# Absolute configuration by vibrational circular dichroism of anti-inflammatory macrolide briarane diterpenoids from the Gorgonian *Briareum asbestinum*

**DOI:** 10.1038/s41598-020-79774-1

**Published:** 2021-01-12

**Authors:** Dawrin Pech-Puch, Pedro Joseph-Nathan, Eleuterio Burgueño-Tapia, Carlos González-Salas, Diana Martínez-Matamoros, David M. Pereira, Renato B. Pereira, Carlos Jiménez, Jaime Rodríguez

**Affiliations:** 1 Centro de Investigacións Científicas Avanzadas (CICA) e Departamento de Química, Facultade de Ciencias, Agrupación Estratéxica CICA-INIBIC, Universidade da Coruña, 15071 A Coruña, Spain; 2 Departamento de Química, Centro de Investigación Y de Estudios Avanzados del Instituto Politécnico Nacional, Apartado 14-740, 07000 Mexico City, Mexico; 3 Departamento de Química Orgánica, Escuela Nacional de Ciencias Biológicas, Instituto Politécnico Nacional, Prolongación de Carpio Y Plan de Ayala, Col. Santo Tomás, 11340 Mexico City, Mexico; 4 Departamento de Biología Marina, Universidad Autónoma de Yucatán, Km. 15.5, Carretera Mérida-Xmatkuil, A.P. 4-116 Itzimná, C.P. 97100 Mérida, Yucatán México; 5 REQUIMTE/LAQV, Laboratório de Farmacognosia, Departamento de Química, Faculdade de Farmácia, Universidade Do Porto, R. Jorge Viterbo Ferreira 228, 4050-313 Porto, Portugal

**Keywords:** Natural products, Structure elucidation

## Abstract

The four new briarane diterpenoids 2-butyryloxybriarane B-3 (**2**), 9-acetylbriarenolide S (**3**), briarenolide W (**4**), and 12-isobriarenolide P (**5**), along with briarane B-3 (**1**) and the five known diterpenes **6–10** were isolated from the gorgonian coral *Briareum asbestinum* collected from the Mexican Caribbean Sea*.* The structures were elucidated by 1D and 2D NMR and MS measurements. Since the structure of briarane B-3 (**1**) was only suggested and published without any spectroscopic support, it was herein confirmed, and the supporting data are now provided. In addition, **1** provided the opportunity to explore the sensitivity of vibrational circular dichroism (VCD) to determine the configuration of a single stereogenic center in the presence of eight other stereogenic centers in a molecule possessing a highly flexible ten-member ring. A single-crystal X-ray diffraction study, in which the Flack and Hooft parameters of **1** were determined, further confirmed that briarane B-3 is (1*S*,2*S*,6*S*,7*R*,8*R*,9*S*,10*S*,11*R*,17*R*)-**1**. This paper reports for first time the use of VCD in briarane diterpenes and with the presence of chlorine atoms. Biological evaluation of seven isolated compounds evidenced a moderate anti-inflammatory activity for compounds **6** and **9** but it did not show any cytotoxic, antiviral, antibacterial, and topoisomerase inhibitory activity.

## Introduction

The prodigious creativity of Nature is evidenced when contemplating the diverse biosynthetic processes of diterpenoids, the largest class of natural products, comprised by more than 80 thousand representatives, which are generated from simple acyclic achiral precursors^1^. Among them, briaranes represent one of the more common families of compounds isolated from marine sources^2,3^. Particularly, the gorgonian *Briareum asbestinum* is a prolific source of this type of diterpenoids^4–7^, whose molecular architecture shows a *trans*-fused bicyclo[8.4.0]tetradecane ring system with hydroxyls, esters, epoxides, and chlorine atoms in their skeletons. Very distinctive is a *γ*-lactone moiety at C-7–C-8, and in many cases congested four-contiguous stereogenic carbon atoms at C-1, C-2, C-10, and C-14 are found.

Since the first structural elucidation of briarein A isolated from *B. asbestinum* in 1977^8^, more than 500 briarane-type secondary metabolites have been reported from Octocorallia Subclass, including Gorgonacea, Pennatulacea, Alcyonacea and Stolonifera Orders. Some of these compounds displayed biological activities such as cytotoxic, antiviral, anti-inflammatory, immunomodulatory, antifouling and ichthyotoxic^9–14^.

**Figure 1:**
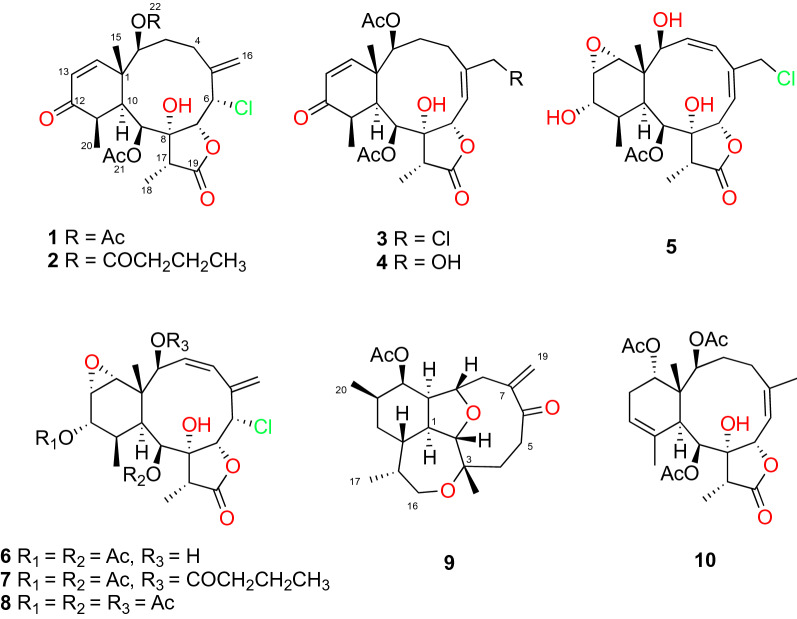
Briarane diterpenes isolated from *Briareum asbestinum*.

In the course of our ongoing chemical research on bioactive marine natural products^15–18^, in particular diterpenes from marine sources^19–24^, and more specifically from those collected in the Yucatan peninsula (Mexico)^25^, we focused our attention on the soft coral *B. asbestinum* because it was assumed to be a rich source of briarane diterpenoids. Thus, in this paper we describe the isolation, structure elucidation, absolute configuration (AC) evaluation, and biological activity searches of the new and known diterpenoids **1**–**10**, found as constituents of this gorgonian, whose structures are shown in Fig. 1.

## Results

### Isolation of metabolites

Specimens of *B. asbestinum*, collected off shore the Yucatan Peninsula, were extracted with CH_2_Cl_2_- MeOH (1:1 *v*/*v*) to give a crude extract that was partitioned using the modified Kupchan procedure^26,27^. The CH_2_Cl_2_ soluble portion was subjected to SiO_2_ flash column chromatography and normal phase HPLC purification using isocratic mixtures of hexanes-acetone as the mobile phase to afford pure samples of **1**–**10**. All compounds could be obtained as pure samples according to their TLC and NMR evaluations.

### Structure and absolute configuration of 1

**Figure 2:**
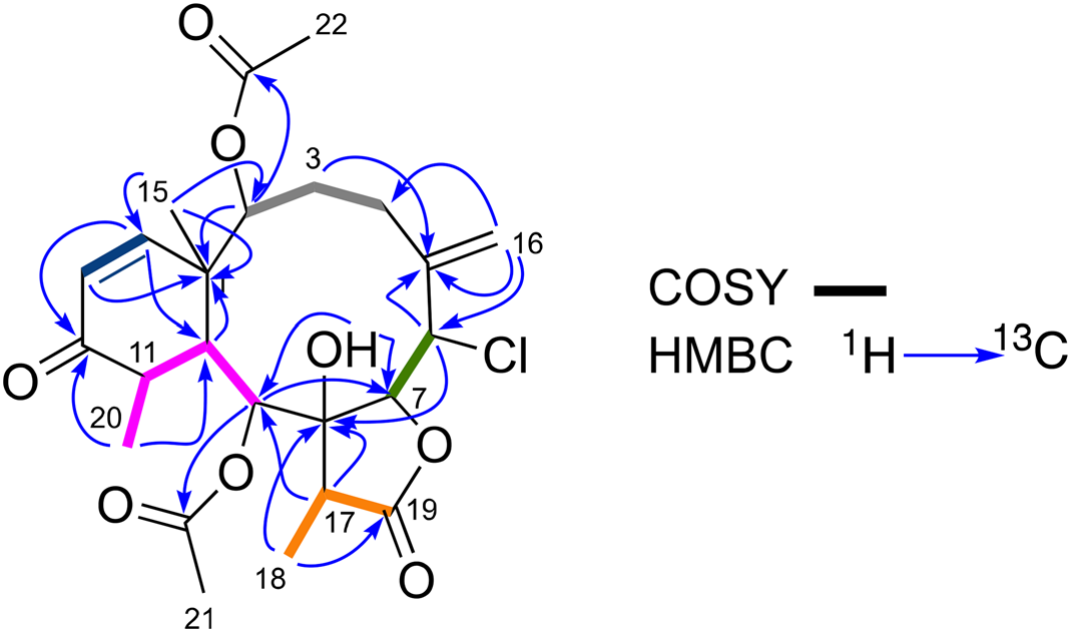
^1^H-^1^H COSY and key HMBC correlations found in **1.** Figure was created with Chemdraw V.20 (see permission in https://informatics.perkinelmer.com/sitesubscription/).

Briarane **1** was isolated as white crystals. The presence of one chlorine atom became evident from the [M + Na]^+^ cluster ion peaks at *m*/*z* 505/507, in a 3:1 ratio, observed in the LRESIMS. The HRESIMS ion peak at *m*/*z* 505.1584 provided the molecular composition [C_24_H_31_O_8_^35^Cl + Na]^+^ (calculated 505.1605), accounting for an index of hydrogen deficiency (IHD) of nine. The IR spectrum showed absorption bands at 3520, 1765, and 1736 cm^−1^, indicating the presence of hydroxy, *γ*-lactone, and ester functionalities, respectively. The ^1^H and ^13^C NMR spectra (Tables 1, 2, respectively), together with the DEPT-135 data, correlated by an edited HSQC experiment, were indicative of a briarane skeleton. The ^1^H-^1^H COSY spectrum displays five separate spin–spin coupling systems that map-out the four hydrogen atom sequences H-2–H-3–H-4–H-16 (through allylic coupling), H-6–H-7, H-9–H-10–H-11–H-20, H-13–H-14, and H-17–H-18 (Fig. 1). These spin–spin systems were connected through the HMBC correlations, thereby confirming the briarane skeleton, as follows: from H-2 to C-1; H-3 to C-5; H-6 to C-5 and C-8; H-9 to C-7 and C-8; H-10 to C-1; H-16 to C-4, C-5 and C-6; and the broad signal at *δ*_H_ 3.43, assigned to a hydroxy group, showed long-range correlations with C-7 and C-9 thereby allowing us to establish the C-1–C-10 connectivity of the ten-member ring. The ring fusion to a methylcyclohexenone, at C-1 and C-10 was evidenced by the HMBC correlations between H-13 and C-1, between H-14 and C-1 and C-12, between H-10 and C-11, between H_3_-20 and C-10, C-11, and C-12, and between H_3_-15 and C-1, C-2, and C-14 (Fig. 2).

**Table 1:** ^1^H NMR data (*δ* in ppm, *J* in Hz) of briaranes **1–5** at 500 MHz in CDCl_3_.

N	1	2	3	4	5
2	4.81 (d, 9.4)	4.82 (d, 9.5)	4.37 (d, 6.0)	4.40 (d, 5.4)	4.27 (d, 9.7)
3	2.41 (dt, 15.6, 9.4, 9.4)	2.40 (m)	2.78 (dt,15.4, 15.4, 5.5)	2.85 (dt, 15.5, 15.5, 4.9)	5.89 (dd, 11.0, 9.7)
	1.87 (dd, 15.6, 10.9)	1.86 (m)	1.77 (m)	1.74 (m)	
4	2.61 (dd, 15.0, 10.9)	2.57 (m)	2.56 (m)	2.55 (m)	6.21 (ddd, 11.0, 1.2)
	1.62 (m)	1.60 (m)	2.05 (m)	2.00 (m)	
6	4.83 (bs)	4.84 (d, 9.3)	5.88 (dd, 10.3, 0.6)	5.72 (d, 10.2)	5.83 (dq, 8.6, 1.2, 1.2, 1.2)
7	5.77 (bs)	5.71 (bs)	5.23 (d, 10.3)	5.28 (d, 10.2)	5.07 (d, 8.6)
9	5.11 (d, 5.1)	5.11 (d, 5.5)	5.28 (d, 4.7)	5.30 (d, 5.2)	5.14 (d, 6.7)
10	2.99 (dd, 4.7, 5.1)	2.99 (dd, 5.0, 5.5)	2.64 (dd, 4.6, 4.7)	2.77 (t, 5.2)	2.12 (d, 6.7)
11	2.61(dq, 4.7, 7.4)	2.61 (m)	2.53 (m)	2.52 (m)	1.80 (m)
12	–	–	–	–	3.94 (dd, 5.9, 2.3)
13	5.97 (d, 10.4)	5.97 (d, 10.5)	5.84 (d, 10.5)	5.80 (d, 10.5)	3.56 (dd, 5.9, 3.6)
14	6.19 (d, 10.4)	6.20 (d, 10.5)	6.40 (d, 10.5)	6.40 (d, 10.5)	3.41 (d, 3.6)
15	1.16 (s)	1.16 (s)	1.22 (s)	1.20 (s)	1.11 (s)
16	5.69 (bs)	5.69 (q, 2.0)	4.32 (d, 12.0)	4.32 (d, 15.8)	4.24 (d, 12.8)
	5.37 (s)	5.37 (bs)	4.18 (d, 12.0)	4.07 (d, 15.8)	4.18 (d, 12.8)
17	2.41 (q, 7.7)	2.45 (q, 7.3)	2.45 (q, 7.2)	2.41 (q, 7.1)	2.31 (d, 7.1)
18	1.30 (d, 7.7)	1.30 (d, 7.3)	1.19 (d, 7.2)	1.19 (d, 7.1)	1.17 (d, 7.1)
20	1.30 (d, 7.4)	1.33 (d, 7.4)	1.30 (d, 7.4)	1.29 (d, 7.4)	1.01 (d, 7.5)
21	2.14 (s)	2.15 (s)	2.25 (s)	2.24 (s)	2.18 (s)
22	2.24 (s)		2.14 (s)	2.14 (s)	
1′		2.45 (q, 7.4)			
2′		1.77 (m)			
3′		1.02 (t, 7.4)			
OH	3.43 (bs)	3.42 (bs)

**Table 2:** ^13^C NMR data (*δ* in ppm) of briaranes **1–5** at 125 MHz in CDCl_3_.

C	1	2	3	4	5
1	44.9	44.8	44.2	44.2	40.4
2	79.3	79.2	79.6	80.6	76.5
3	28.5	28.6	31.7	31.7	137.2
4	28.5	31.7	26.2	25.5	125.0
5	142.6	142.6	144.2	147.8	141.2
6	67.3	67.3	123.3	118.3	126.7
7	78.2	78.3	77.4	77.5	79.2
8	82.98	83.0	82.6	82.4	82.5
9	74.4	73.5	71.1	71.2	70.0
10	38.5	38.5	38.3	37.9	31.3
11	46.8	46.9	48.6	48.1	41.7
12	202.7	202.8	203.1	203.9	66.7
13	126.1	126.0	124.3	124.2	55.2
14	154.7	154.9	154.8	155.4	64.4
15	19.0	19.0	15.4	15.4	14.7
16	119.9	120.0	51.2	67.5	46.6
17	42.3	42.3	42.6	42.8	43.2
18	10.1	14.0	6.8	6.8	6.5
19	176.4	175.9	175.7	176.7	175.7
20	15.1	15.2	15.2	15.3	13.3
21COO	170.3	170.4	169.0	169.1	169.9
22COO	169.6	172.6	170.7	171.1	
CH_3_21	21.9	21.9	21.3	21.2	21.9
CH_3_22	21.2		21.9	21.9	
OH					
1´CH_2_		36.3			
2´CH_2_		18.5			
3´CH_3_		14.3

The γ-lactone was located on the briarane scaffold by the HMBC correlations between H_3_-18 and C-8 and the γ-lactone C-19 carbonyl signal at δ_C_ 176.4, and between H-17 and C-8 and C-9. In addition, the presence of two acetate groups was evidenced by two methyl singlets at δ_H_ 2.14 (δ_C_ 21.9) and δ_H_ 2.24 (δ_C_ 21.2) along with the carbonyl signals at δ_C_ 170.3 and 169.6. The HMBC correlations between these carbonyl signals and the methine doublets at δ_H_ 4.81 and 5.11, respectively, (Fig. 2), defined the positions of the acetate groups at C-2 and C-9. The NMR data of **1** were similar to those of solenolide E^28^ in which C-9 has a hydroxy group.

SciFinder and MarinLit databases searches revealed that the (1*S*,2*R*,6*S*,7*R*,8*R*,9*R*,11*R*,17*R*)-**1** stereostructure was suggested (no stereochemistry at C-10), based on undisclosed NMR methods, for an isolate named as briarane B-3^29^. The compound was found in the same coral, also collected from the Caribbean Sea, although at several West Indies locations instead from a mainland location. The C-10 stereogenic center was not drawn at the time^29^, but it was recently assumed^30^ as (10*S*) from the lack of a NOE effect of the angular methyl group and H-10, in agreement with a *trans* ring fusion present in similar briaranes^31^. A (1*S*,2*S*,6*S*,7*R*,8*R*,9*R*,10*S*,11*R*,17*R*) stereochemistry was assumed based on extensive NMR studies including NOESY, *J*-HMBC, and HSQC-HECADE measurements^30^. Other observed NOE effects that can be considered as secure for **1** are those of the methyl groups at C-1 and C-11 which have an 1,3-*diaxial* distribution on a cyclohexenone, as well as that seen for H-7 and H-17 which are *syn* distributed on the *γ*–lactone, although in this case the observation is not defining the relative stereochemistry of H-7 and H-10. In contrast, a NOE effect observed for H-2 and H-10 has to be interpreted with care since either stereochemistry at C-2 is feasible according to the conformational freedom of the ten-member ring, which is evident after assembly and manipulation of a solid Dreiding stereomodel from Büchi (Flawil, Switzerland), as was done to understand the uncommon conformational behavior of the diterpenoid icetexone^32^. In addition, there is no useful NOE effect observation to define the C-9 stereogenic center.

The stereochemistry of **1** could be expected as that of solenolide E^28^, although, as mentioned above, there appeared to be uncertainty for the configuration of the C-2 and C-9 stereogenic centers. Since the absolute configuration (AC) of **1** could be verified at the same time using an independent methodology, vibrational circular dichroism (VCD) was selected for this purpose. This methodology has shown to be a powerful tool^33^ for the AC determination of many types of natural products^34^, but not yet for briaranes. It was also successful for the determination of one stereogenic center in the presence of nine other stereogenic centers, as occurred for the epoxidation reaction study of the pentacyclic triterpenoid lupenone^35^.

We begin with IR and VCD spectra calculation for the (1*S*,2*R*,6*S*,7*R*,8*R*,9*R*,10*S*,11*R*,17*R*) diastereoisomer by constructing a molecular model in the Spartan 04 software. This model was submitted to conformational population searches using the Monte Carlo protocol and Merck Molecular Force Field (MMFF)^36^ without any conformational restriction, in particular since we are dealing with a molecule possessing a ten-member ring. This search provided 25 conformers in a 9.93 kcal/mol energy window for which the single point (SP) energy was calculated at the DFT B3LYP/6-31G(d) level of theory using the same software. The five conformers found in the initial 5 kcal/mol energy gap, were optimized at the DFT B3LYP/DGDZVP level of theory using the Gaussian 09 software to render three conformers contributing each with more than 1% to the total conformational distribution. These minimum energy structures were verified for the absence of imaginary frequencies, they are shown in Figure S49 (Supplementary Information), and their thermochemical data are summarized in Table S3 in Supplementary Information. These three conformers were used for the IR and VCD frequency calculations at the same level of theory, also in the Gaussian 09 suit. Conformer population weighting was done according to the Δ*G* =  − *RT* ln *K* equation to generate the respective Boltzmann averaged IR and VCD spectra.

The IR and VCD spectra of the (1*S*,2*R*,6*S*,7*R*,8*R*,9*R*,10*S*,11*R*,17*R*) diastereoisomer were compared to the experimental IR and VCD spectra of **1** (Figure S50 in Supplementary Information) using the statistic correlation of the Compare*VOA* program^37^. The confidence level data are summarized in Table S2 in Supplementary Information, where the optimal anharmonicity factor (*anH*) was 0.972. The IR similarity (*S*_IR_) was relatively poor (89.3) and the VCD similarity for the assumed stereochemistry (*S*_E_) was only 24.7, while that for the other enantiomer (*S*_−E_) was 45.3, providing a very poor Enantiomer Similarity Index (*ESI*) of − 220.6, which is obtained as the *S*_E_ − *S*_−E_ difference. The 51% confidence level (*C*) for the (1*S*,2*R*,6*S*,7*R*,8*R*,9*R*,10*S*,11*R*,17*R*) AC of **1** clearly showed the relative stereochemistry was incorrectly assumed.

Since a NOE effect for H-10 and H-2 was seen, but no effect was evident for H-9, it was next assumed this stereogenic center might be inverted. Thus, a similar calculation protocol was undertaken for the (1*S*,2*R*,6*S*,7*R*,8*R*,9*S*,10*S*,11*R*,17*R*) diastereoisomer. The MMFF search provided 11 conformers, in a 9.06 kcal/mol gap, which were submitted to SP calculations. The six conformers in the initial 5 kcal/mol energy gap, were DFT B3LYP/DGDZVP optimized to render the four conformers shown in Figure S51 (in Supplementary Information) contributing each with more than 1% to the conformational distribution. The conformers, whose thermochemical parameters are summarized in Table S2 in Supplementary Information, were used for the IR and VCD frequency calculations and also to generate the IR and VCD spectra. A spectrum contrasting procedure using the Compare*VOA* software provided the data given in Table S3 while the plots are shown in Figure S52 (in Supplementary Information).

**Figure 3:**
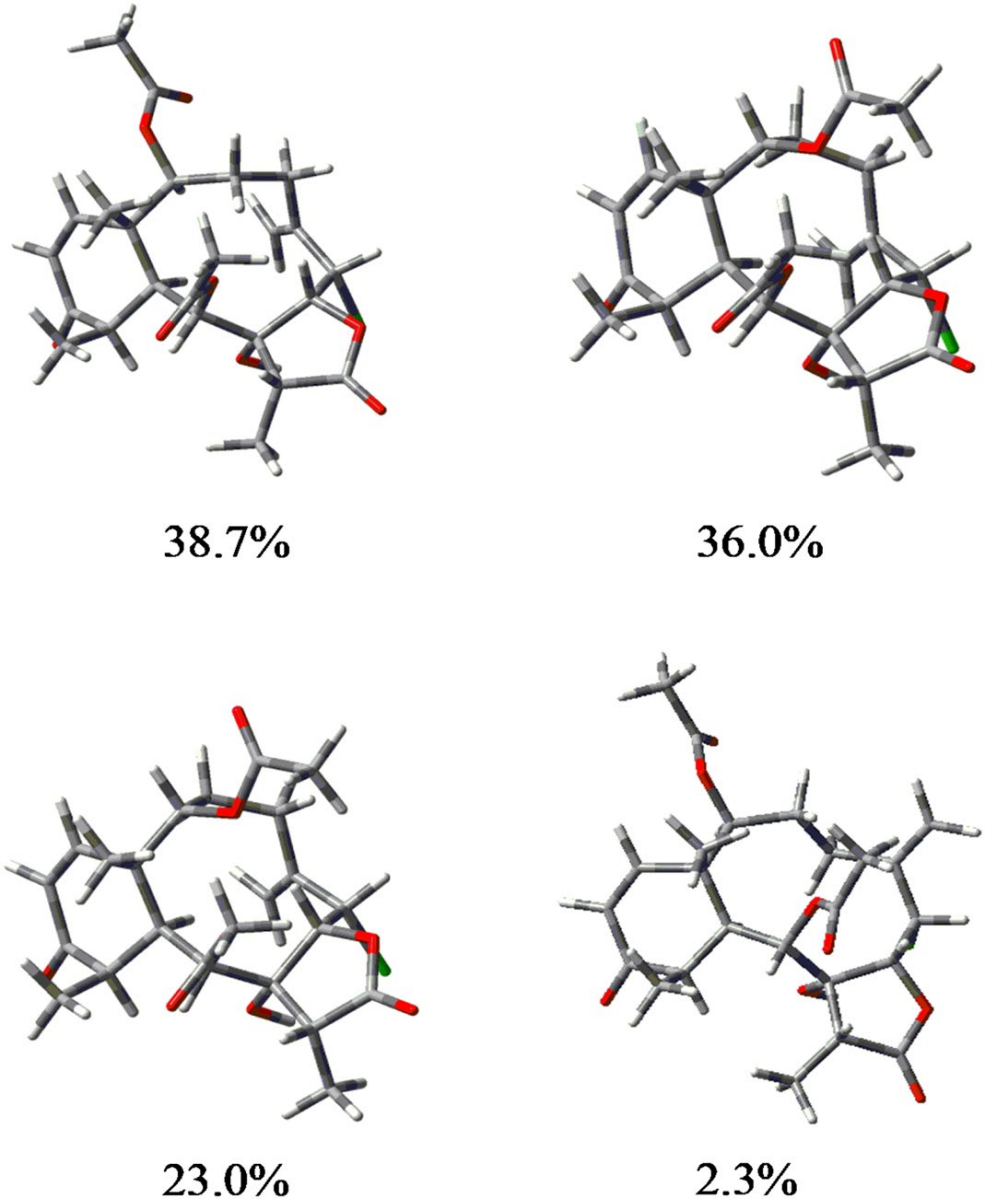
The four most stable conformers of briarane B-3 (**1**). Figure was created with Gaussview v 6.0.

A good data improvement revealed the (9*S*) configuration is indeed evident since *S*_IR_ improved to 93.3, *S*_E_ almost duplicated to 48.9, *S*_−E_ severely decreased to 25.6, and *C* increased to 62%. The above results suggested the C-2 configuration should also be inverted. MMFF calculation of the (1*S*,2*S*,6*S*,7*R*,8*R*,9*S*,10*S*,11*R*,17*R*) diastereoisomer provided 52 conformers in a 9.98 energy gap which after SP calculations left seven conformers in the initial 5 kcal/mol gap. Energy optimizations, as above, rendered the four conformers shown in Fig. 3 which reveal the high conformational freedom of the ten-member ring, while the corresponding thermochemical data are summarized in Table S2 in Supplementary Information.

**Figure 4:**
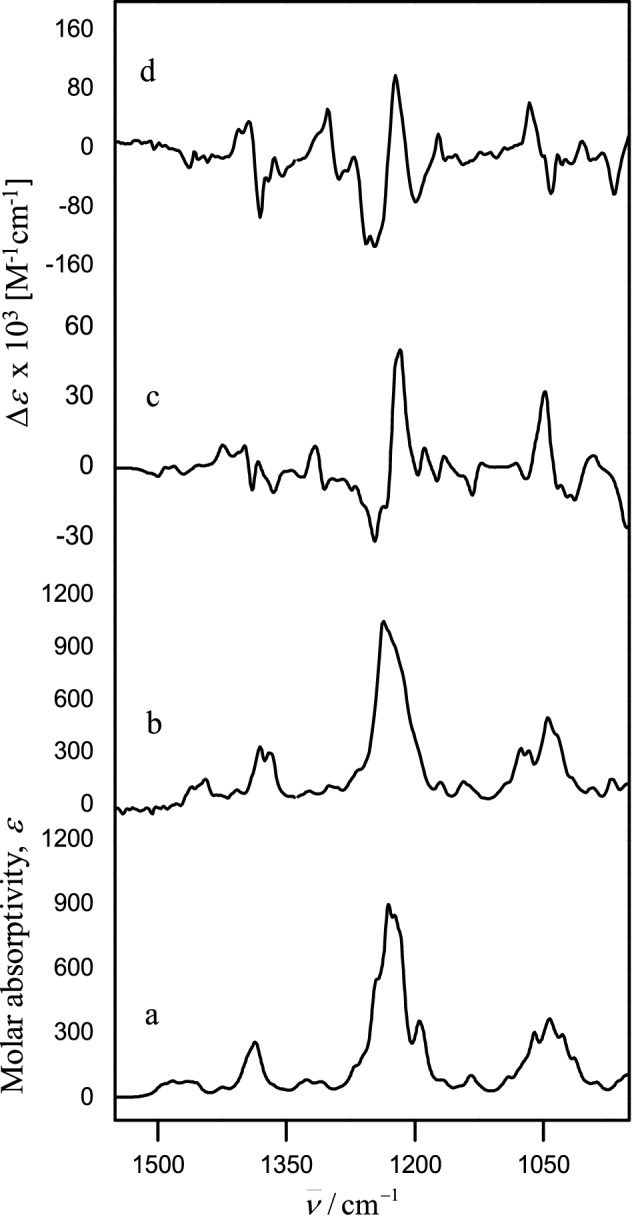
Comparison of the experimental IR (**b**) and VCD (**d**) spectra of briarane B-3 (**1**) with the DFT B3LYP/DGDZVP calculated IR (**a**) and VCD (**c**) spectra for (1*S*,2*S*,6*S*,7*R*,8*R*,9*S*,10*S*,11*R*,17*R*)-**1.**

These four conformers were used to calculate the IR and VCD spectra of the (1*S*,2*S*,6*S*,7*R*,8*R*,9*S*,10*S*,11*R*,17*R*)-diastereoisomer. Compare*VOA* contrasting of the calculated and experimental spectra revealed that the two acetates of **1** are *beta* oriented and have the (*S*) absolute configuration since *S*_IR_ climbed to an excellent 97.3 value, *S*_IR_ now is 78.0, and the confidence level (*C*) is 100%. The corresponding spectra are shown in Fig. 4.

To complete the study for determining the C-2 and C-9 stereochemistry, the fourth possibility, the (1*S*,2*S*,6*S*,7*R*,8*R*,9*R*,10*S*,11*R*,17*R*)-diastereoisomer was also calculated as above. The 41 conformers obtained in a 9.77 kcal/mol gap, after MMFF searches, were reduced to nine in a 5 kcal/mol gap after SP calculations.

The thermochemical parameters of the three final contributing conformers are given in Table S2 in Supplementary Information and the Compare*VOA* data are summarized in Table S3, while the conformers are shown in Figure S53 (Supplementary Information) and the spectra comparison in Figure S54 (Supplementary Information). Close inspection of Table S3 shows that the (2*S*,9*R*) and (2*R*,9*S*) data sets provide similar *S*_IR_ and *C* values since in either case one stereogenic center is incorrectly assumed. In addition, *ESI* is negative in the (2*S*,9*R*) case since the *S*_-E_ value is larger than the *S*_E_ value, indicating that the C-2 and C-9 stereogenic centers highly influence the overall VCD spectroscopic behavior. It also follows from the VCD study of the four C-2 and C-9 diatereoisomers that the stereostructure originally suggested for (1*S*,2*S*,6*S*,7*R*,8*R*,9*S*,10*S*,11*R*,17*R*)-**1** is indeed correct and it is evident that VCD is a very sensitive methodology to establish the absolute configuration (AC) of a single stereogenic center, located on a flexible macrocycle, in the presence of eight other stereogenic centers. To our knowledge, this is the first report of the use of VCD in briarane diterpenes, and also with a natural compound possessing a chlorine atom.

**Figure 5:**
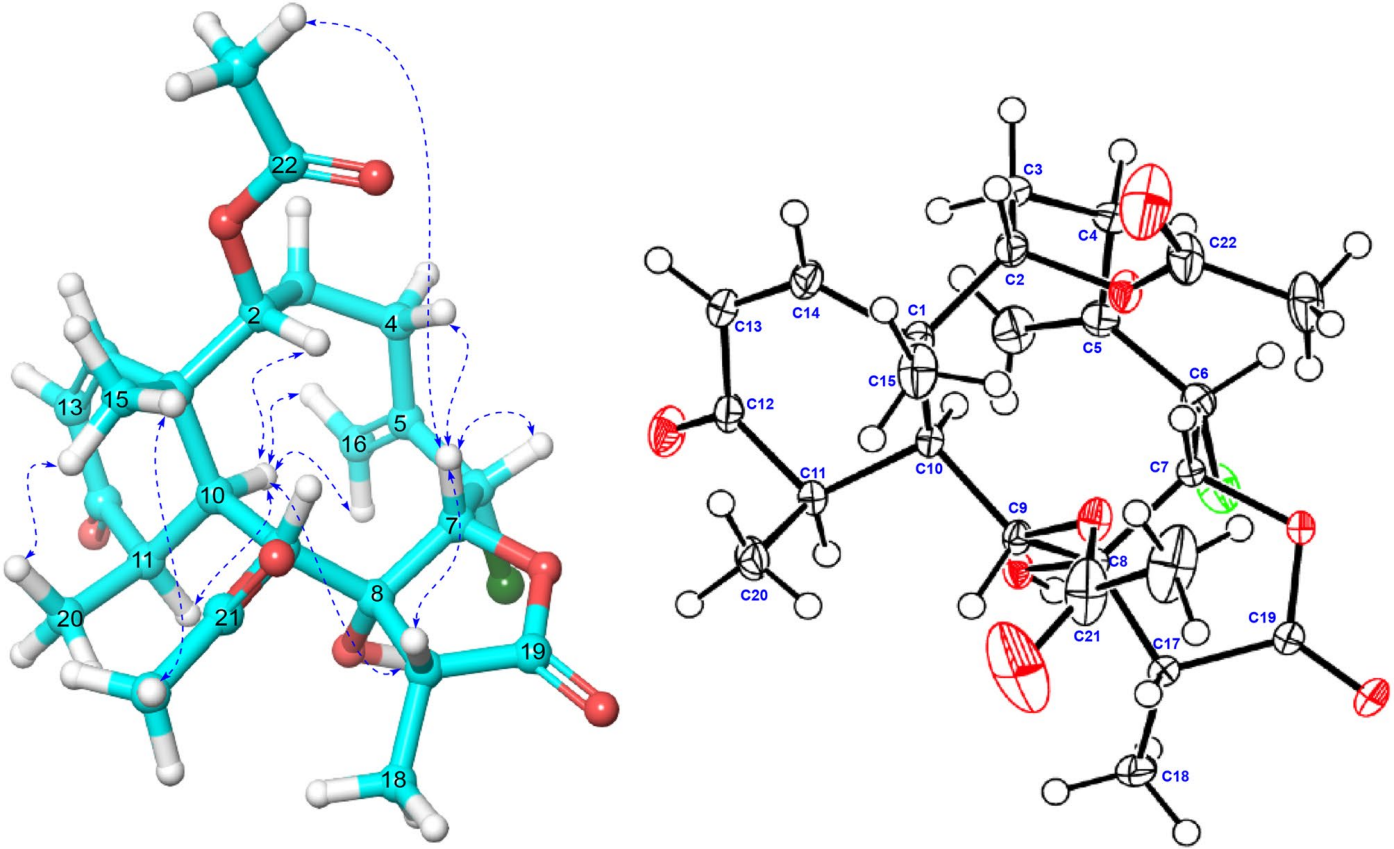
(**a**) Key NOESY correlations observed in compound **1**. (**b**) ORTEP X-Ray representation of compound **1**.

The absolute configuration of **1** was independently confirmed in the present study by single-crystal X-ray diffraction (XRD). This method has been used successfully a long time ago for briaranes from *B. asbestinum* when they possess a halogen atom, like briarein A^8^. In that case the chlorine atom scattering anomalous dispersion was used to measure differences in Bijvoet intensities, allowing to establish the AC of the compound. In the present case, two independent measurements were done. One XRD study, performed at 100 K, allowed to refine the data to a convergence value R = 3.5% as detailed in the Experimental Section. In the second XRD study the data were acquired at room temperature for the complete diffraction sphere, thereby allowing determining the AC by means of the the Flack^38^ and Hooft^39^ parameters. The R value was 4.8%, while the Olex2 software^40^ provided the Flack and Hooft parameters for (1*S*,2*S*,6*S*,7*R*,8*R*,9*S*,10*S*,11*R*,17*R*)-**1** as x =  − 0.010(13) and *y* = 0.007(10), respectively, which for the inverted structure were *x* = 1.003(13) and *y* = 1.012(10), respectively. Figure 5 depicts the AC on an ORTEP drawing for **1**.

The absolute determination of **1** by VCD and XRD is in agreement with a very recent ^1^H residual chemical shift anisotropy measurement^41^, a powerful contemporary methodology for the study of molecular structures^42^.

### Structure elucidation of 2–10

Compound **2** was also isolated as an amorphous white powder. Its molecular formula of C_26_H_35_ClO_8_ was deduced from its ( +)-HRESIMS. The IR spectrum and the ^1^H- and ^13^C-NMR data (Tables 1, 2) were very similar to those of **1**, but they displayed that one of the acetate groups in **1** had been replaced by a butyrate moiety in **2**. This was suggested from its ^13^C-NMR spectrum that shows two ester-type carbonyl carbons (δ_C_ 170.4 and 172.6) and from its ^1^H-NMR spectrum showing only one acetate methyl signal at δ_H_ 2.15 and an additional spin system consisting of a methyl triplet signal at δ_H_ 1.03 correlated by ^1^H-^1^H COSY to a multiplet at 1.77 (m), and this in turn to another multiplet centered at 2.45 (m). Two additional *sp*^*3*^ methylenes observed in the ^13^C-NMR of **2** and the increase of 28 uma of its molecular weight in relation to that of **1** confirmed the substitution of one of the two acetates in **1** for a butyrate group in **2**. The relative configuration of **2** was displayed to be the same as that of **1** by the identical NOE correlations (Figure S55, Supporting Information) observed in its NOESY experiment, the similar proton-proton coupling constants in its ^1^H-NMR and by comparison of the NMR data of **2** with those of **1** (Tables 1, 2). The similar optical rotation of **2** in relation to that of **1** suggests that they share the same absolute configuration. So, we named compound **2** as 2-butyryloxybriarane B-3.

The C_24_H_31_ClO_8_ molecular composition of **3**, isolated as an amorphous white powder, followed from its HRESIMS which showed the [M + Na]^+^ ion at *m*/*z* 505.1596 (calcd for C_24_H_31_ClO_8_Na, 505.1605). This composition is the same as that of **1** and therefore both compounds seem to be isomers. Comparison of the ^1^H NMR spectra of **1** and **3** (Table 1) revealed two relevant changes: instead the exocyclic methylene signals of **1** at *δ*_H_ 5.69 and 5.37, there is only one vinyl proton signal which appears as a double doublet (*J* = 10.3, 0.6 Hz) at *δ*_H_ 5.88 in the spectrum of **3**, and instead the hydrogen atom geminal to chlorine at *δ*_H_ 4.84 in **2**, there is an *sp*^3^ methylene group which appears as two doublets (*J* = 12.0 Hz) at δ_H_ 4.32 and 4.18 suggesting the Δ^5(16)^ double bond of **1** switched to Δ^5^ in **3**. This allylic relationship of the double bond and the chlorine atom in **1** and **3** is in further agreement with a ^13^C NMR data (Table 2) comparison. The double bond of **1** appears as a non-protonated signal at *δ*_C_ 142.6 (C-6) in **1** which is shifted to *δ*_C_ 144.2 in **3**, while the methylene signal of **1** at *δ*_C_ 120.0 (C-16) is replaced by a methine signal at *δ*_C_ 123.3 (C-6) in **3** and the methine chlorine bearing atom in **1** at *δ*_C_ 67.3 (C-6) is replaced by a menthylene group at *δ*_C_ 51.2 (C-16). The NMR data of **3** are also in agreement with those of briarenolide S^31^. NOE correlation of olefinic H-6 and the methylene H_2_-16 atoms suggested the *E* geometry of the Δ^5^ double bond in the macrocycle of **3**. Comparison of cross-peaks in the NOESY plots of** 3** with those of **1** and **2** suggest the three molecules share a common relative configuration (Figure S56 in Supplementary Information). We named **3** as 9-acetylbriarenolide S.

Compound **4** was also isolated as an amorphous white powder and the molecular formula was assigned as C_24_H_32_O_9_ in accordance to the HRESIMS [M + Na]^+^ ion at *m*/*z* 487.1953 (calculated for C_24_H_32_O_9_Na, 487.1944). This composition, when compared to that of **3**, suggested that in **4** a hydroxy group replaced the chlorine atom of **3**, a fact that is confirmed since the ^1^H and ^13^C NMR spectra of **4** are very similar to those of **3**, the main differences being due to the methylene group signals at position 16. While the two doublets (*J* = 12.0 Hz) appear at *δ*_H_ 4.32 and 4.18 in **3**, they appear at *δ*_H_ 4.32 and 4.07 (*J* = 15.8 Hz) in **4** (Table 1) and the C-16 signal in **3**, at *δ*_C_ 51.2, is shifted to δ_C_ 67.5 in **4** (Table 2). The relative configuration of **4** at C-1, C-2, C-7, C-8, C-9, C-10, C-11, and C-17 is the same than in **1**–**3** as evidenced by similar correlations observed in its NOESY plot (Figure S57 in Supplementary Information). We have named **4** as briarenolide W.

**Figure 6:**
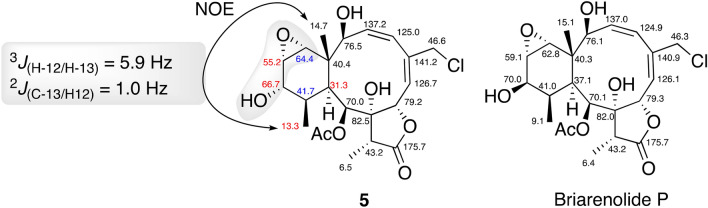
Comparison of the ^13^C NMR data between **5** and briarenolide P^27^ (carbon chemical shift differences: Δδ_C_ > 3 ppm in red and Δδ_C_ between 0.5 to 1.8 ppm in blue), key coupling constants (see reference^18^) and NOE correlations in **5.** Figures were created with Chemdraw 20.0 and Chem3D v.2020.

Briarane **5** was also isolated as an amorphous white powder. The isotopes clusters at *m*/*z* 479/481 in a 3:1 ratios corresponding to [M + Na]^+^ ion peak revealed that **5** also had a chlorine atom. The HRESIMS of **5** at *m*/*z* 479.1437 (calculated 479.1449), indicated the C_22_H_29_ClO_8_ molecular composition with an IHD of eight. Comparison of the IR, ^1^H, and ^13^C NMR data of **5** showed they were very similar to those of briarenolide P^31^, the main differences being found for the six-member ring, and remarkably for the C-20 methyl signal. The strong NOE effect of H_3_-15 and H_3_-20 indicated that they are on the same face of the molecule. The shift of C-20 from *δ*_C_ 9.1 in briarenolide P to *δ*_C_ 13.3 in **5** suggested a smaller *γ*-gauche effect due to an 1,3-diaxial interaction between these two methyl groups in **5** as compared to that occurring in briarenolide P^31^. This suggests a conformational change of the six-member ring, probably due to the different configuration of the hydroxy group at C-12 (see ^13^C chemical shifts in red and blue colors in Fig. 6).

Thus, the relative stereochemistry of the hydroxy and epoxy groups at C-12, C-13, and C-14 in **5** followed from evaluation of their ^1^H NMR coupling constants, the heteronuclear carbon-proton coupling constants in a HSQC-HECADE experiment, and its NOESY experiment, using our reported^43^ approach for the evaluation of 3*β*,7-dihydroxy-5,6-epoxycholestanes. The ^3^*J*_H-12/H-13_ = 5.9 Hz and ^3^*J*_C-13/H-14_ = 1 Hz values in **5** implied the *cis* disposition of the hydroxy and epoxy groups. Besides, the strong NOESY cross peak observed between Me-20 and H-12 places both protons on the same face of the ring. The almost identical proton and carbon chemical shifts and proton-proton coupling constants observed in the NMR spectra of **5** and briarenolide P (see black colored carbon chemical shifts in Fig. 6), suggests that they have the same relative configuration at all other stereogenic centers. It thus followed that **5** is the C-12 epimer of briarenolide P, and therefore we named it as 12-isobriarenolide P.

Five known diterpenoids were identified as brianthein X (**6**)^44–46^, brianthein Y (**7**)^44^, brianthein Z^44,47^, (**8**) and asbestinin-10 (**9**)^48,49^, isolated from *B. asbestinum* by comparison of the [*α*]_D_^25^, MS, ^1^H-, ^13^C-NMR and X-Ray information (just for **7)**, while the NMR and MS data of **10** matched with that of lactone 14, isolated from the sea pen *Stylatula* sp. reported by Wratten and Faulkner^50^.

### Anti-inflammatory activity

In order to assure the lack of toxicity of the molecules before more detailed biological studies, the seven diterpenes (compounds **1**, **4**, **6–10**) were initially assed for their potential toxicity against human non-cancer cells, namely keratinocytes (HaCaT). At the highest concentration tested (100 µM) no toxicity was detected (Figure S48 in Supplementary Information).

**Figure 7:**
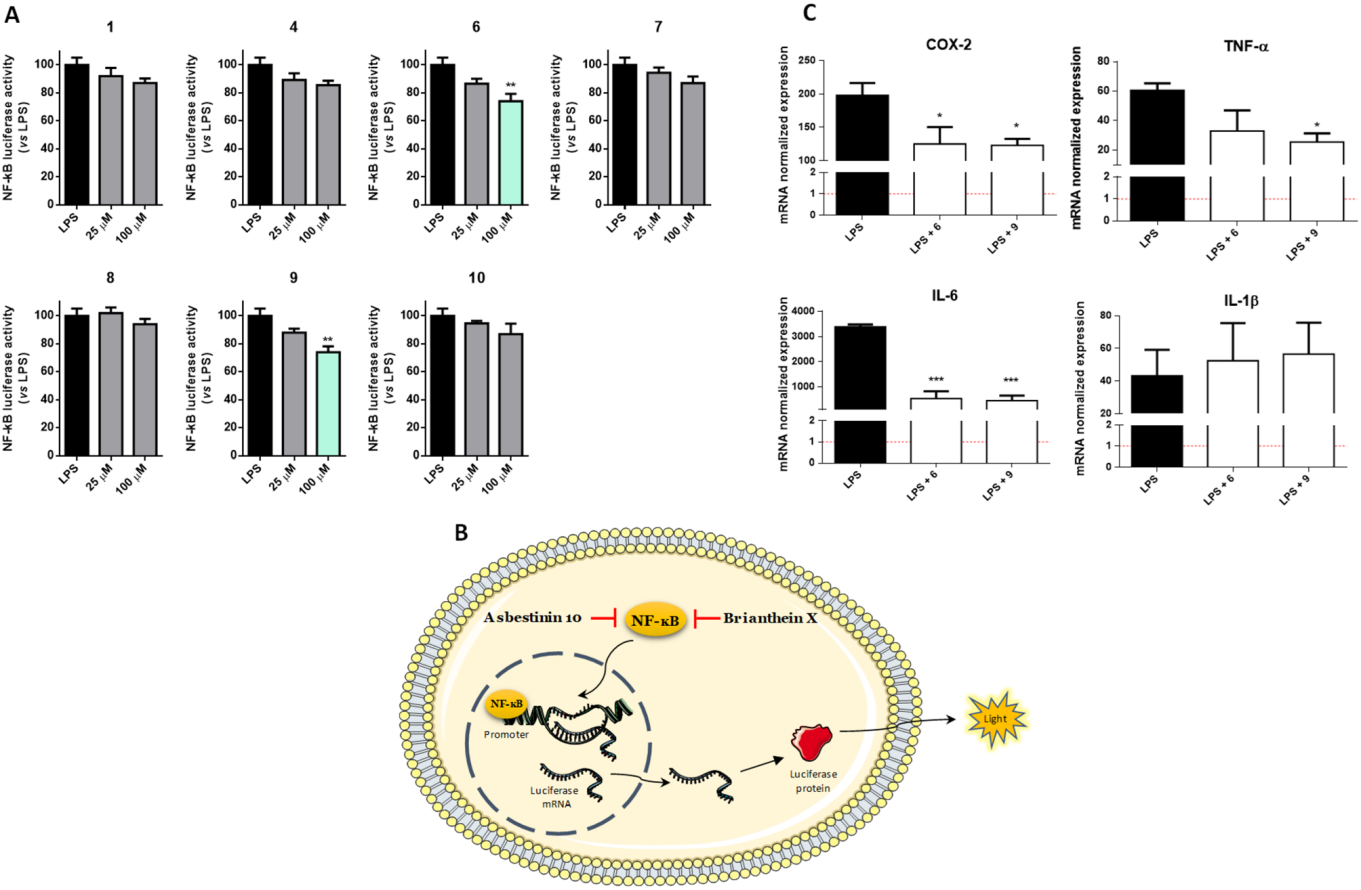
(**A**) NF-κB activation in LPS-challenged THP-1 Lucia cells in the presence of compounds **1**, **4**, **6–10** for 24 h. Incubation with LPS corresponds to maximum NF-κB response, with molecules capable of inhibiting the activation of the transcription factor (**6** and **9**) presenting lower activity values. (**B**) Experimental model used for the assessment of NF-κB in THP-1 luciferase-transfected cells. (**C**) COX-2, IL-6, TNF-α and IL-1β mRNA expression in THP-1 cells. mRNA expression was determined by qPCR after normalization with GAPDH reference gene. Horizontal dashed red line corresponds to the basal expression of the gene in untreated cells. *p < 0.05; **p < 0.01; ***p < 0.001.

Posteriorly, the diterpenes were evaluated for their anti-inflammatory capacity, as assessed for their ability to inhibit or lower the activation of the nuclear transcription factor NF-κB (Fig. 7A). The experimental model used consisted of human THP-1 monocytes stably transfected with a NF-κB-inducible Luc reporter construct (Fig. 7B). Cells were then differentiated into macrophages by incubation with phorbol-myristate acetate (PMA) for 24 h, after which the M1 phenotype was induced by exposure to lipopolysaccharide (LPS). Preliminary studies evaluated the impact of the diterpenes in the viability of these cells, in order to ensure that only non-toxic concentrations were used. As shown in Figure S48, no toxicity was detected at 100 µM. All molecules were evaluated for their impact in NF-κB signaling at two concentrations, 25 and 100 µM. As it can be seen in Fig. 7A, compounds **6** and **9** significantly reduced NF-κB activation at the highest concentration tested.

Considering that the NF-κB transcription factor is considered a master switch in controlling the inflammatory cascade, the capacity of compounds **6** and **9** to lower its activation status is of interest, as these molecules can be further studied as leads for anti-inflammatory drugs. To the best of our knowledge, briarane diterpenes have been described before as anti-inflammatory agents in mouse cells^51^ ours being the first study to describe such effect in human macrophages.

Seeing these results, we were interested in investigating which downstream genes could be modulated by **6** and **9** to exert their anti-inflammatory activity. In Fig. 7C we present the normalized expression of mRNA for COX-2, IL-6, IL-1β and TNF-α. As shown in the results, **6** and **9** significantly decrease the expression of COX-2 and IL-6, with** 9** displaying the same activity towards TNF-α.

All the isolated compounds were also submitted to additional biological evaluations including cytotoxic, topoisomerase I and antibacterial activities. They did not show significant cytotoxic activity against the human tumor cell lines (MDA-MB-231 (breast), HT-29 (colon), NSCLC A549 (lung) and PSN1 (pancreas)); topoisomerase inhibitory activity against the human topoisomerase I, nor antibacterial activity against *Acinetobacter baumannii*,* Pseudomonas aeruginosa*,* Klebsiella pneumoniae*, and *Staphylococcus aureus*.

The insecticidal activity of compound **6** was previously reported^44,52^. The cytotoxic activity and neuroactivity of compound **9** was previously reported^48,53^.

## Conclusions

In summary, the four new briarane diterpenoids, 2-butyryloxybriarane B-3 (**2**), 9-acetylbriarenolide S (**3**), briarenolide W (**4**), and 12-isobriarenolide P (**5**), together with previously suggested briarane B-3 (**1**), the briantheins **6**–**8**, asbestinin (**9**), and diterpenoid **10** were found in the gorgonian *Briareum asbestinum* collected from the Mexican Caribbean Sea. The originally suggested structure and stereochemistry of **1** was established by a combination of NMR measurements and VCD spectroscopy in combination with DFT calculations for first time for this type of compounds. In fact, due to the high conformational flexibility of the ten-member ring of **1**, which difficults the relative configuration determination of several stereogenic centers by means of NMR methods, allows to suggest that VCD might be the method of choice for the determination of the relative configuration of specific stereogenic centers on a ten-member ring when crystals suitable for X-ray diffraction studies could not be gained. VCD has the additional advantage that it provides the absolute configuration of the studied molecule. Several biological tests using the isolated metabolites revealed a mild anti-inflamatory activity.

## Methods

### General experimental procedures

Optical rotations were measured on a JASCO DIP-1000 polarimeter, with a Na (589 nm) lamp and filter. IR spectra were measured on a FTIR Bruker Vector 22 spectrometer. ^1^H, ^13^C, and 2D NMR spectra were recorded on a Bruker Avance 500 spectrometer at 500 and 125 MHz, respectively, using CDCl_3_. Low resolution electrospray mass spectrometry (LRESIMS) and high-resolution electrospray mass spectrometry (HRESIMS) experiments were performed on the Applied Biosystems QSTAR Elite system. The chemical shifts were given in *δ* (ppm) and coupling constants in Hz. HPLC separations were performed on the Agilent 1100 liquid chromatography system equipped with a solvent degasser, quaternary pump, and diode array detector (Agilent Technologies, Waldbronn, Germany) using a semipreparative normal phase column Nova-pak, silica 6 µm 60 Å, 300 × 7.8 mm (Waters). Precoated silica gel plates (Merck, Kieselgel 60 F254, 0.25 mm) were used for TLC analysis, and the spots were visualized under a UV light (254 nm) or by heating the plate pre-treated with H_2_SO_4_/H_2_O/AcOH (1:4:20)^25^.

### Animal material

The gorgonian, *Briareum asbestinum*, was collected in the coast of Mexican Caribbean, Rio Indio, Quintana Roo (18° 84′ 75.61″ N / 87° 65′ 04.55″ W) by scuba diving at a depth of 10 m, in march 2017. The fresh gorgonian was immediately frozen after collection and kept at − 20 °C until being processed. A voucher specimen 17YUBA1 was deposited in the collection of Marine Biology of Autonomous University of Yucatan, Yucatan, Mexico.

### Extraction and isolation

Sliced bodies of *Briareum asbestinum* (wet weight, 1.37 kg; dry weight, 513.70 g) were exhaustively extracted with a mixture of CH_3_OH-CH_2_Cl_2_ (1:1, 3 × 1.5L) at 25 °C for 24 h each extraction, and the extracts were combined and concentrated under vacuum. Following the modified Kupchan methodology, the dark green crude residue (20.0 g) was first partitioned between CH_2_Cl_2_/H_2_O (1:1 *v*/*v*). Then, the organic phase was concentrated under reduced pressure and partitioned between 10% aqueous MeOH (400 mL) and hexane (2 × 400 mL). The H_2_O content (% *v*/*v*) of the methanolic fraction was adjusted to 50% aqueous MeOH and this mixture was extracted with CH_2_Cl_2_ (100 mL). The CH_2_Cl_2_-soluble portion was subjected to flash column chromatography using silica gel and a gradient of n-hexane/EtOAc (0–100%) to obtain 14 fractions (C1-C14).

Fraction C9-C11 was separated by NP-HPLC using an isocratic mixture of 80:20 hexane–acetone as the mobile phase, to obtain compounds asbestinin-10 (**9**, 10 mg), **2** (1 mg), lactone-14 (**10**, 4 mg), **3** (2 mg), **1** (25 mg) and brianthein Z (**8**, 10 mg). Crystallization of fraction C10H8 (*n*-hexane/EtOAc, 4:1) furnished brianthein Y (**7**) (8 mg). Fractions C12 and C13 were purified by NP-HPLC using an isocratic mixture of 60:40 hexane–acetone as the mobile phase, to obtain compounds brianthein X (**6**, 11 mg), **4** (2 mg) and **5** (2 mg).

#### Briarane B-3 (**1**)

Colorless white powder; [α]_D_^25^ + 36.50 (*c* 0.1, MeOH); IR ν_max_ 3520, 2980, 1765, 1736, 1475, 1225, 1050, 900, 725 cm^−1^; ^1^H-NMR data (500 MHz, CDCl_3_), see Table 1; ^13^C-NMR data (125 MHz, CDCl_3_), see Table 2; HRESIMS *m*/*z* 505.1584 [M + Na]^+^ (calcd. For C_24_H_31_ClO_8_Na, 505.1605).

#### 2-Butyryloxybriarane B-3 (**2**)

Colorless amorphous white powder; [α]_D_^25^ + 33.20 (*c* 0.1, MeOH); IR ν_max_ 3520, 2925, 1765, 1675, 1350, 1210, 1025, 950, 725 cm^−1^; ^1^H-NMR data (500 MHz, CDCl_3_), see Table 1; ^13^C-NMR data (125 MHz, CDCl_3_), see Table 2; HRESIMS *m*/*z* 533.1910 [M + Na]^+^ (calcd. For C_26_H_35_ClO_8_Na, 533.1918).

#### 9-Acetylbriarenolide S (**3**)

Colorless amorphous white powder; [α]_D_^25^ − 35.10 (c 0.1, MeOH); IR ν_max_ 3520, 2925, 1800, 1765, 1675, 1350, 1210, 1025, 950, 725 cm^−1^; ^1^H-NMR data (500 MHz, CDCl_3_), see Table 1; ^13^C-NMR data (125 MHz, CDCl_3_), see Table 2; HRESIMS *m*/*z* 505.1596 [M + Na]^+^ (calcd. For C_24_H_31_ClO_8_Na, 505.1605).

#### Briarenolide W (**4**)

Colorless amorphous white powder; [α]_D_^25^ − 34.40 (c 0.1, MeOH); IR ν_max_ 3500, 2925, 1765, 1675, 1350, 1220, 1025, 950, 725 cm^−1^; ^1^H-NMR data (500 MHz, CDCl_3_), see Table 1; ^13^C-NMR data (125 MHz, CDCl_3_), see Table 2; HRESIMS m/z 487.1953 [M + Na]^+^ (calcd. For C_24_H_32_O_9_Na, 487.1944).

#### 12-isobriarenolide P (**5**)

Colorless amorphous white powder; [α]_D_^25^ − 20.00 (c 0.1, MeOH); IR ν_max_ 3520, 2925, 1775, 1765, 1675, 1350, 1220, 1025, 950, 750 cm^−1^; ^1^H-NMR data (500 MHz, CDCl_3_), see Table 1; ^13^C-NMR data (125 MHz, CDCl_3_), see Table 2. HRESIMS *m*/*z* 479.1437 [M + Na]^+^ (calcd. For C_22_H_29_ClO_8_Na, 479.1449).

#### Brianthein X (**6**)

Colorless white powder; [α]_D_^25^ − 49.61 (c 0.3, MeOH); IR ν_max_ 3566, 2963, 1788, 1739, 1363, 1220, 1025, 950, 750 cm^−1^; HRESIMS *m*/*z* 521.1543 [M + Na]^+^ (calcd. For C_24_H_31_ClO_9_Na, 521.1554).

#### Brianthein Y (**7**)

Colorless amorphous white powder; [α]_D_^25^ + 35.46 (c 0.3, MeOH); IR ν_max_ 3575, 2964, 1788, 1739, 1364, 1220, 1025, 925, 750 cm^−1^; HRESIMS *m*/*z* 591.1966 [M + Na]^+^ (calcd. For C_28_H_37_ClO_10_Na, 591.1973).

#### Brianthein Z (**8**)

Colorless amorphous white powder; [α]_D_^25^ + 28.90 (c 0.3, MeOH); IR ν_max_ 3550, 2930, 1790, 1739, 1365, 1220, 1025, 950, 750 cm^−1^; HRESIMS m/z 563.1663 [M + Na]^+^ (calcd. For C_26_H_33_ClO_10_Na, 563.1660).

#### Asbestinin-10 (**9**)

Colorless amorphous white powder; [α]_D_^25^ − 17.63 (c 0.2, MeOH); IR ν_max_ 3550, 2968, 2926, 2872, 1731, 1726, 1687, 1638, 1440, 1385, 1235, 1126, 918, 758 cm-1; HRESIMS *m*/*z* 399.2165 [M + Na]^+^ (calcd. For C_22_H_32_O_5_Na, 399.2147).

#### Lactone 14 (**10**)

Colorless amorphous white powder; [α]_D_^25^ + 42.65 (c 0.1, MeOH); IR ν_max_ 3550, 2968, 2926, 2872, 1731, 1726, 1687, 1638, 1440, 1385, 1340, 1220, 1126, 1025, 900, 775 cm-1; HRESIMS *m*/*z* 515.2240 [M + Na]^+^ (calcd. For C_26_H_36_O_9_Na, 515.2257).

### Vibrational circular dichroism studies

The data were acquired on a BioTools dualPEM Chiral*IR* FT spectrophotometer using a solution of 5.0 mg of **1** in 150 *μ*L of 100% D atom CDCl_3_. The solution was placed in a cell having BaF_2_ windows and a path-length of 100 μm. The data were measured at a resolution of 4 cm^−1^ for 6 h and the base-line was provided by subtracting the spectrum of the solvent acquired under identical experimental conditions. The stability of **1** during the data acquisition procedure was verified by ^1^H NMR spectroscopy immediately before and after the VCD measurement. The in silico constructed molecular models of the four diastereoisomers of **1** at C-2 and C-9 were subjected to Monte Carlo search protocols in a 10 kcal/mol energy window using Merck Molecular Force Field (MMFF94) as implemented in the Spartan’04 program without any conformational restriction. These searches provided 25 conformers for the (2*R*,9*R*) diastereoisomer, 11 for the (2*R*,9*S*) diastereoisomer, 52 for the (2*S*,9*S*) diastereoisomer, and 41 for the (2*S*,9*R*) diastereoisomer in 9.93, 9.06, 9.98, and 9.77 kcal/mol energy windows, respectively. These 129 conformational models were subjected to single point energy calculation using DFT at the B3LYP/6-31G(d) level of theory in the same software suit. These procedures provided five, six, seven, and nine conformers, in the same order respectively, found in the initial 5 kcal/mol. These 28 conformers were subjected to geometry optimization and energy calculation by DFT using the B3LYP/DGDZVP level of theory as implemented in the Gaussian 09 program. The three, five, four, and three minimized structures, in the same order respectively, contributing individually with more than 1% to the total conformational distribution of each diastereoisomer, provided the thermochemical parameters given in Table S2 in Electronic Supporting Information and were used to calculate the IR and VCD frequencies at 298 K and 1 atm. All minimum energy structures were verified for the absence of imaginary frequencies and their relative free energies were employed to calculate their Boltzmann population. The Boltzmann-weighted IR and VCD spectra were calculated considering Lorentzian bands with half-widths of 6 cm^−1^. Molecular visualization was accomplished using the GaussView 6.0 program. Geometry optimization and vibrational calculations required some 65 h of CPU time per conformer when using a PC operated at 3.5 GHz and 4 Gb RAM.

### Single-crystal X-ray diffraction analysis

Suitable colorless crystals of **1** were grown by slow evaporation from methanol. A crystal measuring 0.53 × 0.312 × 0.178 mm was mounted on a diffractometer equipped with graphite monochromated Mo *Kα* (*λ* = 0.71073) radiation. The crystal was orthorhombic, *P2*_1_*2*_1_*2*_1_, C_24_H_31_ClO_8_, *a* = 12.6098(4) Å, *b* = 14.09741(5) Å, *c* = 27.0326(10) Å, *V* = 4805.5(3) Å^3^, *Z* = 4 and *Z’* = 2 . *ρ*_calcd_ = 1.359 g/cm^3^, 0.208 *μ*/mm^−1^, F(000) = 2084, *T* = 100 K. A total of 668,812 reflections were collected, of which 25,782 unique reflections (R_int_ = 0.0453) with I > 2*σ*(I) were used for the analysis. The structure was solved by direct methods and refined by full-matrix least-squares on F2. The non-hydrogen atoms were refined anisotropically and the hydrogen atoms were rided at geometrically idealized positions on their parent atoms. The final indices were R_1_ 3.54%, wR_2_ 9.61%, GOF = 1.093, and Flack parameter x = 0.017. The X-ray structure plot is shown in Fig. 4. Crystallographic data (excluding structure factors) have been deposited (CCDC deposition number 2009802) with the Cambridge Crystallographic Data Centre from where they can be obtained, free of charge, via http://www.ccdc.ac.uk/data_request/cif.

### Cell culture

HaCaT cells were cultured in DMEM supplemented with 10% FBS and 1% penicillin/streptomycin. THP-1 and THP-1 Lucia monocytes (stably transfected with a NF-κB-inducible Luc reporter construc) were maintained in RPMI 1640 medium, containing 10% FBS, 1% penicillin/streptomycin and 25 mM HEPES. In the case of THP-1 Lucia cells Normocin was added to the media at a final concentration of 100 µg/mL and a selective antibiotic, Zeocin, was added every other passage at 100 μg/mL, as recommended by the supplier. All cell lines were incubated at 37 °C, in a humidified atmosphere of 5% CO_2_.

### MTT viability assay

Monocytes were seeded at a density of 6.0 × 10^4^ cells/well. PMA (50 nM) was added as a differentiation agent to obtain macrophages. After 24 h, this medium was discarded and replaced with fresh PMA-free medium for another 24 h period, after which the differentiated M1-macrophages were incubated with the compounds of interest for 24 h. After this period the wells were aspirated, and the medium replaced with MTT at 0.5 mg/mL and incubated for 2 h. At the end of this period, the solution was discarded and the formazan crystals in the well dissolved in 200 µL of a DMSO:isopropanol solution (3:1). The absorbance at 560 nm was read in a Thermo Scientific Multiskan GO microplate reader. For HaCaT cells, the density used was 1.5 × 10^4^ cells/well.

### NF-κB activation assay

THP-1 Lucia NF-κB monocytes were seeded on 96-well plates and differentiated into macrophages as described above for non-transfected THP-1 monocytes and incubated with the selected compounds. After 2 h, LPS from *E. coli* was added to each well at a final concentration of 1 µg/mL. 22 h later, 20 µL of supernatant were collected from each well and transferred to a white 96-well plate. Then, as indicated by the supplier, 50 µL of QUANTI-Luc assay solution was added to each well, the plate was shaken, and luminescence was immediately read in a Cytation 3 (BioTek) microplate reader.

### RNA extraction, quantification, integrity, and conversion

THP-1 cells were seeded at a density of 4.8 × 10^5^ cells/well in 12-well plates and differentiated into macrophages as described above and treated for 22 h with LPS (1 µg/mL), with or without pre-incubation with compounds **6** and **9** for 2 h. Afterwards, the supernatant was removed, and the cells were disrupted in 500 µL of PureZOL reagent. Then, the samples were transferred to a RNase-free tube and 100 µL of chloroform were added. The mixture was shaken vigorously for 15 s. After 5 min incubation at room temperature, the samples were centrifuged at 12,000 × *g* for 15 min at 4 °C. Following centrifugation, the aqueous phase containing the RNA was immediately transferred to a new RNase-free tube and 250 µL of isopropyl alcohol were added, and mixture was incubated at room temperature for 5 min. Afterwards, the tubes were centrifuged at 12,000 × *g* for 10 min at 4 °C, the RNA appearing as a white pellet on the side and bottom of the tube. Supernatant was carefully discarded, and the RNA pellet was washed with 1 mL of 75% of ethanol. After vortexing, the mixture was centrifuged at 7500 × *g* for 5 min at 4 °C and the supernatant was carefully discarded. Then, the RNA pellet was air-dried for about 5 min and reconstituted in 25 µL of PCR grade water. Subsequently, the RNA was quantified in a Qubit 4 fluorometer (Invitrogen by Thermo Fisher Scientific; Waltham, MA, USA), using the Qubit RNA HS assay kit. The RNA quality and integrity were then evaluated using the Qubit RNA IQ assay kit. In order to obtain the complementary DNA, 1 µg of RNA was mixed with 4 µL qScript cDNA SuperMix in a 20-µL reaction. The reverse-transcribed reaction involved three steps: 5 min at 25 °C, 30 min at 42 °C, and 5 min at 85 °C^54^.

### q-PCR analysis

q-PCR analysis were conducted on multiple genes, namely COX-2, TNF-α, IL-6, and IL-1β (Table S9 in ESI). GAPDH was used as reference gene (Table S9 in ESI). The primers were designed using the Primer-BLAST tool (NCBI, Bethesda, MD, USA) and synthesized by Thermo Fisher (Waltham, MA, USA), as listed in Table S9 in the Supplementary Information.

Real-time qPCR was performed with 2 ng of cDNA using KAPA SYBR FAST qPCR Kit Master Mix (2X) Universal similarly to what we have described before^55^. The thermal cycling conditions were as follows: 3 min at 95 °C, followed by 40 cycles of denaturation at 95 °C for 3 s, specific annealing temperatures for each gene (Table S9 In the Supplementary Information) for 20 s, and extension at 72 °C for 20 s. The fluorescence signal was detected at the end of each cycle. The results were analyzed with qPCRsoft 4.0 supplied with the equipment qTOWER3 G (Analytik Jena AG, Germany), and a melting curve was used to confirm the specificity of the products. Transcript abundances of the target genes were normalized to the expression of GAPDH (reference gene). Samples were run in duplicate in each PCR assay. Normalized expression values were calculated following the mathematical model proposed by Pfaffl using the formula: 2 − ΔΔCt^56^ At least three independent experiments were performed.

### Statistical analysis

For biological assays, the Shapiro-Wilks normality test was performed in the data to ensure that it followed a normal distribution. Comparison between the means of controls and each experimental condition was performed using ANOVA. Outliers were identified by the Grubbs’ test. Data was expressed as the mean ± standard error of the mean (SEM) of at least 3 independent experiments, each performed in triplicate. GraphPad Prism software was used, and values were considered statistically significant with a *p* < 0.05. All results represent the mean ± SEM.

## Supplementary Information

Supplementary Information.

## References

[1] Dickschat JS (2019). Bacterial diterpene biosynthesis. Angew. Chem. Int. Ed..

[2] Albano G, Aronica LA (2020). Acyl Snogashira cross-coupling: state of the art and application to the synthesis of heterocyclic compounds. Catalysts.

[3] Rodríguez J, Nieto RM, Jiménez C (1998). New Briarane Stecholide Diterpenes from the Indonesian Gorgonian *Briareum*sp. †. J. Nat. Prod..

[4] Gómez-Reyes JF (2012). Seco-briarellinone and briarellin S, two new eunicellin-based diterpenoids from the panamanian octocoral *Briareum asbestinum*. Mar. Drugs.

[5] Gupta P (2011). Bioactive diterpenoid containing a reversible ‘spring-loaded’ (E, Z)-dieneone Michael acceptor. Org. Lett..

[6] Meginley RJ (2012). Briareolate esters from the Gorgonian *Briareum asbestinum*. Mar. Drugs.

[7] González N, Rodríguez J, Kerr RG, Jiménez C (2002). Cyclobutenbriarein A, the first diterpene with a tricyclo[8.4.0.0.3,6]tetradec-4-ene ring system isolated from the Gorgonian Briareum asbestinum. J. Org. Chem..

[8] Burks JE, van der Helm D, Chang CY, Ciereszko LS (1977). The crystal and molecular structure of briarein A, a diterpenoid from the gorgonian *Briareum abestinum*. Acta Crystallogr Sect. B Struct. Crystallogr. Cryst. Chem..

[9] Sung PJ, Sheu JH, Xu JP (2002). Survey of briarane-type diterpenoids of marine origin. Heterocycles.

[10] Sung PJ (2005). Survey of briarane-related diterpenoids- part II. Heterocycles.

[11] Sung PJ (2008). Survey of briarane-type diterpenoids-part III. Heterocycles.

[12] Sung PJ (2011). Survey of briarane-type diterpenoids - Part IV. Heterocycles.

[13] Sheu JH (2014). Briarane diterpenoids isolated from gorgonian corals between 2011 and 2013. Mar. Drugs.

[14] Liaw C-C (2014). New Briarane Diterpenoids from Taiwanese Soft Coral Briareum violacea. Mar. Drugs.

[15] Jiménez C (2018). Marine natural products in medicinal chemistry. ACS Med. Chem. Lett..

[16] Pech-Puch D, Pérez-Povedano M, Lenis-Rojas OA, Rodríguez J, Jiménez C (2020). Marine natural products from the Yucatan Peninsula. Mar. Drugs.

[17] Pech-Puch D (2020). Antiviral and antiproliferative potential of marine organisms from the Yucatan Peninsula, Mexico. Front. Mar. Sci..

[18] Pech-Puch D (2020). Marine organisms from the Yucatan Peninsula (Mexico) as a potential natural source of antibacterial compounds. Mar. Drugs.

[19] Rodríguez J (1994). Variation among known kalihinol and new kalihinene diterpenes from the sponge *Acanthella cavernosa*. Tetrahedron.

[20] Anta C, González N, Rodríguez J, Jiménez C (2002). A new secosterol from the Indonesian octocoral *Pachyclavularia violacea*. J. Nat. Prod..

[21] Reyes F (2004). New cytotoxic cembranes from the sea pen *Gyrophyllum siboga*e. J. Nat. Prod..

[22] Tello E (2011). Absolute stereochemistry of antifouling cembranoid epimers at C-8 from the Caribbean octocoral *Pseudoplexaura flagellosa* Revised structures of plexaurolones. Tetrahedron.

[23] Pardo-Vargas A (2014). Dolabelladienols A-C, new diterpenes isolated from Brazilian brown alga *Dictyota pfaffii*. Mar. Drugs.

[24] Urda C (2017). Protoxenicins A and B, cytotoxic long-chain acylated xenicanes from the soft coral *Protodendron repens*. J. Nat. Prod..

[25] Pech-Puch D, Rodríguez J, Cautain B, Sandoval-Castro CA, Jiménez C (2019). Cytotoxic Furanoditerpenes from the Sponge *Spongia tubulifera* collected in the Mexican Caribbean. Mar. Drugs.

[26] Cannell RJP (1998). Natural Products Isolation, vol 4.

[27] Pech-Puch D (2020). In vitro and in vivo assessment of the efficacy of bromoageliferin, an alkaloid isolated from the Sponge *Agelas dilatata*, against *Pseudomonas aeruginosa*. Mar. Drugs.

[28] Groweiss A, Look SA, Fenical W (1988). Solenolides, new antiinflammatory and antiviral diterpenoids from a marine octocoral of the genus *Solenopodium*. J. Org. Chem..

[29] Harvell CD (1993). Local and geographic variation in the defensive chemistry of a West Indian gorgonian coral (*Briareum asbestinum*). Mar. Ecol. Prog. Ser..

[30] Pech-Puch, D., Rodríguez, J. & Jiménez, C. I. Euroindoamerican natural products meeting. in *Abstracts Book* 33 (2018).

[31] Su YD (2016). Briarenolides M-T, new briarane diterpenoids from a Formosan octocoral *Briareum* sp. Tetrahedron.

[32] Esquivel B (2018). Absolute configuration of the diterpenoids icetexone and conacytone from *Salvia ballotaeflora*. Chirality.

[33] Joseph-Nathan P, Gordillo-Román B (2015). Progress in the chemistry of organic natural products. Prog. Chem. Org. Nat. Prod..

[34] Díaz-Fernández M, Salazar MI, Joseph-Nathan P, Burgueño-Tapia E (2019). Configurational Study of Diastereoisomeric Royleanone Diterpenoids from *Salvia concolor*. Nat. Prod. Commun..

[35] Gutiérrez-Nicolás F (2012). Synthesis and anti-HIV activity of lupane and olean-18-ene derivatives. Absolute configuration of 19,20-epoxylupanes by VCD. J. Nat. Prod..

[36] Holtje H, Folkers G (1997). Molecular Modeling: Basic Principles and Applications. Methods and Principles in Medicinal Chemistry.

[37] Debie E (2011). A confidence level algorithm for the determination of absolute configuration using vibrational circular dichroism or raman optical activity. ChemPhysChem.

[38] Flack HD, Bernardinelli G (2008). The use of X-ray crystallography to determine absolute configuration. Chirality.

[39] Hooft RWW, Straver LH, Spek AL (2008). Determination of absolute structure using Bayesian statistics on Bijvoet differences. J. Appl. Crystallogr..

[40] Dolomanov OV, Bourhis LJ, Gildea RJ, Howard JAK, Puschmann H (2009). OLEX2: a complete structure solution, refinement and analysis program. J. Appl. Crystallogr..

[41] Nath N (2020). Relative configuration of micrograms of natural compounds using proton residual chemical shift anisotropy. Nat. Commun..

[42] Liu Y (2019). Application of anisotropic NMR parameters to the confirmation of molecular structure. Nat. Protoc..

[43] Poza JJ, Jiménez C, Rodríguez J (2008). J-based analysis and DFT-NMR assignments of natural complex molecules: application to 3β,7-dihydroxy-5,6-epoxycholestanes. Eur. J. Org. Chem..

[44] Grode SH, James TR, Cardellina JH, Onan KD (1983). Molecular structures of the briantheins, new insecticidal diterpenes from briareum polyanthes. J. Org. Chem..

[45] Van der Helm D, Loghry RA, Matson JA, Weinheimer AJ (1986). Crystal and molecular structure of brianthein X. J. Crystallogr. Spectrosc. Res..

[46] Linz GS, Weinheimer AJ, Martin GE, Musmar MJ, Matson JA (1986). Two-dimensional NMR studies of marine natural products. III. Reassignment of the 13C-NMR spectrum of brianthein-X using heteronuclear relayed coherence transfer. Spectrosc. Lett..

[47] Grode SH, James TR, Cardellina JH (1983). Brianthein Z, a new polyfunctional diterpene from the Gorgonian *Briareum polyanthes*. Tetrahedron Lett..

[48] Rodríguez AD, Cóbar OM (1993). Structures and bioactivities of new asbestinin diterpenoids from the caribbean gorgonian octocoral *Briareum asbestinum*. Tetrahedron.

[49] Ospina CA, Rodríguez AD (2006). Bioactive compounds from the gorgonian Briareum polyanthes. Correction of the structures of four asbestinane-type diterpenes. J. Nat. Prod..

[50] Wratten SJ, Faulkner DJ (1979). Some diterpenes from the sea pen *Stylatula* sp. Tetrahedron.

[51] Sung P-J (2020). Survey of briarane-type diterpenoids—part VII. Heterocycles.

[52] Sullivan B, Gilmet J, Leisch H, Hudlicky T (2008). Chiral version of the burgess reagent and its reactions with oxiranes: application to the formal enantiodivergent synthesis of balanol. J. Nat. Prod..

[53] Ferchimin, P. A., Eterovic, de F. V. A. & Maldonado-Maldonado, H. M. Neuronal circuit-dependent neuroprotection by interaction between nicotinic receptors. WO/2008/002594, 67pp (2008).

[54] Pereira RB (2019). Anti-inflammatory effects of 5α,8α-epidioxycholest-6-en-3β-ol, a steroidal endoperoxide isolated from *Aplysia depilans*, based on bioguided fractionation and NMR analysis. Mar. Drugs.

[55] Ribeiro, V., Andrade, P. B., Valentão, P. & Pereira, D. M. Benzoquinones from Cyperus spp. trigger IRE1α-independent and PERK-dependent ER stress in human stomach cancer cells and are novel proteasome inhibitors. *Phytomedicine* **63**, 153017 (2019). 10.1016/j.phymed.2019.153017 31325684

[56] Pfaffl MW (2001). A new mathematical model for relative quantification in real-time RT-PCR. Nucleic Acids Res..

