# Supplementary material for: Absolute configuration by vibrational circular dichroism of anti-inflammatory macrolide briarane diterpenoids from the Gorgonian Briareum asbestinum
Source: Sci Rep. 2021 Jan 12;11:496. doi: 10.1038/s41598-020-79774-1 (PMC7804146; doi:10.1038/s41598-020-79774-1)
Supplement: Supplementary file 1 — Supplementary Information. [file 41598_2020_79774_MOESM1_ESM.pdf]

Scientific Reports

Supplementary Information

Absolute Configuration by Vibrational Circular Dichroism of Anti-inflammatory Macrolide Briarane Diterpenoids from the Gorgonian *Briareum asbestinum*

Dawrin Pech-Puch,[1,4] Pedro Joseph-Nathan,[2] Eleuterio Burgueño-Tapia,[3] Carlos González-Salas,[4] Diana Martínez-Matamoros,[1] David M. Pereira,[5] Renato B. Pereira,[5] Carlos Jiménez,[*,1] Jaime Rodríguez*[,1]

[1]Centro de Investigacións Científicas Avanzadas (CICA) e Departamento de Química, Facultade de Ciencias, Agrupación Estratéxica CICA-INIBIC, Universidade da Coruña, A Coruña 15071, Spain*
[2]Departamento de Química, Centro de Investigación y de Estudios Avanzados del Instituto Politécnico Nacional, Apartado 14-740, Mexico City 07000, Mexico
[3]Departamento de Química Orgánica, Escuela Nacional de Ciencias Biológicas, Instituto Politécnico Nacional, Prolongación de Carpio y Plan de Ayala, Col. Santo Tomás, Mexico City 11340, Mexico
[3]Departamento de Química Orgánica, Escuela Nacional de Ciencias Biológicas, Instituto Politécnico Nacional, Prolongación de Carpio y Plan de Ayala, Col. Santo Tomás, Mexico City 11340, Mexico
[4]Departamento de Biología Marina, Universidad Autónoma de Yucatán, Km. 15.5, carretera Mérida-Xmatkuil, A.P. 4-116 Itzimná, C.P. 97100, Mérida, Yucatán, México
[5]REQUIMTE/LAQV, Laboratório de Farmacognosia, Departamento de Química, Faculdade de Farmácia, Universidade do Porto, R. Jorge Viterbo Ferreira 228, Porto 4050-313, Portugal

Index

Figures

Figure S1. ^{1}H NMR spectrum of briarane B-3 (1) (500 MHz, $CDCl_3$) S5
Figure S2. ^{13}C NMR and DEPT-135 spectrum of briarane B-3 (1) (500 MHz, $CDCl_3$) S5
Figure S3. Edited -HSQC spectrum of briarane B-3 (1) (500 MHz, $CDCl_3$) S6
Figure S4. COSY spectrum of briarane B-3 (1) (500 MHz, $CDCl_3$) S6
Figure S5. HMBC spectrum of briarane B-3 (1) (500 MHz, $CDCl_3$) S7
Figure S6. NOESY spectrum of briarane B-3 (1) (500 MHz, $CDCl_3$) S7
Figure S7. (+)-LRESIMS of briarane B-3 (1) S8
Figure S8. (+)-HRESIMS of briarane B-3 (1) S8
Figure S9. ^{1}H NMR spectrum of 2-butyryloxybriarane B-3 (2) (500 MHz, $CDCl_3$) S12
Figure S10. ^{13}C NMR and DEPT-135 spectrum of 2-butyryloxybriarane B-3 (2) (500 MHz, $CDCl_3$) S12
Figure S11. HSQC spectrum of 2-butyryloxybriarane B-3 (2) (500 MHz, $CDCl_3$) S13
Figure S12. COSY spectrum of 2-butyryloxybriarane B-3 (2) (500 MHz, $CDCl_3$) S13
Figure S13. HMBC spectrum of 2-butyryloxybriarane B-3 (2) (500 MHz, $CDCl_3$) S14
Figure S14. NOESY spectrum of 2-butyratebriarane B-3 (2) (500 MHz, $CDCl_3$) S14
Figure S15. (+)-LRESIMS of 2-butyryloxybriarane B-3 (2) S15
Figure S16. (+)-HRESIMS of 2-butyryloxybriarane B-3 (2) S15
Figure S17. ^{1}H NMR spectrum of 9-acetylbriarenolide S (3) (500 MHz, $CDCl_3$) S17
Figure S18. ^{13}C NMR and DEPT-135 spectrum of briarane 5 (3) (500 MHz, $CDCl_3$) S17
Figure S19. Edited-HSQC spectrum of briarane 5 (3) (500 MHz, $CDCl_3$) S18
Figure S20. COSY spectrum of 9-acetylbriarenolide S (3) (500 MHz, $CDCl_3$) S18
Figure S21. HMBC spectrum of 9-acetylbriarenolide S (3) (500 MHz, $CDCl_3$) S19
Figure S22. NOESY spectrum of 9-acetylbriarenolide S (3) (500 MHz, $CDCl_3$) S19
Figure S23. (+)-LRESIMS of 9-acetylbriarenolide S (3) S20
Figure S24. (+)-HRESIMS of 9-acetylbriarenolide S (3) S20
Figure S25. ^{1}H NMR spectrum of briarenolide W (4) (500 MHz, $CDCl_3$) S22
Figure S26. ^{13}C NMR and DEPT-135 spectrum of briarenolide W (4) (500 MHz, $CDCl_3$) S22
Figure S27. Edited-HSQC spectrum of briarenolide W (4) (500 MHz, $CDCl_3$) S23
Figure S28. COSY spectrum of briarenolide W (4) (500 MHz, $CDCl_3$) S23
Figure S29. HMBC spectrum of briarenolide W (4) (500 MHz, $CDCl_3$) S24
Figure S30. NOESY spectrum of briarenolide W (4) (500 MHz, $CDCl_3$) S24
Figure S31. (+)-LRESIMS of briarenolide W (4) S25
Figure S32. (+)-HRESIMS of briarenolide W (4) S25
Figure S33. ^{1}H NMR spectrum isobriarenolide P (5) (500 MHz, $CDCl_3$) S27
Figure S34. ^{13}C NMR and DEPT-135 spectrum of isobriarenolide P (5) (500 MHz, $CDCl_3$) S27
Figure S35. HSQC spectrum of isobriarenolide P (5) (500 MHz, $CDCl_3$) S28
Figure S36. COSY spectrum of isobriarenolide P (5) (500 MHz, $CDCl_3$) S28
Figure S37. HMBC spectrum of isobriarenolide P (5) (500 MHz, $CDCl_3$) S29
Figure S38. NOESY spectrum of isobriarenolide P (5) (500 MHz, $CDCl_3$) S29
Figure S39. (+)-LRESIMS of isobriarenolide P (5) S30
Figure S40. (+)-HRESIMS of isobriarenolide P (5) S30
Figure S41. ^{1}H NMR spectrum of lactone 14 (10) (500 MHz, $CDCl_3$) S32
Figure S42. ^{13}C NMR and DEPT-135 spectrum of lactone 14 (10) (500 MHz, $CDCl_3$) S32
Figure S43. HSQC spectrum of lactone 14 (10) (500 MHz, $CDCl_3$) S33
Figure S44. COSY spectrum of lactone 14 (10) (500 MHz, $CDCl_3$) S33
Figure S45. HMBC spectrum of lactone 14 (10) (500 MHz, $CDCl_3$) S34
Figure S46. (+)-LRESIMS of lactone 14 (10) S34

Figure S47. (+)-HRESIMS of lactone 14 (10) S35
Figure S48. Viability of HaCaT and THP-1 cells exposed to compounds 1, 4, 6-10 at 100 μM for 24 hours, as assessed by the MTT assay S35
Figure S49. The most stable conformers of the (1S,2R,6S,7R,8R,9R,10S,11R,17R) diastereoisomer of briarane B-3. S36
Figure S50. Comparison of the experimental IR (b) and VCD (d) spectra of briarane B-3 (1) with the DFT B3LYP/DGDZVP calculated IR (a) and VCD (c) spectra of its (1S,2R,6S,7R,8R,9R,10S,11R,17R) diastereoisomer. S37
Figure S51. The most stable conformers of the (1S,2R,6S,7R,8R,9S,10S,11R,17R) diastereoisomer of briarane B-3. S38
Figure S52. Comparison of the experimental IR (b) and VCD (d) spectra of briarane B-3 (1) with the DFT B3LYP/DGDZVP calculated IR (a) and VCD (c) spectra of its (1S,2R,6S,7R,8R,9S,10S,11R,17R) diastereoisomer. S39
Figure S53. The most stable conformers of the (1S,2S,6S,7R,8R,9R,10S,11R,17R) diastereoisomer of briarane B-3. S40
Figure S54. Comparison of the experimental IR (b) and VCD (d) spectra of briarane B-3 (1) with the DFT B3LYP/DGDZVP calculated IR (a) and VCD (c) spectra of its (1S,2S,6S,7R,8R,9R,10S,11R,17R) diastereoisomer. S41
Figure S55. Key NOESY correlations observed in 2-butyryloxybriarane B-3 (2). S42
Figure S56. Key NOESY correlations observed in as 9-acetylbriarenolide S (3). S42
Figure S57. Key NOESY correlations observed in briarenolide W (4). S43

Tables

Table S1. NMR data of briarane B-3 (1) in CDCl3 (500 MHz) S4
Table S2. Thermochemical data of the (1S,2ξ,6S,7R,8R,9ξ,10S,11R,17R) diastereoisomers of briarane B-3. S9
Table S3. Confidence level data for the IR and VCD spectra comparison of C-2 and C-9 diastereoisomers of briarane B-3 (1). S10
Table S4. NMR data of 2-butyryloxybriarane B-3 (2) in $CDCl_3$ (500 MHz) S11
Table S5. NMR data of 9-acetylbriarenolide S (3) in $CDCl_3$ (500 MHz) S16
Table S6. NMR data of briarenolide W (4) in $CDCl_3$ (500 MHz) S21
Table S7. NMR data of 12-isobriarenolide P (5) in $CDCl_3$ (500 MHz) S26
Table S8. NMR data of lactone 14 (10) in $CDCl_3$ (500 MHz) S31
Table S9. qPCR target information. S43

Table S1. NMR data of briarane B-3 (**1**) in $CDCl_3$ (500 MHz)

no.	δ_C type	δ_H, mult. (J in Hz)	COSY	HMBC	NOESY
1	44.9, qC			2, 10,13, 14, 15	
2	79.3, CH	4.81, d (9.4)	3	14, 15, 22	3, 10, 14, 15
3	28.5, CH_2	2.41, dt (15.6, 9.4, 9.4)- 1.87, dd (15.6, 10.9)	2, 4		2, 3, 10, 16
4	28.5, CH_2	2.61, dd (15.0, 10.9)- 1.62, m	3	16	4, 6, 7, 9, 16
5	142.6, qC			3, 6, 16	
6	67.3, CH	4.83, bs	7	16	4, 7
7	78.2, CH	5.77, bs	6	6, 9, OH	4, 6, 10, 17, 22
8	82.9, qC			6, 9, 10, 17, 18, OH	
9	74.4, CH	5.11, d (5.1)	10	10, 17, 21, OH	4, 10, 17, 20
10	38.5, CH	2.99, dd (4.7, 5.1)	9, 11	2, 14, 20	2, 3, 7, 9, 11, 16, OH
11	46.8, CH	2.61, dq (4.7, 7.4)	10, 20	9, 20	10, 20
12	202.7, qC			14, 20	
13	126.1, CH	5.97, d (10.4)	14	14	
14	154.7, CH	6.19, d (10.4)	13	2, 13, 15	2, 15
15	19.0, CH	1.16, s		14	2, 14, 20, 21
16	119.9, CH_2	5.69, bs-5.37, s		6	3, 4, 10, 16, OH
17	42.3, CH	2.41, q (7.7)	18	9	7, 9, 18,
18	10.1, CH	1.30, d (7.7)	17	17	17
19	176.4, qC			17, 18	
20	15.1, CH	1.30, d (7.4)	11	10	9, 11, 15
21 Ac	170.3, qC			9	
22 Ac	169.6, qC			2	
CH_3 21	21.9, CH_3	2.14, s		21	15
CH_3 22	21.2, CH_3	2.24, s		22	7
OH		3.43, bs			10, 16

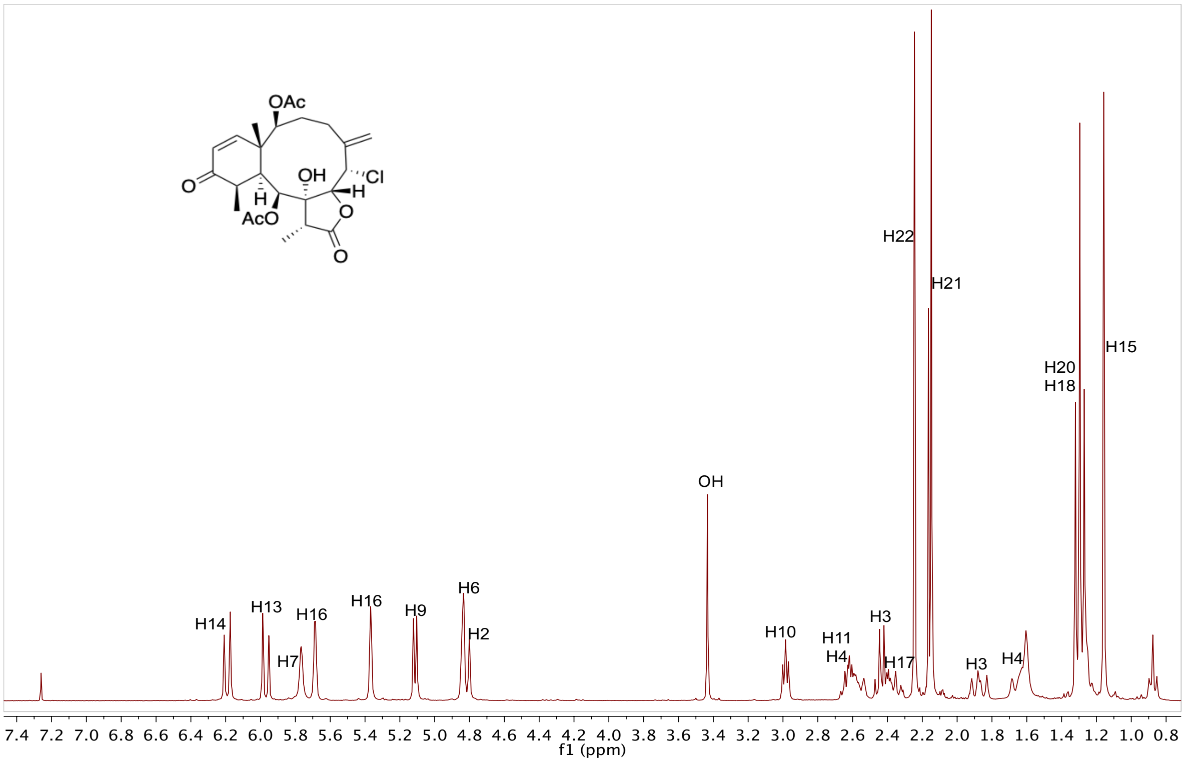

Figure S1. ^{1}H NMR spectrum of briarane B-3 (**1**) (500 MHz, $CDCl_3$)

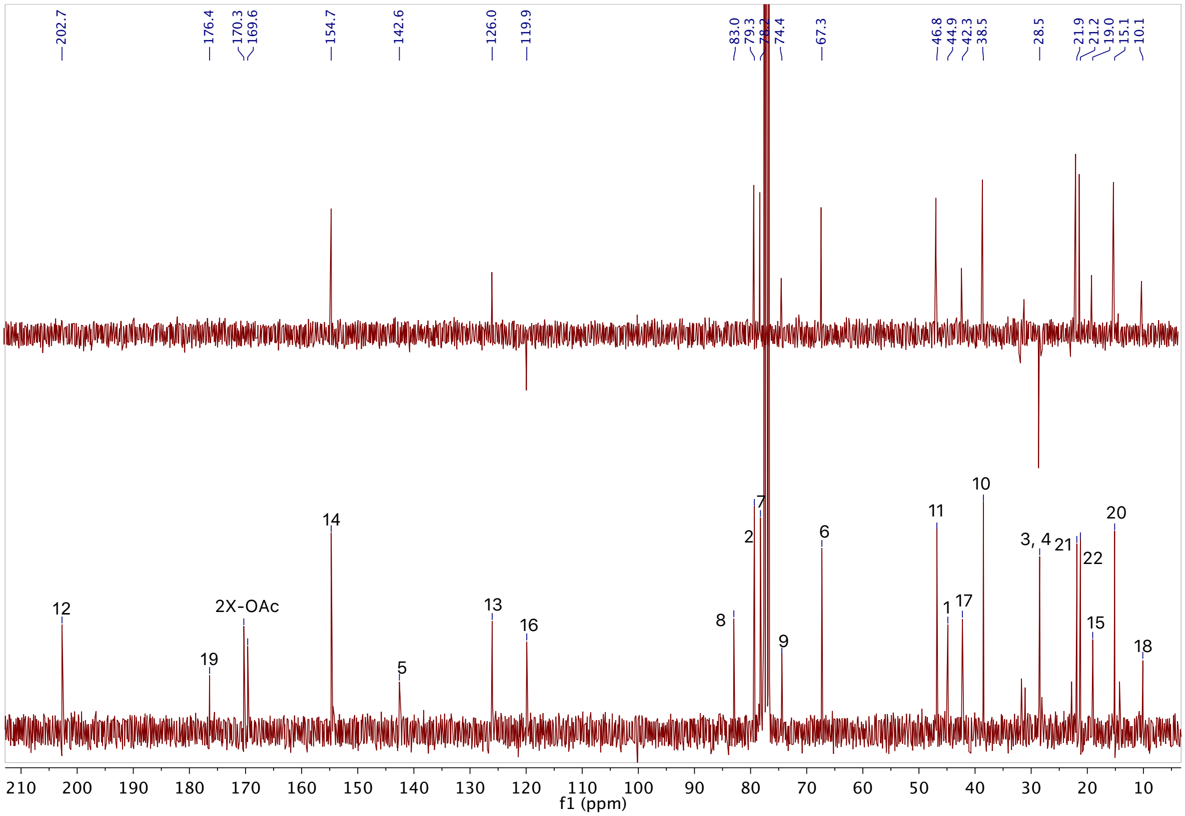

Figure S2. ^{13}C NMR and DEPT-135 spectrum of briarane B-3 (**1**) (500 MHz, $CDCl_3$)

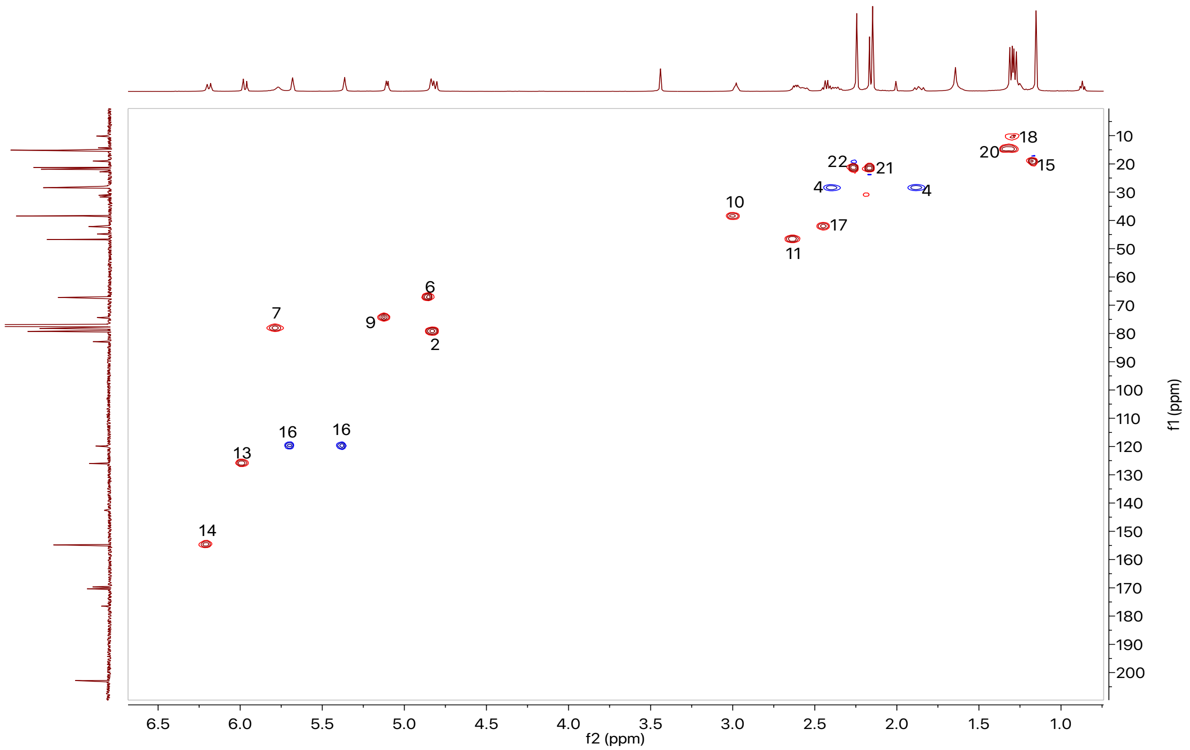

Figure S3. Edited -HSQC spectrum of briarane B-3 (**1**) (500 MHz, $CDCl_3$). CH_2: blue cross-peaks and CH or CH_3: red cross-peaks.

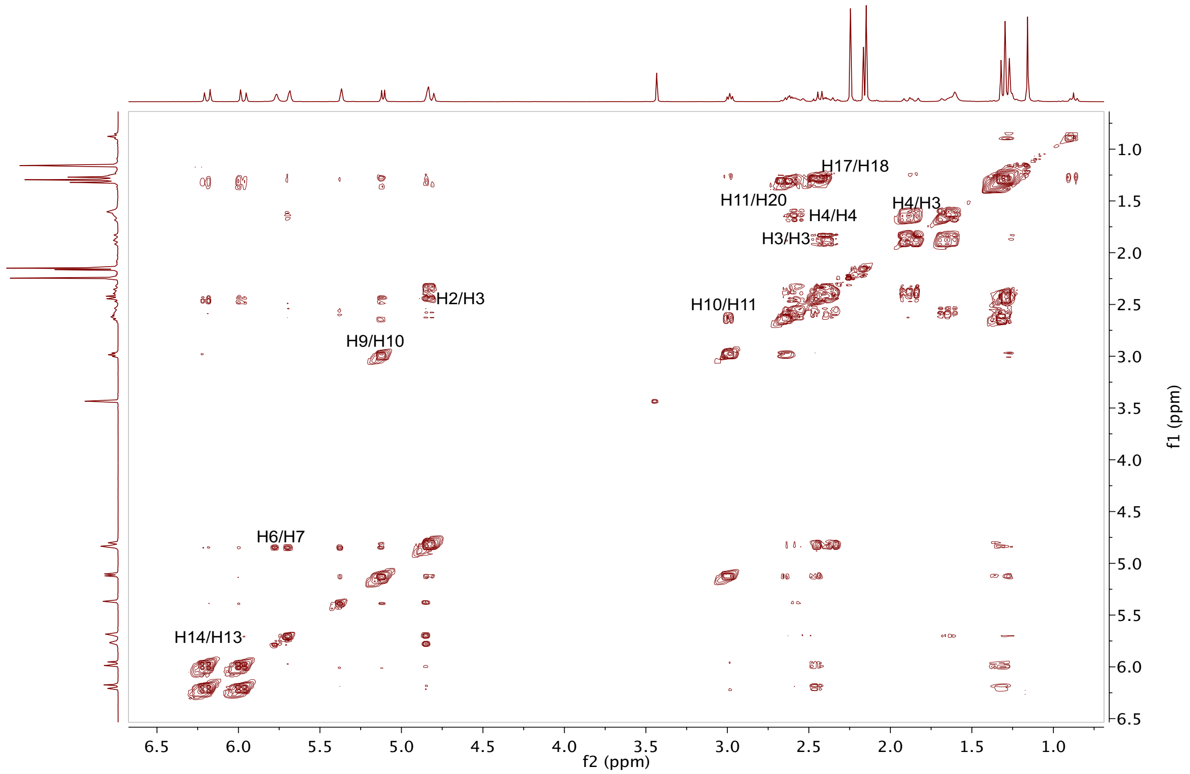

Figure S4. COSY spectrum of briarane B-3 (**1**) (500 MHz, $CDCl_3$)

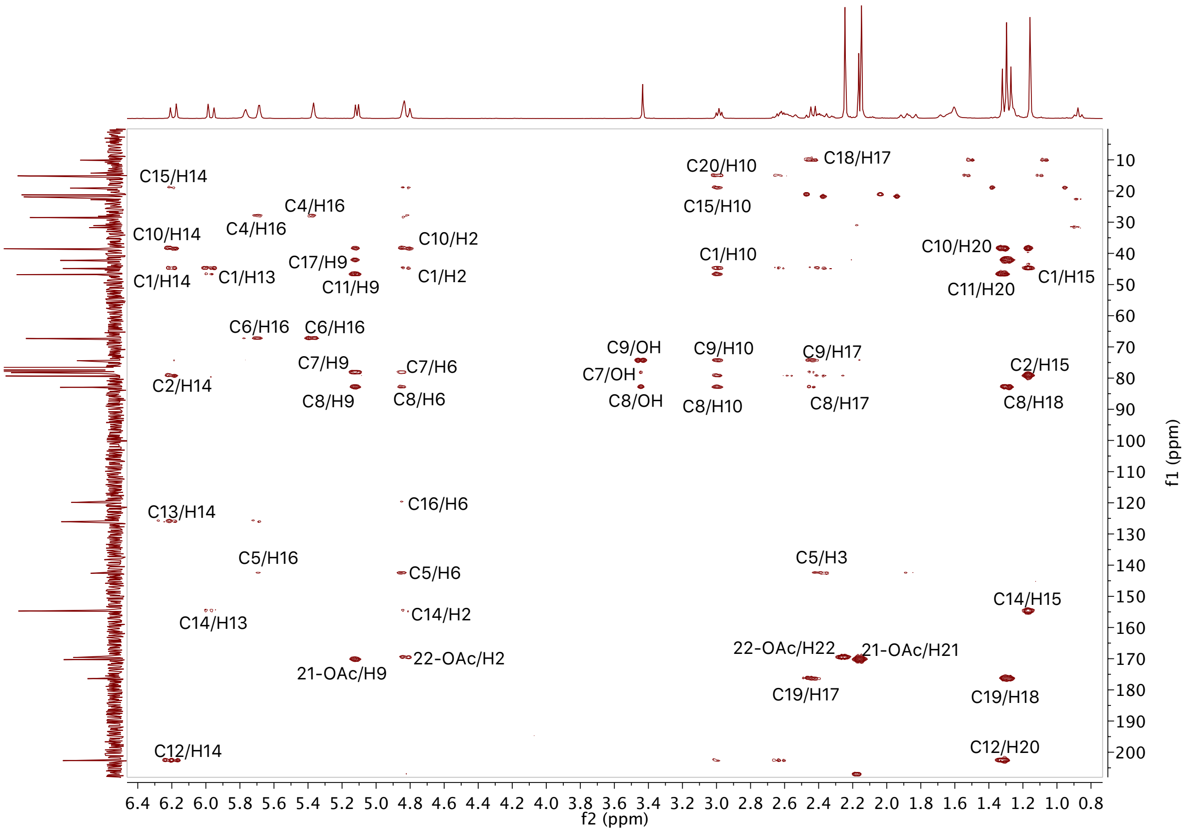

Figure S5. HMBC spectrum of briarane B-3 (**1**) (500 MHz, $CDCl_3$)

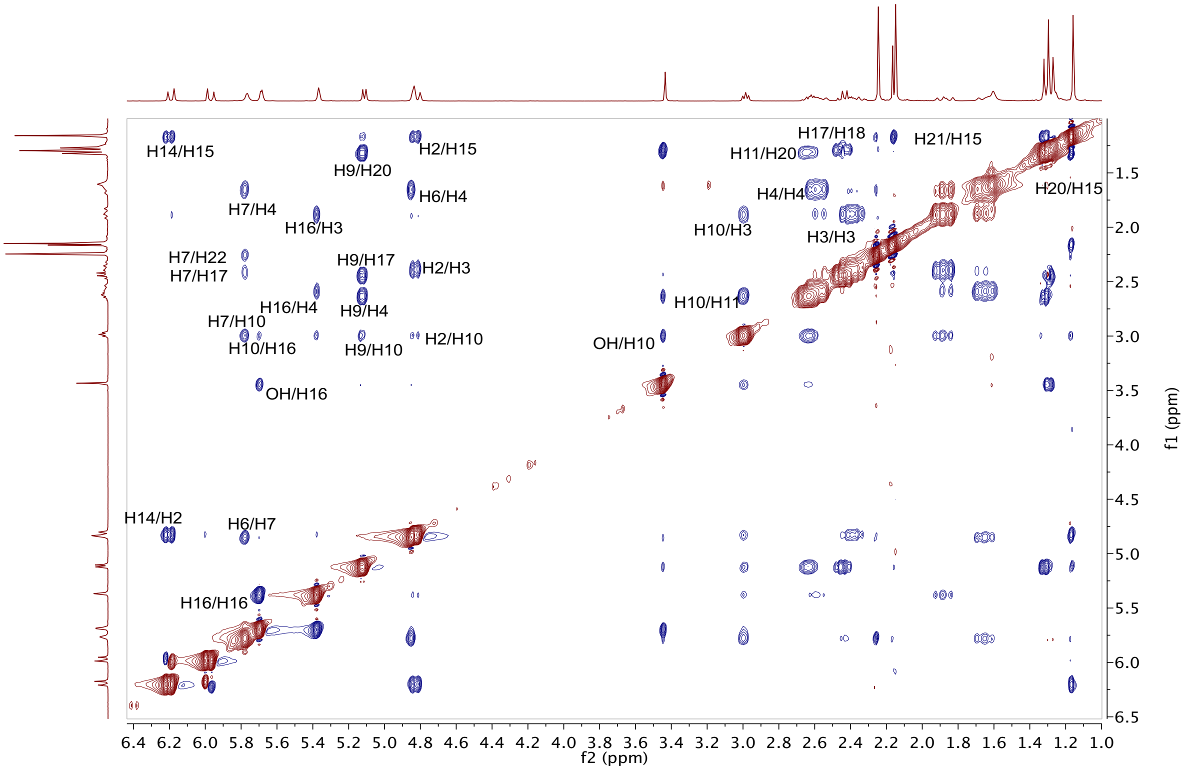

Figure S6. NOESY spectrum of briarane B-3 (**1**) (500 MHz, $CDCl_3$)

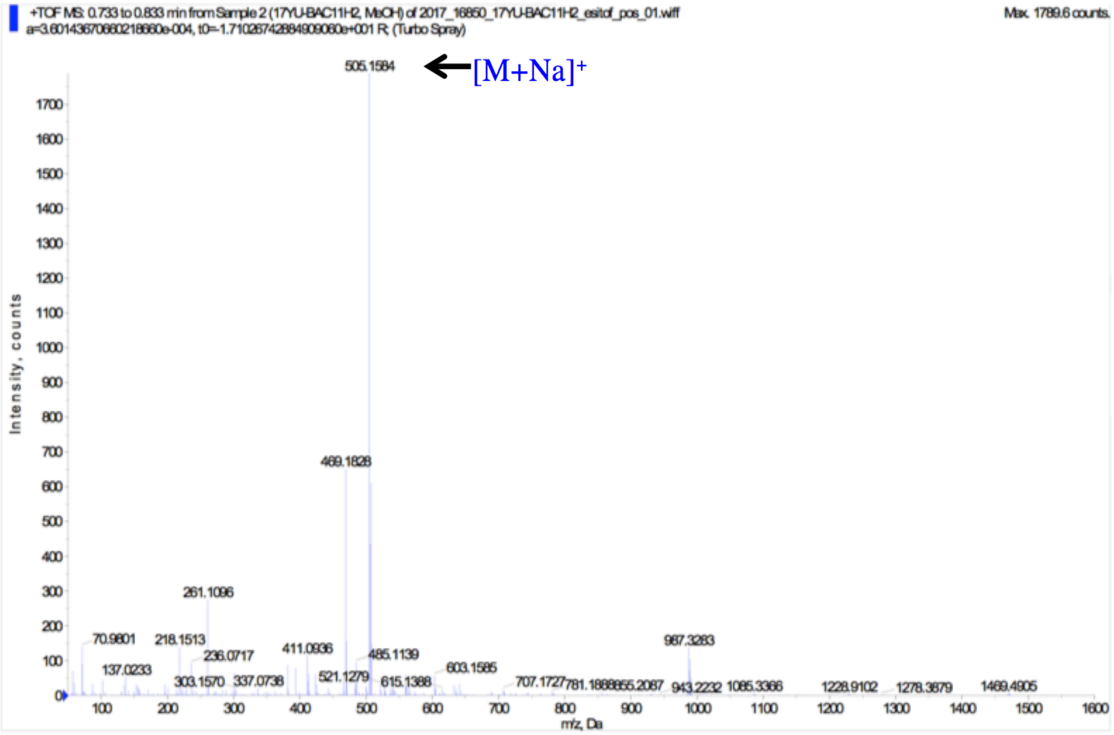

Figure S7. (+)-LRESIMS of briarane B-3 **(1)**

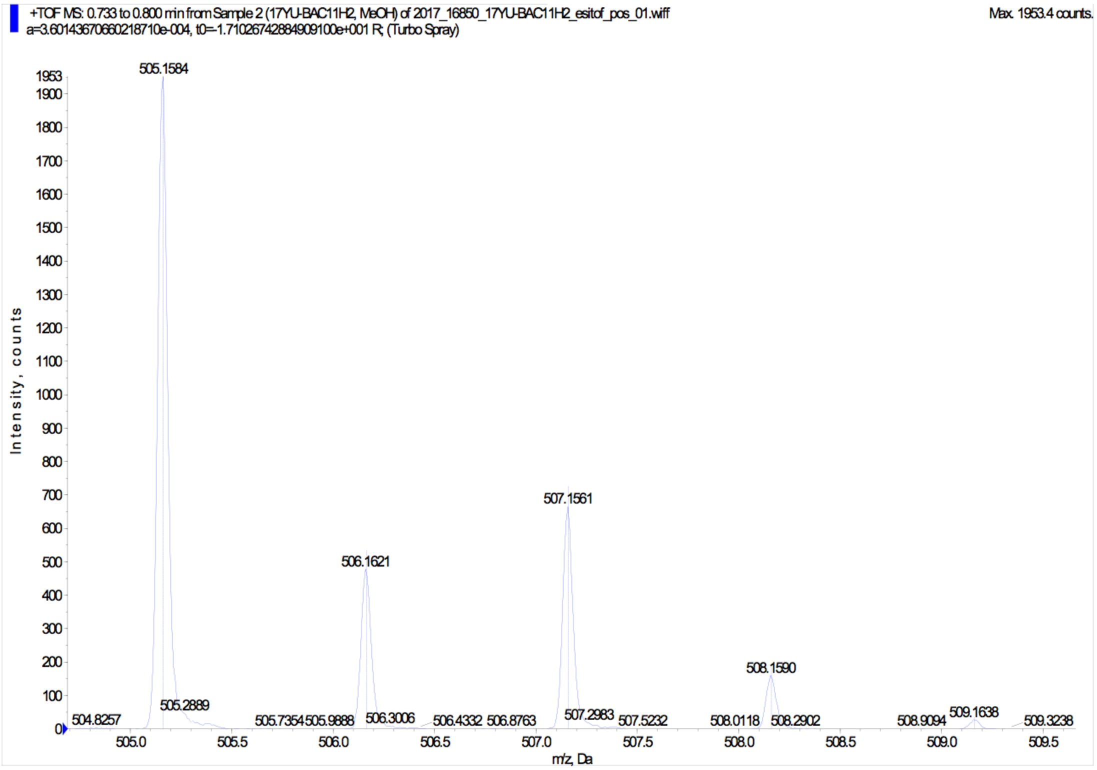

Figure S8. (+)-HRESIMS of briarane B-3 **(1)**

Table S2. Thermochemical data of the (1*S*,2*ξ*,6*S*,7*R*,8*R*,9*ξ*,10*S*,11*R*,17*R*) diastereoisomers of briarane B-3.

Conformer	ΔE_{MMFF}[a]	%[b]	$\Delta E_{\mathrm{6\text{-}31G(d)}}$[c]	%[b]	$\Delta E_{\mathrm{DGDZVP}}$[d]	%[b]	$\Delta G_{\mathrm{DGDZVP}}$[e]	%[f]
**(2*R*,9*R*)-I**	0.00	98.4	0.00	97.3	0.00	97.6	0.00	92.8
**(2*R*,9*R*)-II**	3.04	0.6	2.57	0.4	2.63	1.1	1.73	5.1
**(2*R*,9*R*)-III**	3.51	0.3	2.29	0.2	2.56	1.3	2.23	2.1
**(2*R*,9*S*)-I**	2.91	0.7	0.00	77.5	0.02	29.3	0.00	40.5
**(2*R*,9*S*)-II**	7.02	0.0	2.49	1.1	0.01	29.7	0.29	24.7
**(2*R*,9*S*)-III**	0.00	94.9	1.03	13.7	0.69	9.4	0.48	18.2
**(2*R*,9*S*)-IV**	6.75	0.0	2.46	1.2	0.00	30.2	0.53	16.6
**(2*S*,9*S*)-I**	1.90	2.9	0.00	50.7	0.61	15.2	0.00	38.7
**(2*S*,9*S*)-II**	3.62	0.2	2.21	1.2	0.00	42.4	0.04	36.0
**(2*S*,9*S*)-III**	0.00	79.8	0.04	47.6	0.00	42.2	0.31	23.0
**(2*S*,9*S*)-IV**	0.62	24.7	3.32	0.2	3.66	0.1	1.68	2.3
**(2*S*,9*R*)-I**	0.00	67.3	1.86	3.3	0.08	36.4	0.00	69.5
**(2*S*,9*R*)-II**	1.23	7.9	0.00	75.6	0.00	41.5	0.69	21.6
**(2*S*,9*R*)-III**	0.50	27.4	0.79	20.1	0.40	21.3	1.22	8.9

[a]Relative to **(2*R*,9*R*)-I**: 106.65 kcal/mol, **(2*R*,9*S*)-III**: 107.77 kcal/mol, **(2*S*,9*S*)-III**: 116.29 kcal/mol, **(2*S*,9*R*)-I**: 111.05 kcal/mol. [b]Calculated using $\Delta E \cong -RT \ln K$. [c]Relative to **(2*R*,9*R*)-I**: –1251991.0 kcal/mol, **(2*R*,9*S*)-I**: –1251988.49 kcal/mol, **(2*S*,9*S*)-I**: –1251983.08 kcal/mol, **(2*S*,9*R*)-II**: –1251985.24 kcal/mol. [d]Relative to **(2*R*,9*R*)-I**: –1252099.86 kcal/mol, **(2*R*,9*S*)-IV**: –1252098.55 kcal/mol, **(2*S*,9*S*)-II**: –1252093.65 kcal/mol, **(2*S*,9*R*)-II**: –1252096.781 kcal/mol. [e]Relative to **(2*R*,9*R*)-I**: –1251809.93, **(2*R*,9*S*)-I**: –1251809.05, **(2*S*,9*S*)-I**: –1251804.23, **(2*S*,9*R*)-I**: –1251807.40. [f]Calculated using $\Delta G = -RT \ln K$.

Table S3. Confidence level data for the IR and VCD spectra comparison of C-2 and C-9 diastereoisomers of briarane B-3 (**1**).

Diastereoisomer	*anH*[a]	S_{IR}[b]	S_E[c]	S_{-E}[d]	*ESI*[e]	*C*[f]
(**2*R*,9*R***)	0.972	89.3	24.7	45.3	–20.6	51
(**2*R*,9*S***)	0.976	93.3	48.9	25.6	23.3	62
(**2*S*,9*S***)	0.984	97.3	78.0	18.1	59.9	100
(**2*S*,9*R***)	0.980	94.5	28.1	51.9	–23.8	65

[a]Anharmonicity factor.

[b]IR spectral similarity in percentage.

[c]VCD spectral similarity for the correct enantiomer in percentage.

[d]VCD spectral similarity for the opposite enantiomer in percentage.

[e]Enantiomer similarity index, calculated as the $S_E - S_{-E}$ difference.

[f]Confidence level for the absolute configuration determination in percentage.

Table S4. NMR data of 2-butyryloxybriarane B-3 (**2**) in $CDCl_3$ (500 MHz)

no.	δ_C type	δ_H, mult. (*J* in Hz)	COSY	HMBC	NOESY
1	44.8, qC			13, 15	
2	79.2 CH	4.82, d (9.5)	3	15	3, 10, 14, 15
3	28.6, CH_2	2.40, m-1.86 m	2, 4		2, 3, 10, 16
4	31.7, CH_2	2.57, m-1.60 m	3		4, 6, 7, 16
5	142.6, qC			3, 6, 16	
6	67.3, CH	4.84, d (9.3)	7	16	4, 7, 17
7	78.3, CH	5.71, bs	6	6, 9	4, 6, 10, 17
8	83.0, qC			6, 18	
9	73.5, CH	5.11, d (5.5)	10	17, OH	10, 17, 20
10	38.5, CH	2.99, dd (5.0, 5.5)	9, 11	2, 14, 15, 20	2, 3, 7, 9,11, 16, OH
11	46.9, CH	2.61, m	10, 20	9, 20	10, 20
12	202.8, qC			14, 20	
13	126.0, CH	5.97, d (10.5)	14	14	20
14	154.9, CH	6.20, d (10.5)	13	2, 15	2, 15
15	19.0, CH	1.16, s			2, 2′, 14, 21
16	120.0, CH_2	5.69, q (2.0)-5.37 (bs)		6	3, 4, 10, 16, OH
17	42.3, CH	2.45, q (7.3)	18	9, 18	6, 7, 9, 18, 21
18	14.0, CH	1.30, d (7.3)	17	17	17
19	175.9, qC			17, 18	
20	15.2, CH	1.33, d (7.4)	11		9, 11, 13
21 Ac	170.4, qC			9	
22 Ac	172.6, qC			2	
CH_3 21	21.9, CH_3	2.15, s		21	15, 17
1′CH_2	36.3, CH_2	2.45, q (7.4)	2′	22	2′, 3′
2′CH_2	18.5, CH_2	1.77, m	1′, 3′		1′, 3′15
3′CH_3	14.3 CH_3	1.02, t (7.4)	2′		1′, 2′
OH		3.42, bs			10, 16

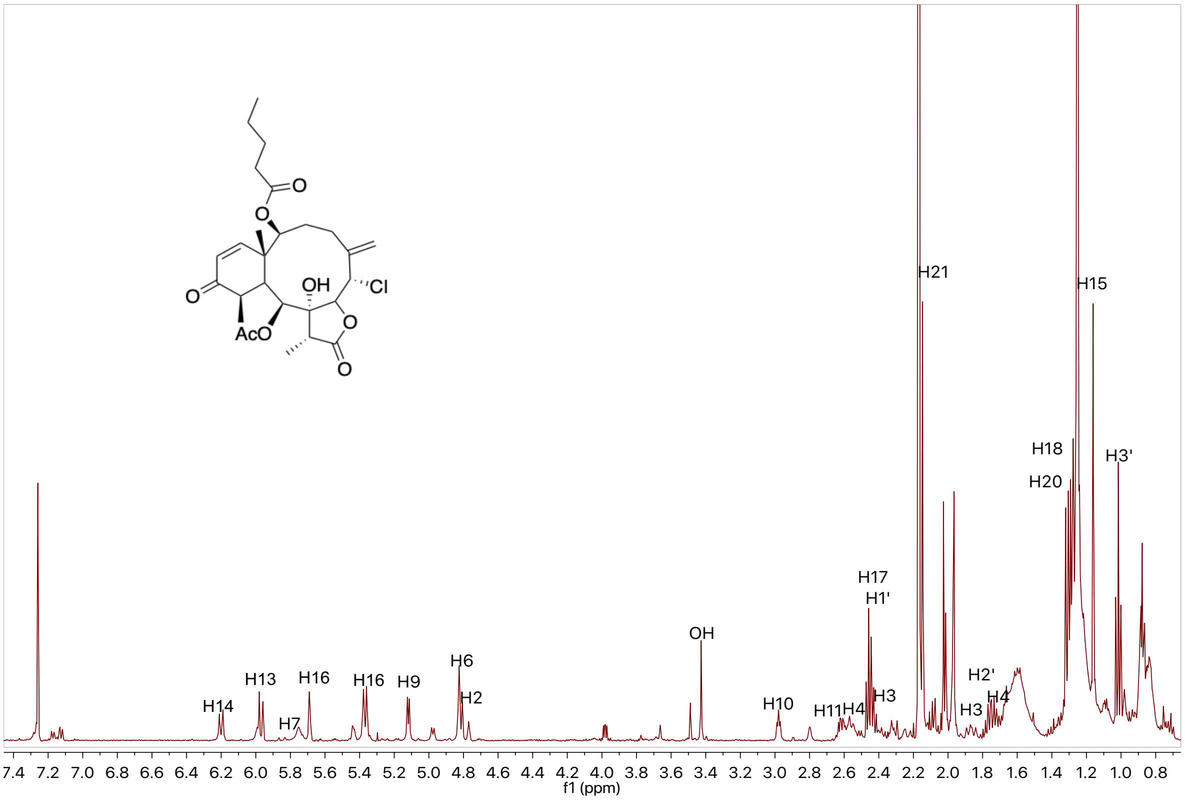

Figure S9. ^{1}H NMR spectrum of 2-butyryloxybriarane B-3 (**2**) (500 MHz, $CDCl_3$)

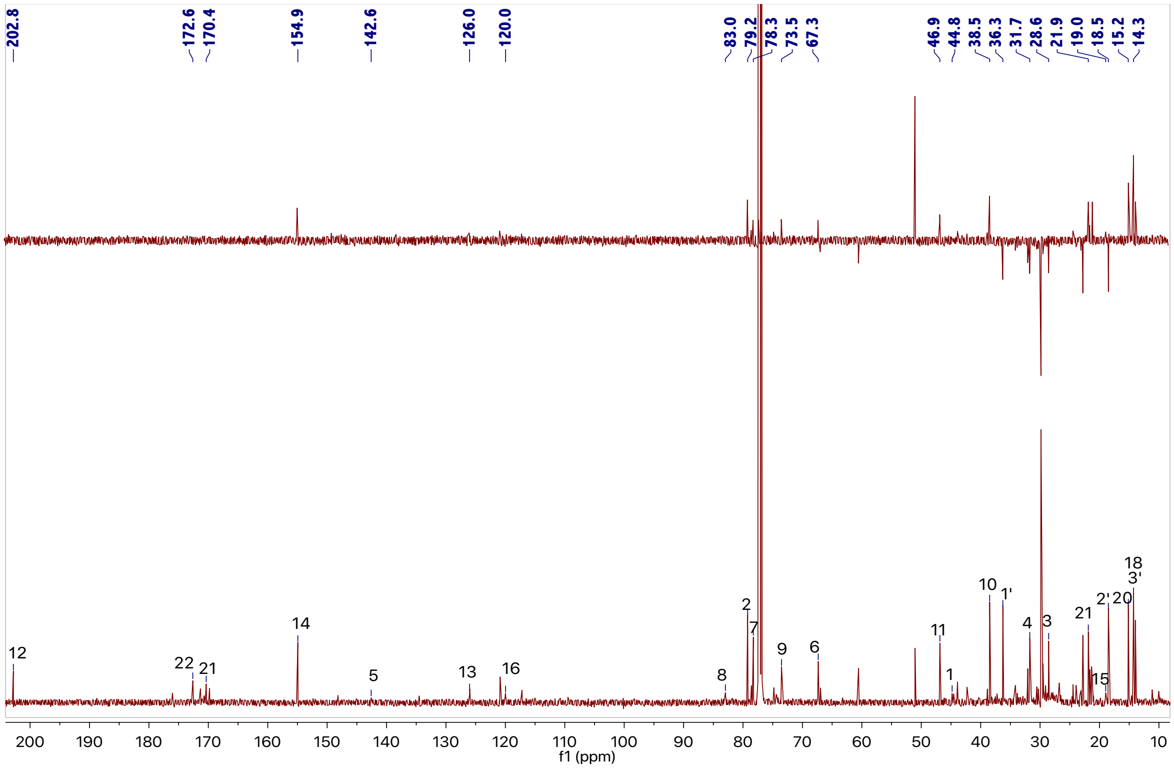

Figure S10. ^{13}C NMR and DEPT-135 spectrum of 2-butyryloxybriarane B-3 (**2**) (500 MHz, $CDCl_3$)

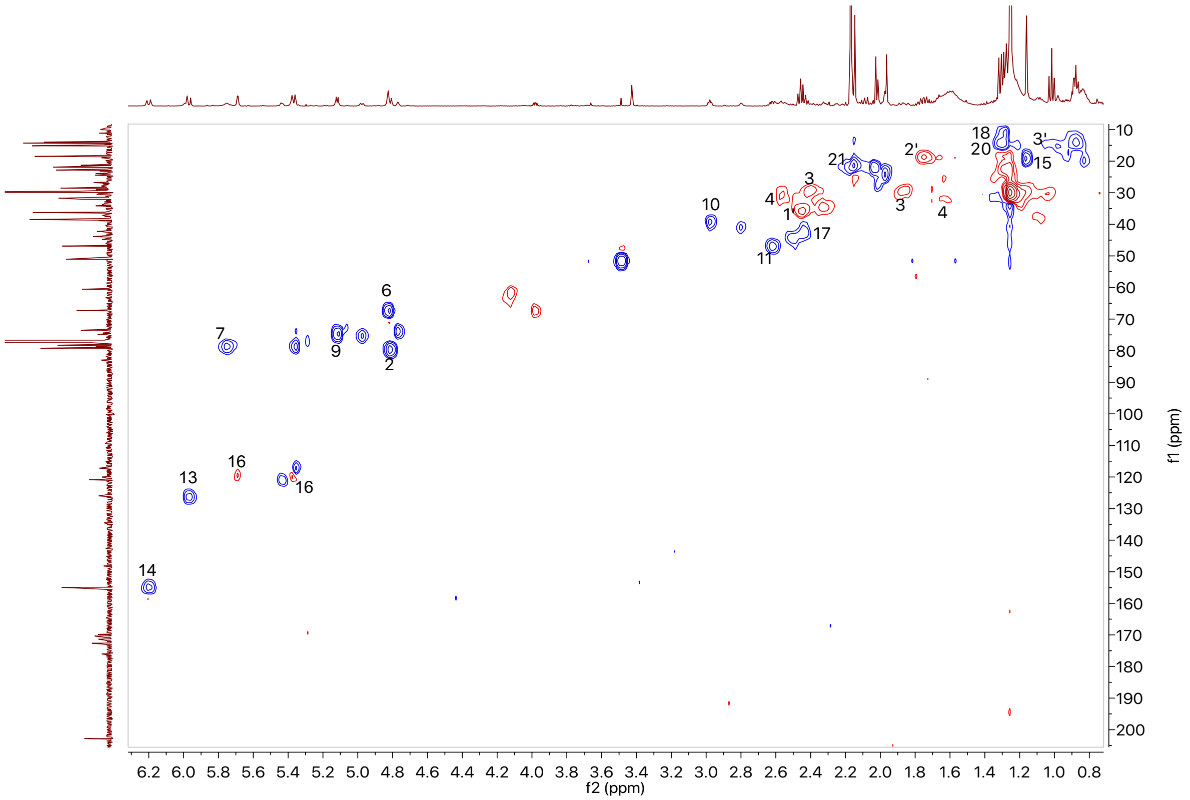

Figure S11. HSQC spectrum of 2-butyryloxybriarane B-3 (**2**) (500 MHz, $CDCl_3$). CH2: blue cross-peaks and CH or CH3: red cross-peaks.

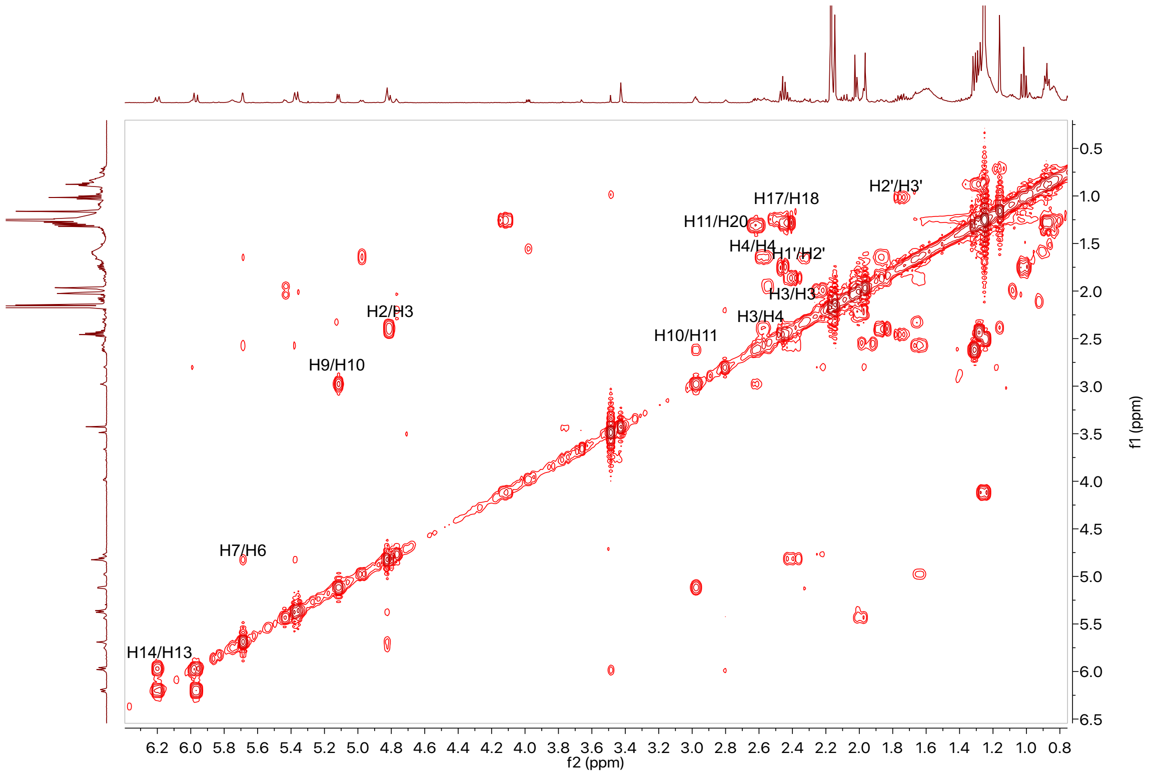

Figure S12. COSY spectrum of 2-butyryloxybriarane B-3 (**2**) (500 MHz, $CDCl_3$)

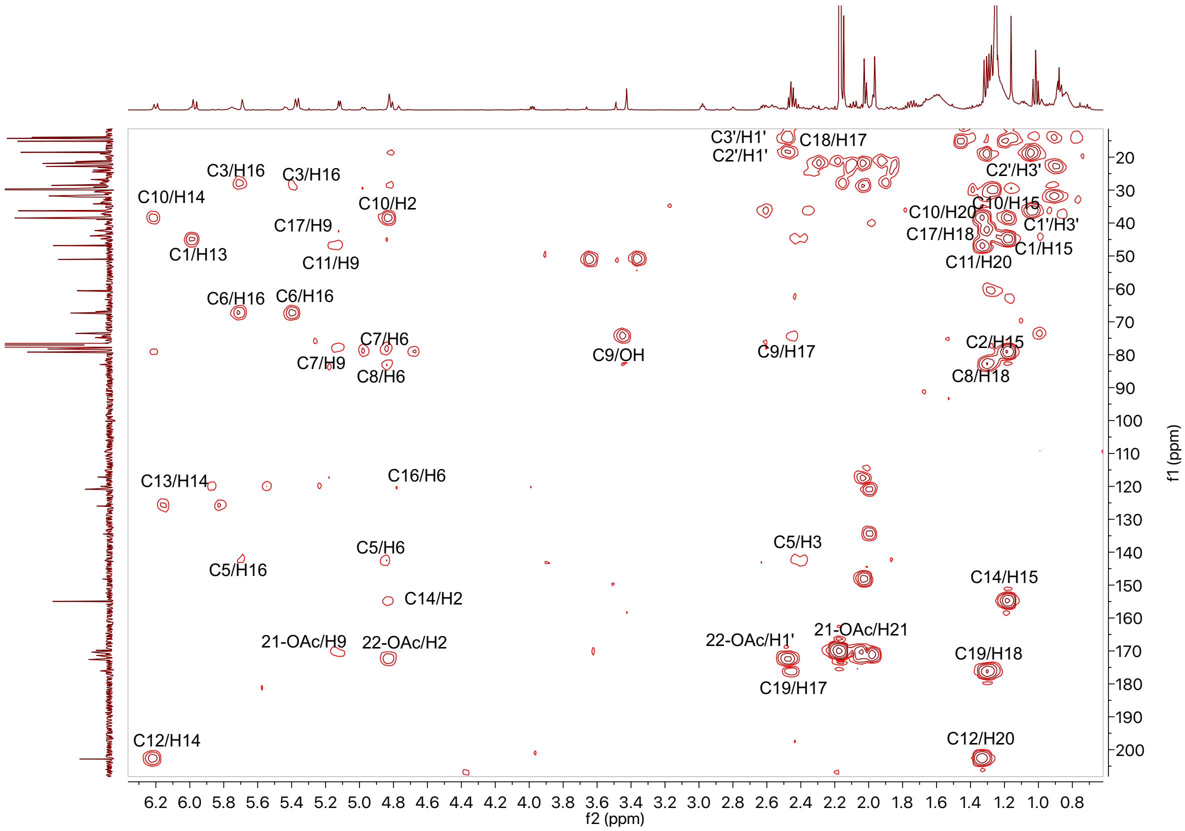

Figure S13. HMBC spectrum of 2-butyryloxybriarane B-3 (**2**) (500 MHz, $CDCl_3$)

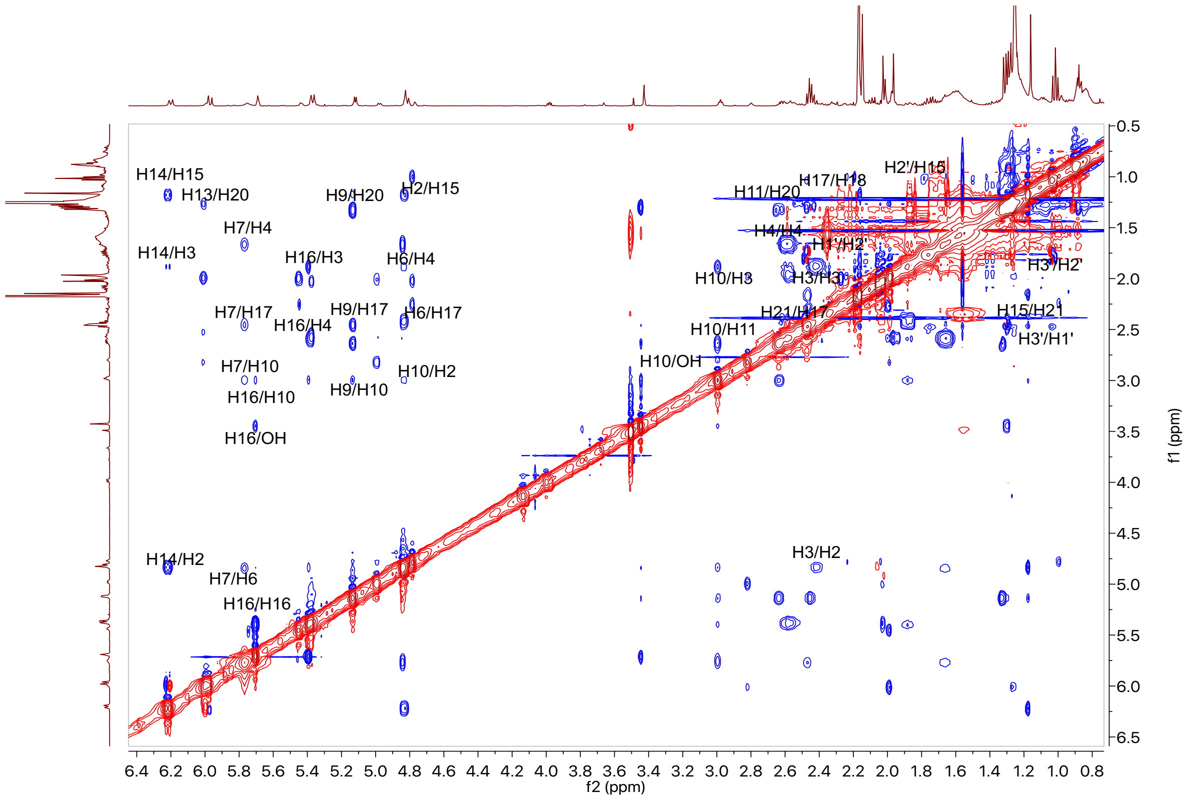

Figure S14. NOESY spectrum of 2-butyryloxybriarane B-3 (**2**) (500 MHz, $CDCl_3$)

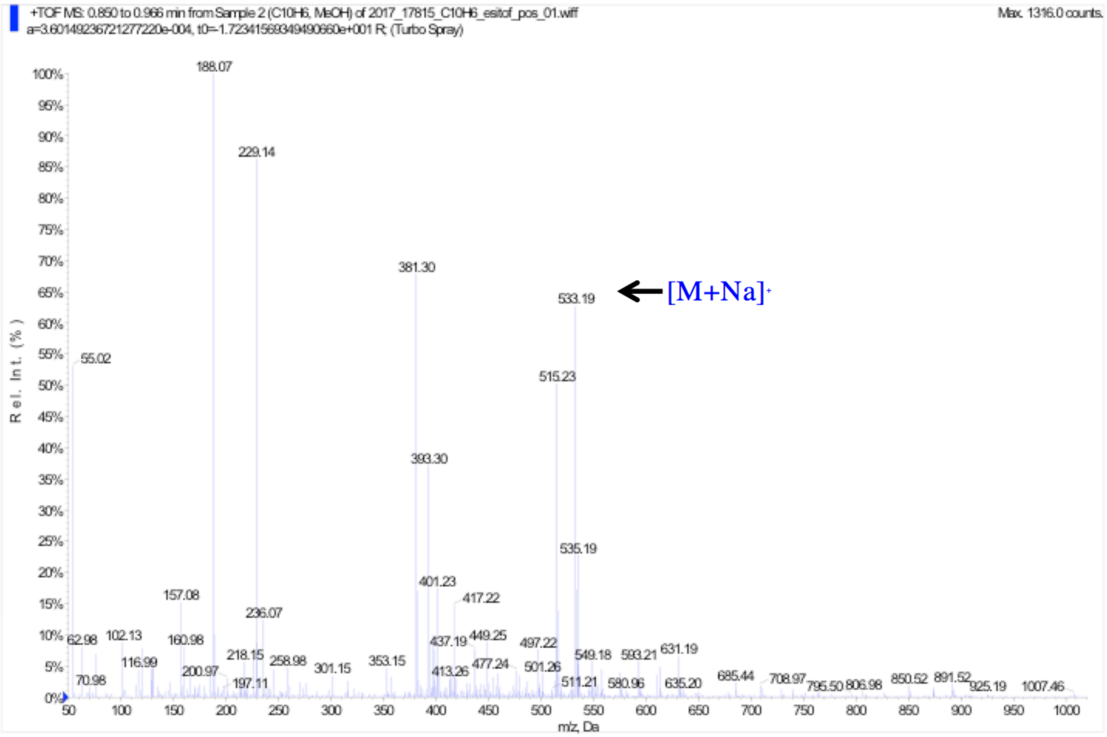

Figure S15. (+)-LRESIMS of 2-butyryloxybriarane B-3 (**2**)

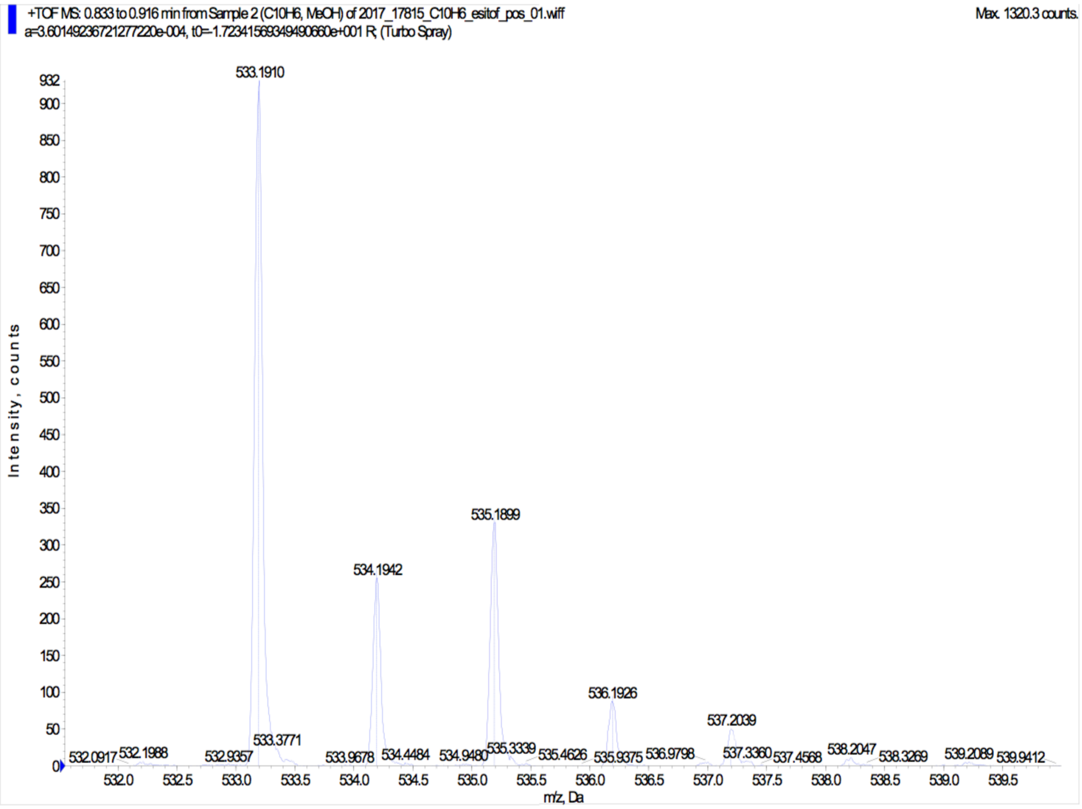

Figure S16. (+)-HRESIMS of 2-butyryloxybriarane B-3 (**2**)

Table S5. NMR data of 9-acetylbriarenolide S (**3**) in $CDCl_3$ (500 MHz)

no.	δ_C type	δ_H, mult. (*J* in Hz)	COSY	HMBC	NOESY
1	44.2, qC			13, 14, 15	
2	79.6 CH	4.37, d (6.0)	3	15	3, 4, 10, 15
3	31.7, CH_2	2.78, dt (15.4, 15.4, 5.5)-1.77, m	2, 4		2, 3, 7
4	26.2, CH_2	2.56, m-2.05, m	3	2, 6, 16	2, 4, 7, 16
5	144.2, qC			7, 16	
6	123.3, CH	5.88, dd (10.3, 0.6)	7	16	7
7	77.4, CH	5.23, d (10.3)	6		3, 4, 6, 17
8	82.6, qC			18	
9	71.1, CH	5.28, d (4.7)	10	17	10, 17, 15, 20
10	38.3, CH	2.64, dd (4.6, 4.7)	9, 11	14, 15, 20	2, 9, 16,
11	48.6, CH	2.53, m	10, 20	9, 20	15, 21
12	203.1, qC			14, 20	
13	124.3, CH	5.84, d (10.5)	14		14
14	154.8, CH	6.40, d (10.5)	13	15	13, 15
15	15.4, CH	1.22, s			2, 9, 11,14, 17,
16	51.2, CH_2	4.32, d (12.0)-4.18, d (12.0)		6	4, 10,
17	42.6, CH	2.45, q (7.2)	18		7, 9, 15, 18, 20, 22
18	6.8, CH	1.19, d (7.2)	17	17	9, 17
19	175.7, qC			17, 18	
20	15.2, CH	1.30, d (7.4)	11		9, 17, 18
21 Ac	169.0, qC			9	
22 Ac	170.7, qC			2	
CH_3 21	21.3, CH_3	2.25, s		21	11
CH_3 22	21.9, CH_3	2.14, s		22	4, 17
OH					

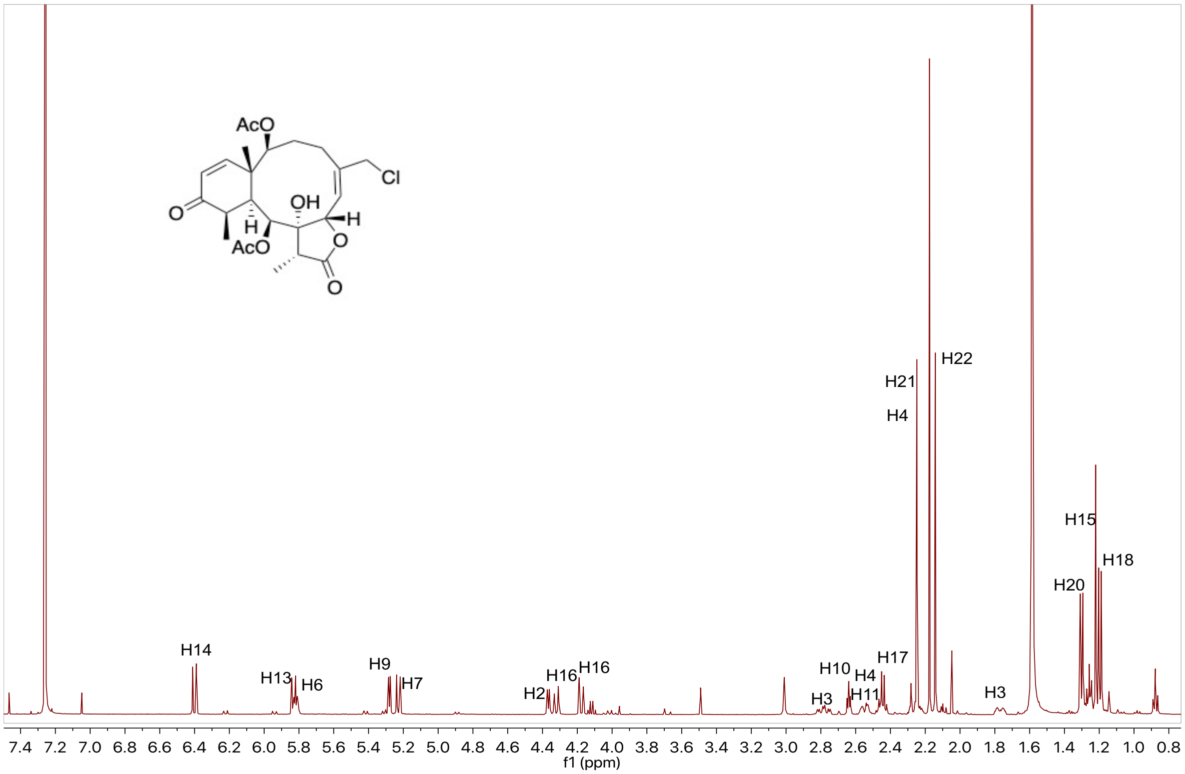

Figure S17. ^{1}H NMR spectrum of 9-acetylbriarenolide S (**3**) (500 MHz, $CDCl_3$)

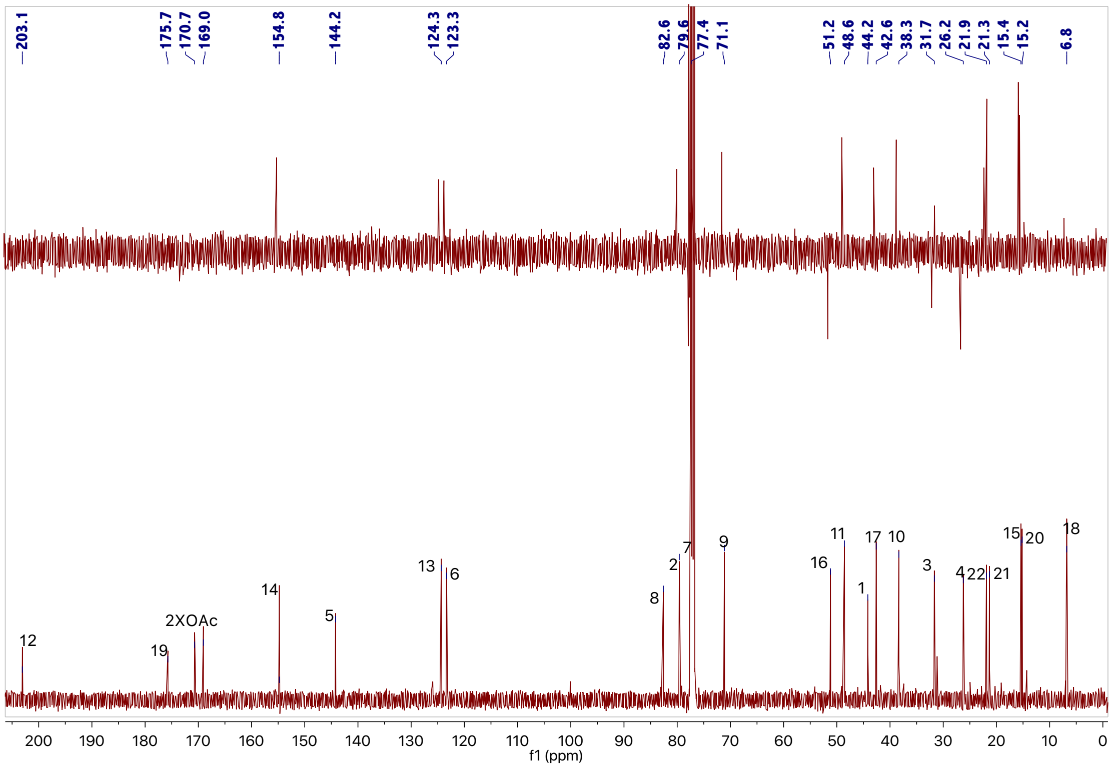

Figure S18. ^{13}C NMR and DEPT-135 spectrum of 9-acetylbriarenolide S 5 (**3**) (125 MHz, $CDCl_3$)

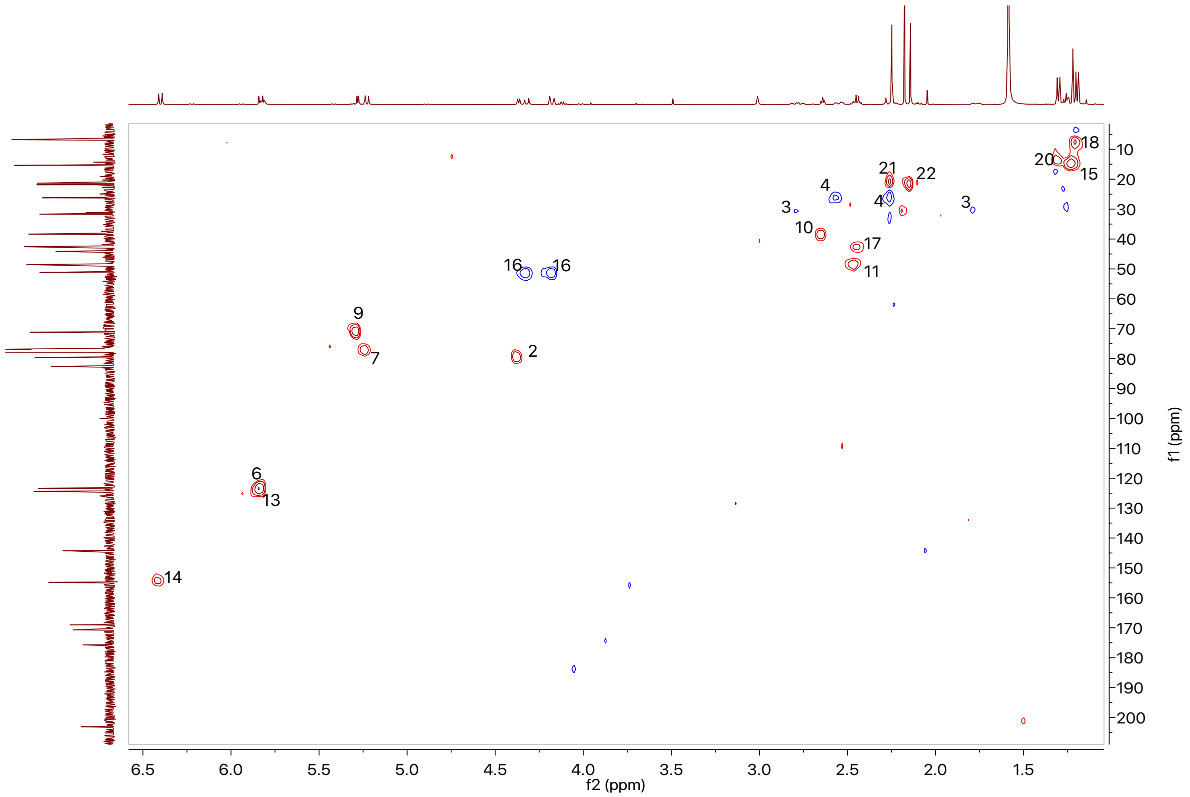

Figure S19. Edited-HSQC spectrum of 9-acetylbriarenolide S (**3**) (500 MHz, $CDCl_3$). CH_2: blue cross-peaks and CH or CH_3: red cross-peaks.

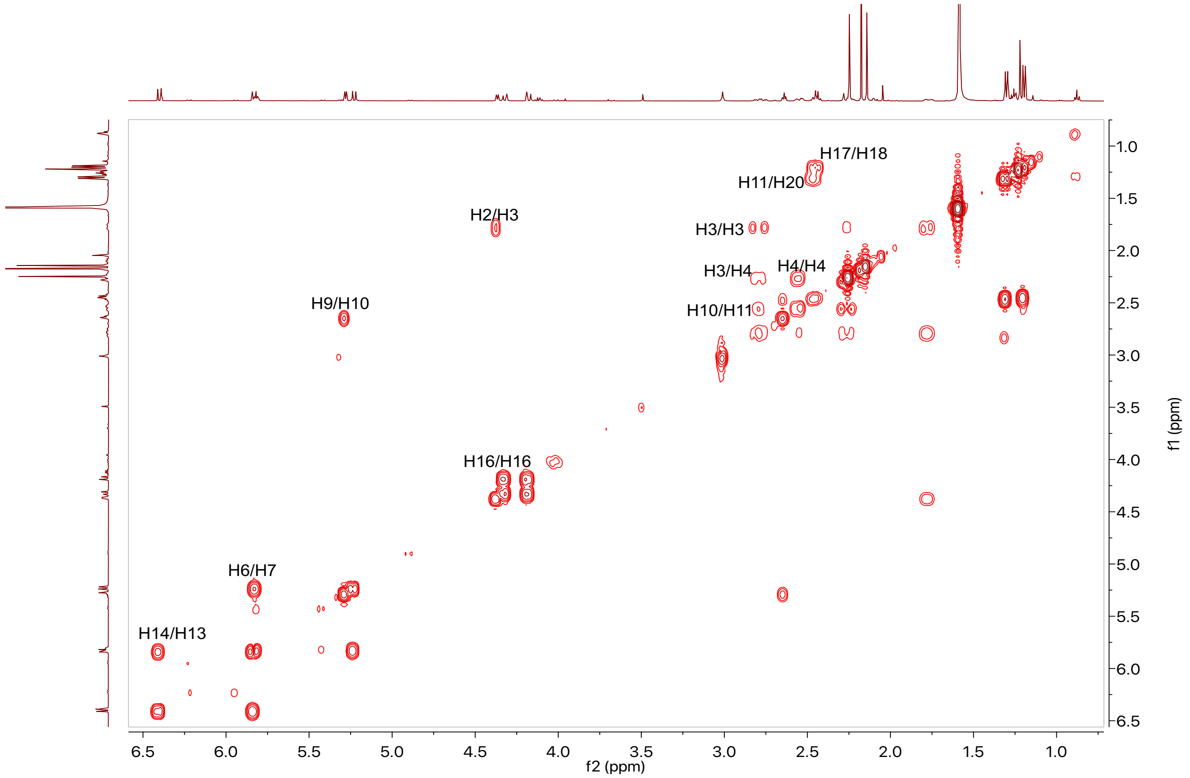

Figure S20. COSY spectrum of 9-acetylbriarenolide S (**3**) (500 MHz, $CDCl_3$)

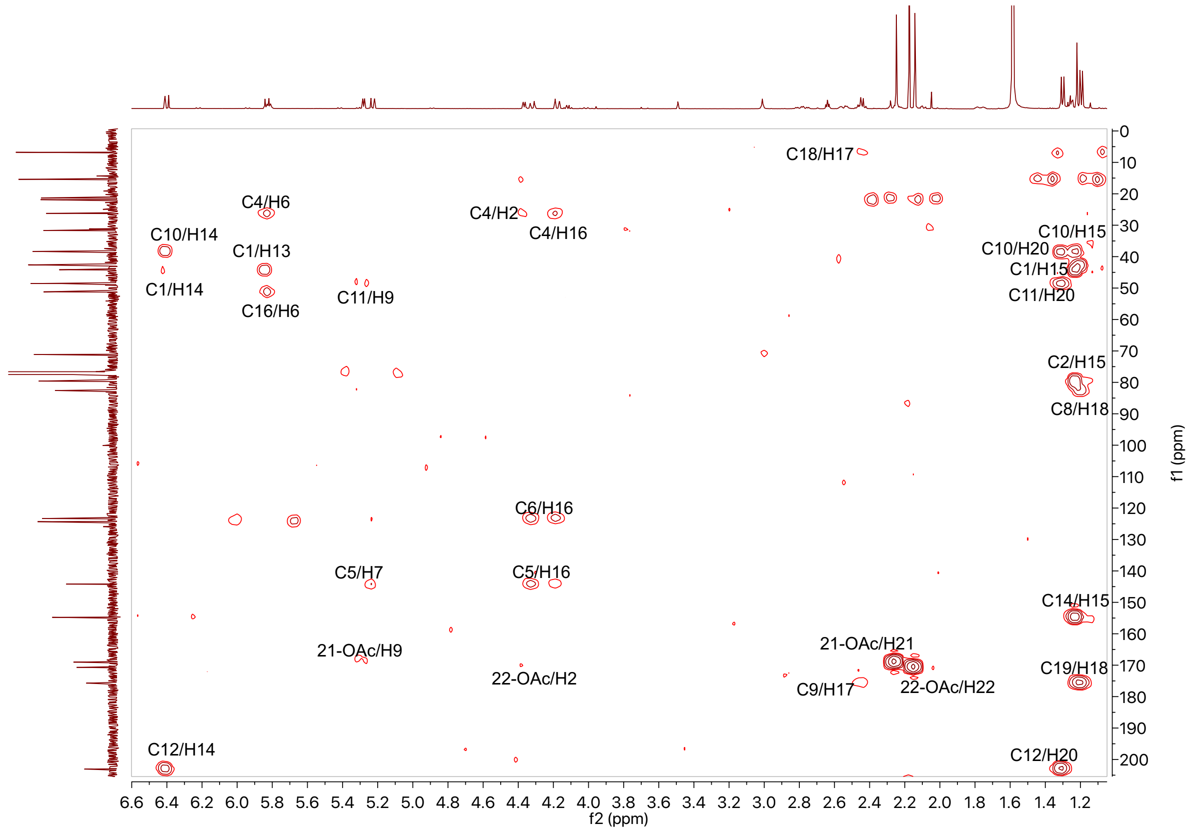

Figure S21. HMBC spectrum of 9-acetylbriarenolide S (**3**) (500 MHz, $CDCl_3$)

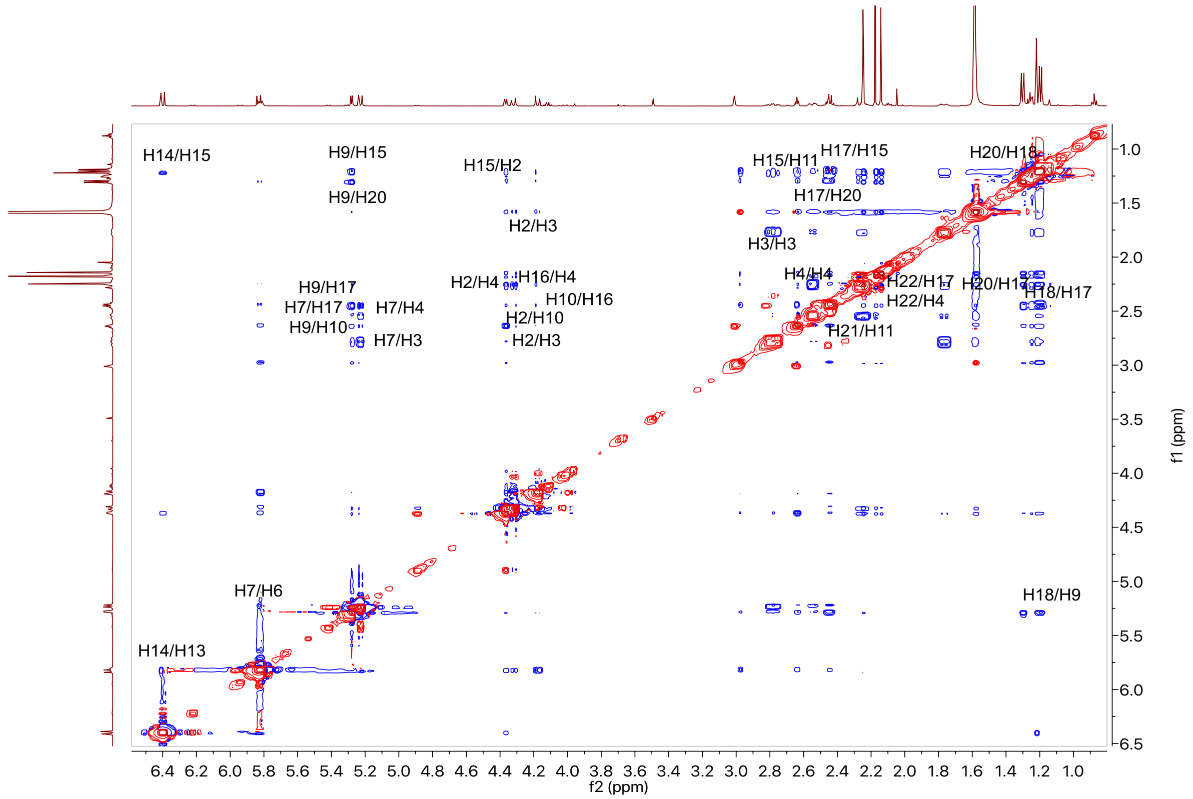

Figure S22. NOESY spectrum of 9-acetylbriarenolide S (**3**) (500 MHz, $CDCl_3$)

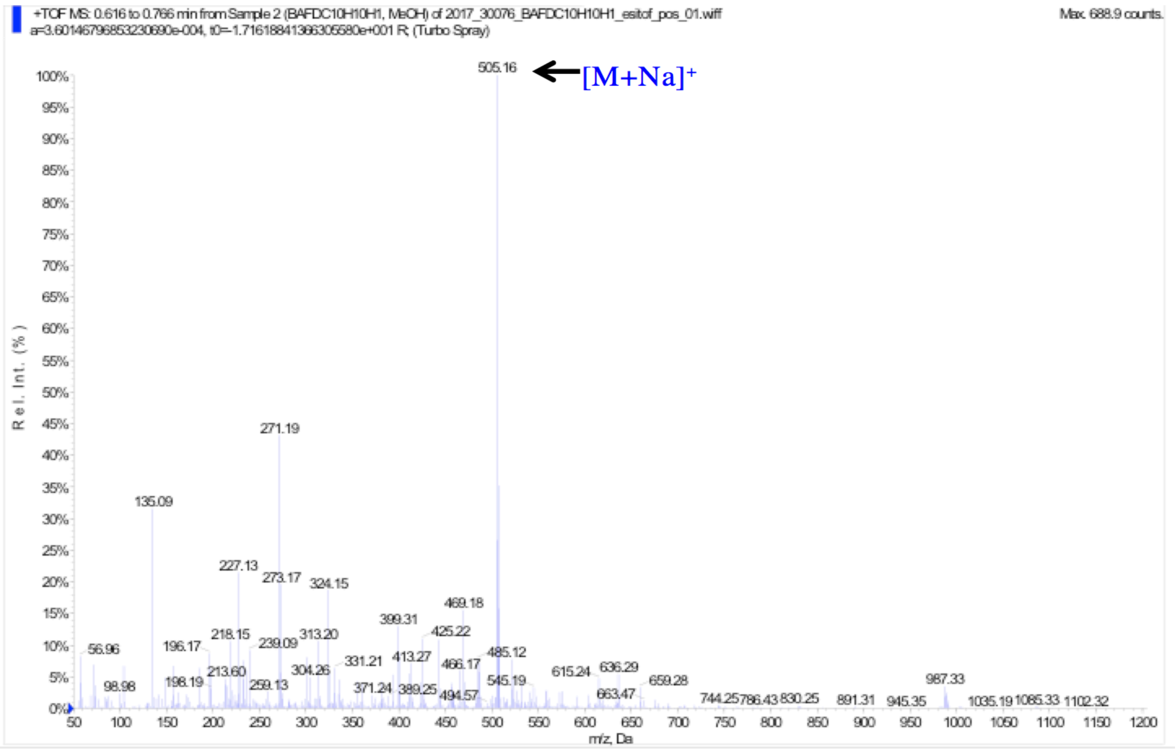

Figure S23. (+)-LRESIMS of 9-acetylbriarenolide S (**3**)

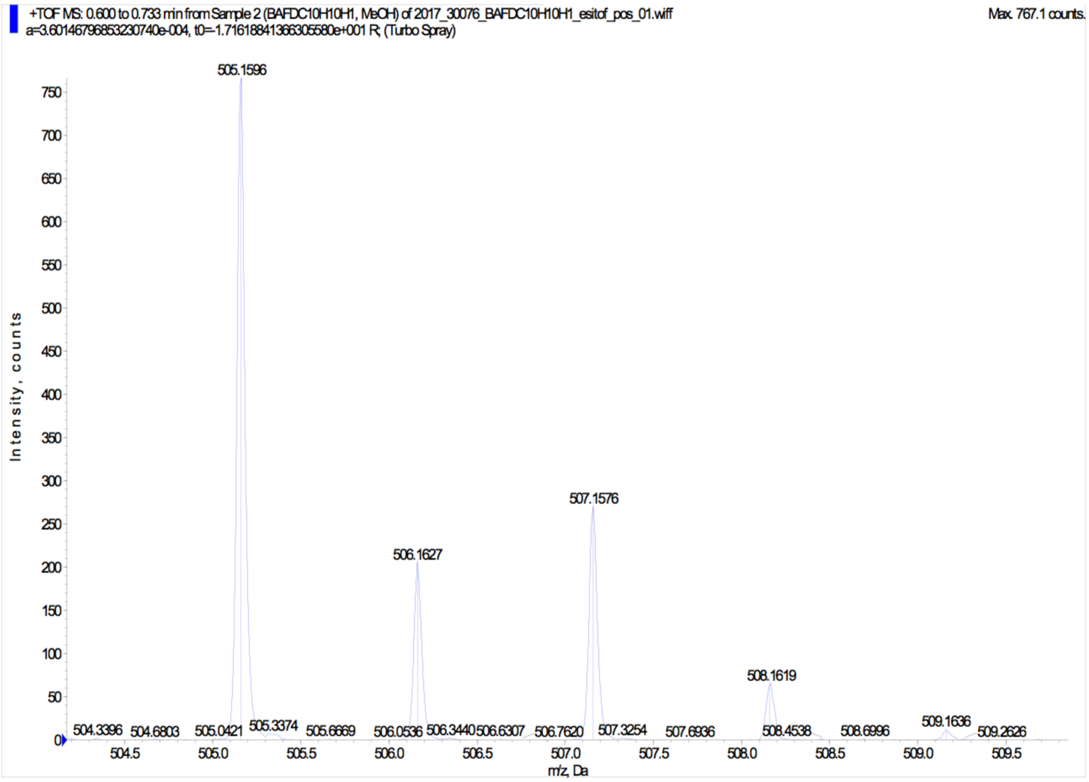

Figure S24. (+)-HRESIMS of 9-acetylbriarenolide S (**3**)

Table S6. NMR data of briarenolide W (**4**) in $CDCl_3$ (500 MHz)

no.	δ_C type	δ_H, mult. (*J* in Hz)	COSY	HMBC	NOESY
1	44.2, qC			13, 14, 15	
2	80.6 CH	4.40, d (5.4)	3	14, 15	3, 4, 10, 14, 15
3	31.7, CH_2	2.85, dt (15.5, 15.5, 4.9)-1.74, m	2, 4		2, 3, 6, 7
4	25.5, CH_2	2.55, m-2.00, m	3	2, 6, 16	2, 4, 9
5	147.8, qC			7, 16	
6	118.3, CH	5.72, d (10.2)	7	16	3, 7, 16
7	77.5, CH	5.28, d (10.2)	6		3, 6, 17
8	82.4, qC			9, 18	
9	71.2, CH	5.30, d (5.2)	10		10, 17, 18, 20
10	37.9, CH	2.77, t (5.2)	9, 11	14, 15, 20	2, 9, 11, 17
11	48.1, CH	2.52, m	10, 20	9, 13, 20	10, 20
12	203.9, qC			14, 20	
13	124.2, CH	5.80, d (10.5)	14		14
14	155.4, CH	6.40, d (10.5)	13	20	2, 13, 15, 22
15	6.8, CH	1.20, s			2,14, 17, 21
16	67.5, CH_2	4.32, d (15.8)-4.07, d (15.8)		6	6
17	42.8, CH	2.41, q (7.1)	18		7, 9, 15, 18
18	15.4, CH	1.19, d (7.1)	17		9
19	176.7, qC			17, 18	
20	15.3, CH	1.29, d (7.4)	11		9, 11
21 Ac	169.1, qC			9	
22 Ac	171.1, qC			2	
CH_3 21	21.2, CH_3	2.24, s		21	
CH_3 22	21.9, CH_3	2.14, s		22	14
OH					

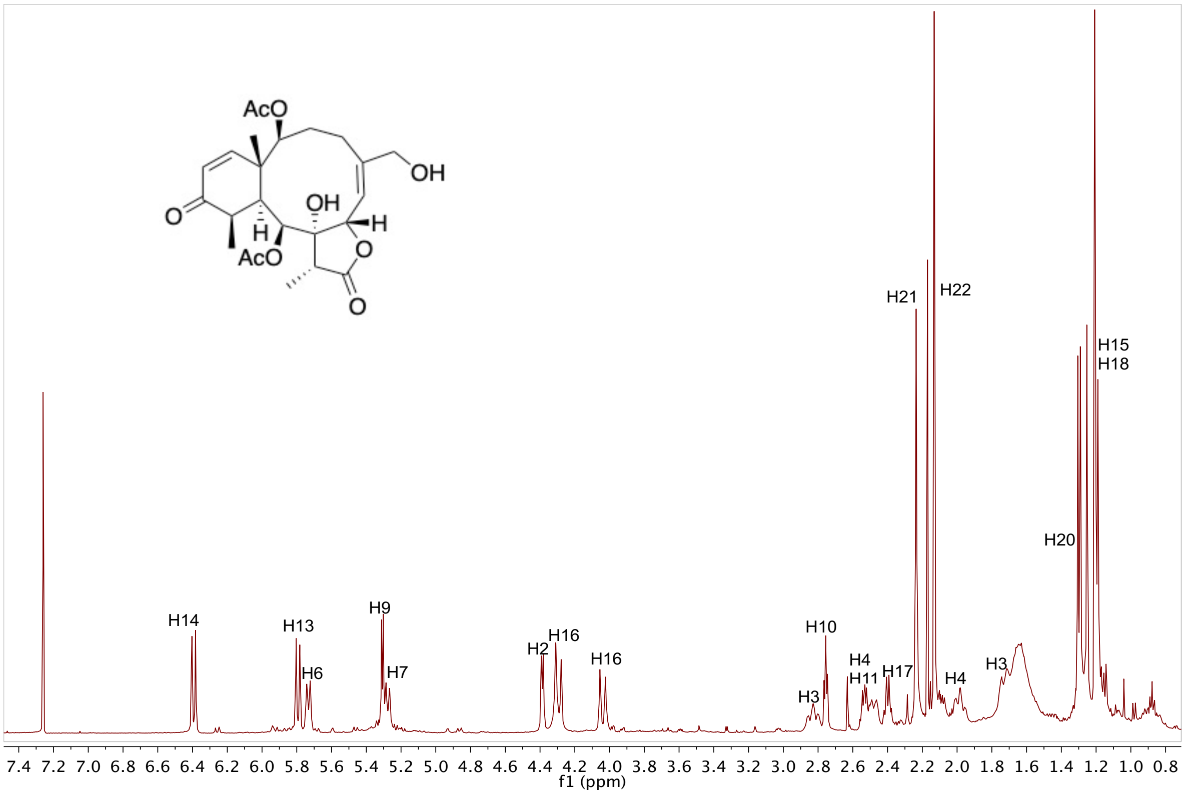

Figure S25. ^{1}H NMR spectrum of briarenolide W (**4**) (500 MHz, $CDCl_3$)

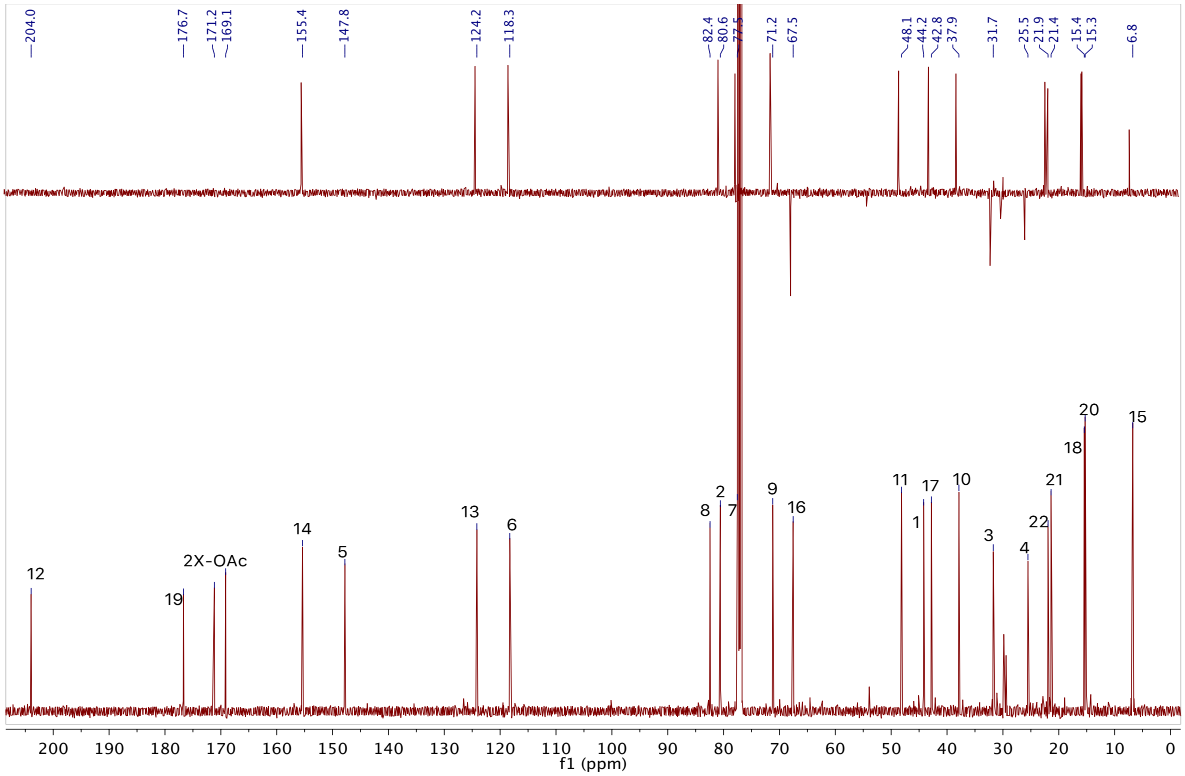

Figure S26. ^{13}C NMR and DEPT-135 spectrum of briarenolide W (**4**) (500 MHz, $CDCl_3$)

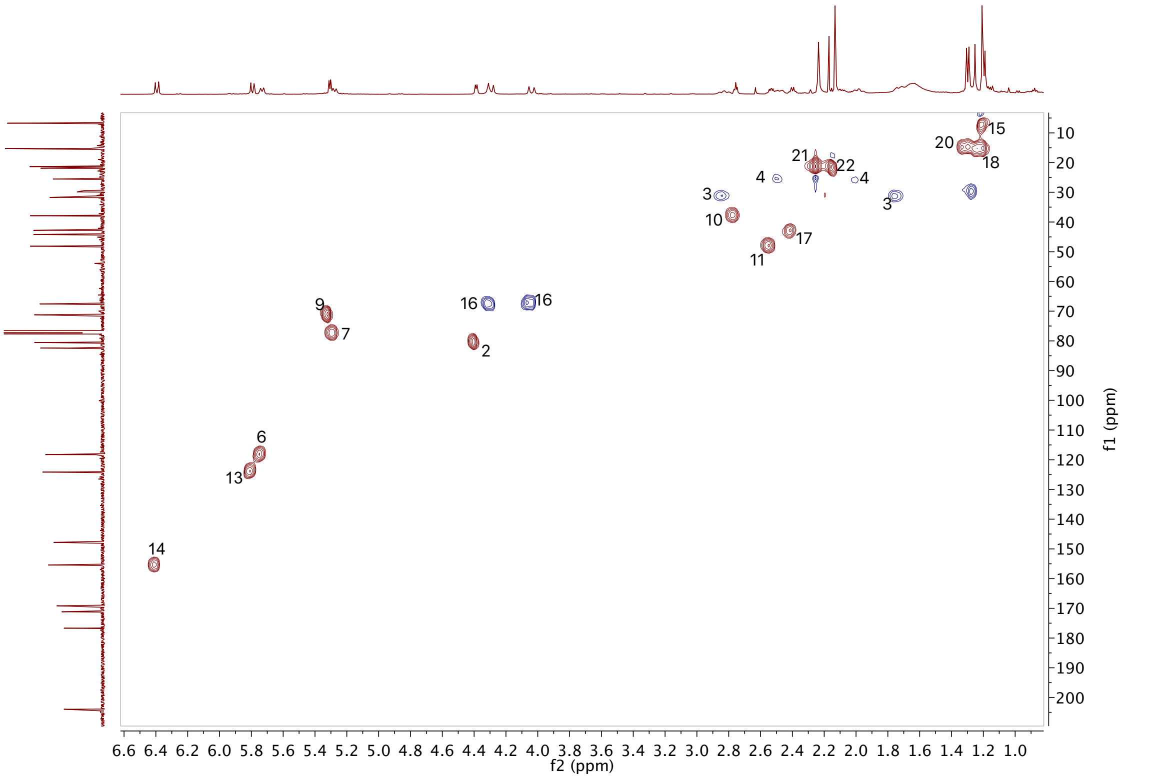

Figure S27. Edited-HSQC spectrum of briarenolide W (**4**) (500 MHz, $CDCl_3$). CH_2: blue cross-peaks and CH or CH_3: red cross-peaks.

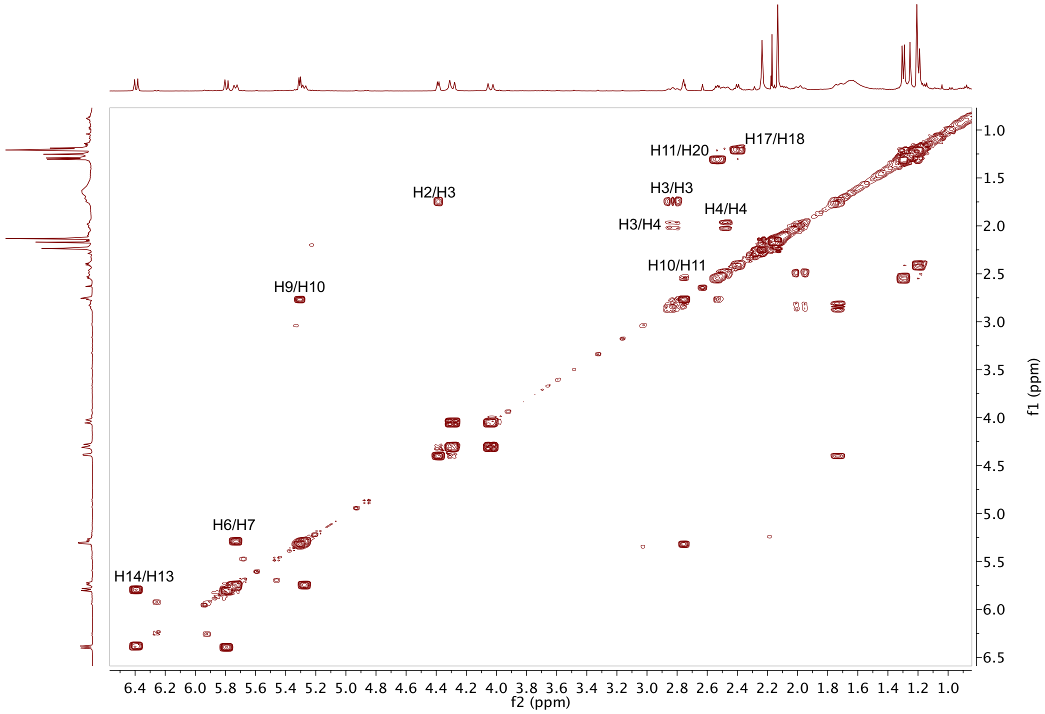

Figure S28. COSY spectrum of briarenolide W (**4**) (500 MHz, $CDCl_3$)

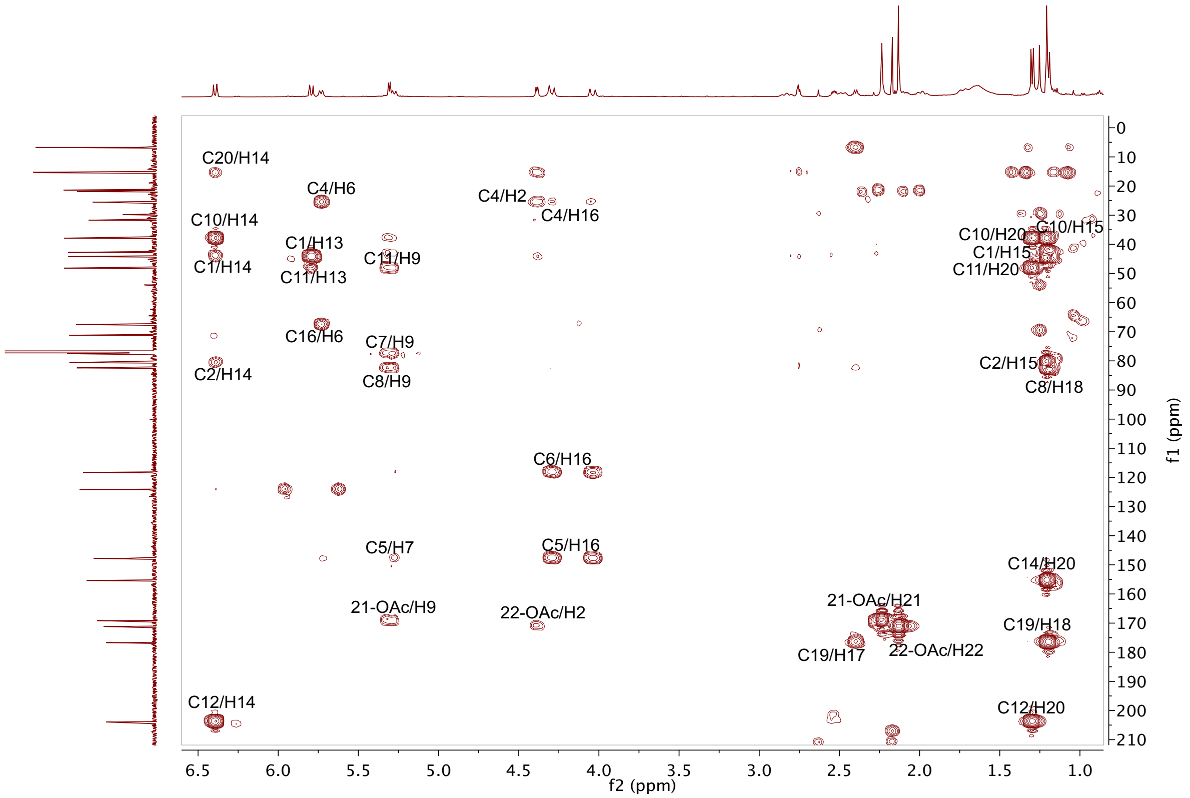

Figure S29. HMBC spectrum of briarenolide W (**4**) (500 MHz, $CDCl_3$)

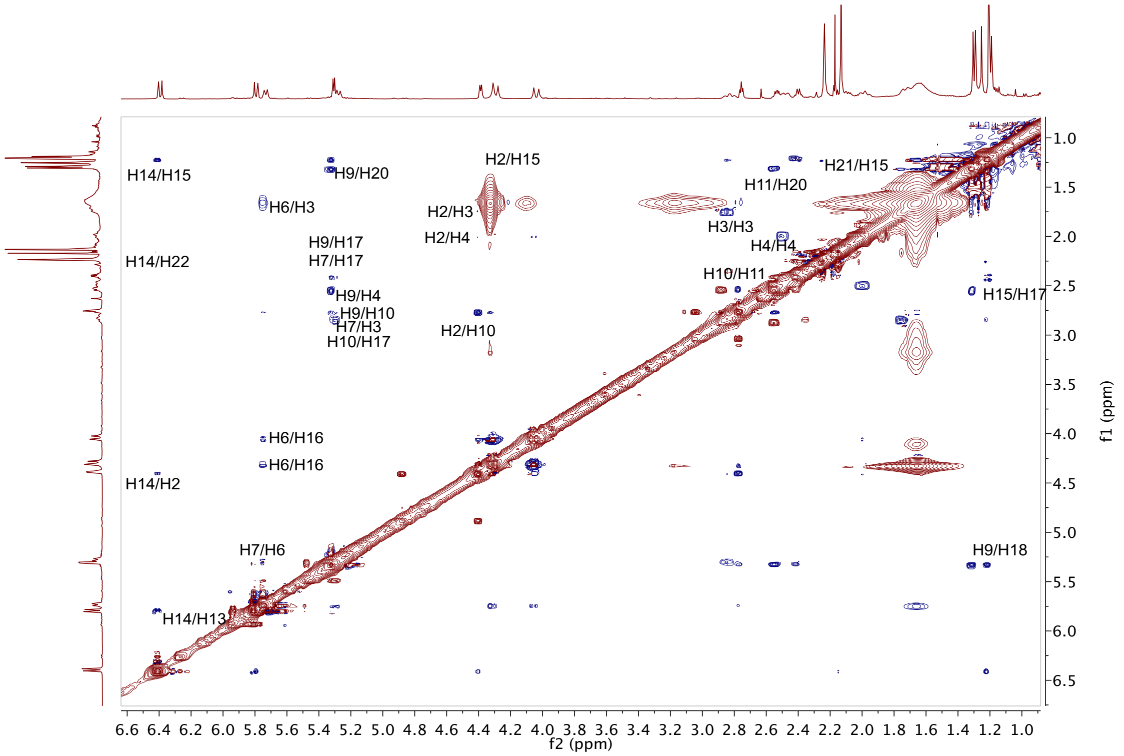

Figure S30. NOESY spectrum of briarenolide W (**4**) (500 MHz, $CDCl_3$)

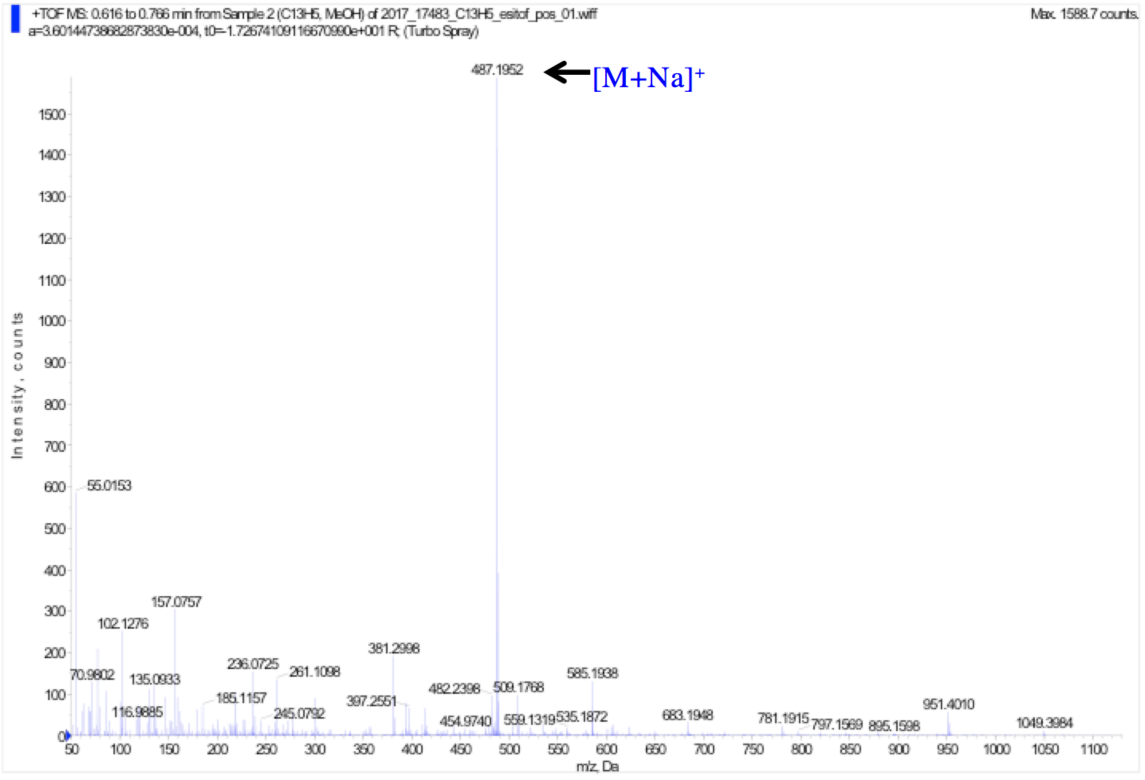

Figure S31. (+)-LRESIMS of briarenolide W (**4**)

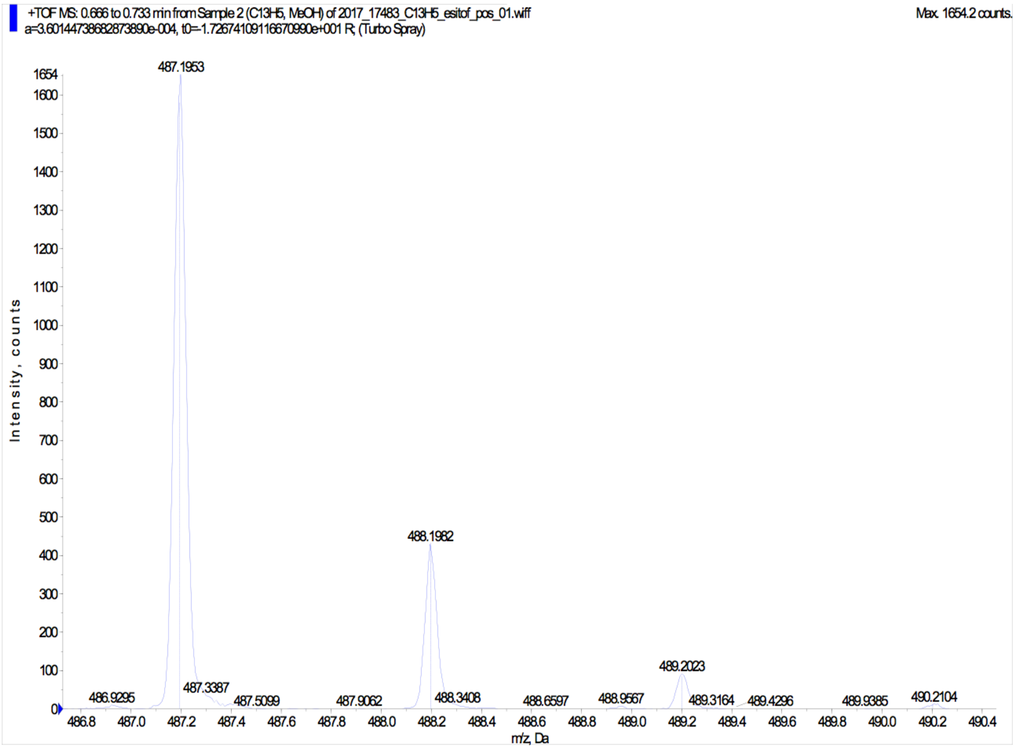

Figure S32. (+)-HRESIMS of briarenolide W (**4**)

Table S7. NMR data of 12-isobriarenolide P (**5**) in $CDCl_3$ (500 MHz)

**no.**	**δ_C type**	**δ_H, mult. (*J* in Hz)**	**COSY**	**HMBC**	**NOESY**
1	40.4, qC			14, 15	
2	76.5 CH	4.27, d (9.7)	3	4, 10, 15	3, 10
3	137.2, CH_2	5.80, dd (11.0, 9.7)	2, 4		2, 15, 21
4	125.0, CH_2	6.21, dd (11.0, 1.2)	3	6, 16	3, 7
5	141.2, qC			3, 7, 16	
6	126.7, CH	5.83, dq (8.6, 1.2, 1.2, 1.2)	7	4	16
7	79.2, CH	5.07, d (8.6)	6	9	4, 9, 17
8	82.5, qC			9, 10, 18	
9	70.0, CH	5.14, d (6.7)	10	10	10, 11, 15, 17, 18, 20
10	31.3, CH	2.12, d (6.7)	9, 11	12, 20	2, 9, 11, 16
11	41.7, CH	1.80, m	10, 12, 20	9, 20	9, 10, 12, 20
12	66.7, qC	3.94, dd (5.9, 2.3)	11, 13	20	11, 13, 20
13	55.2, CH	3.56, dd (5.9, 3.6)	12,14		12, 20
14	64.4, CH	3.41, d (3.6)	13	15	2, 13, 15
15	14.7, CH	1.11, s		2, 10	3, 9, 14, 21
16	46.6, CH_2	4.24, bd (12.8)-4.18, bd (12.8)		6	6, 10
17	43.2, CH	2.31, d (7.1)	18		7, 9, 18
18	6.5, CH	1.17, d (7.1)	17		9, 17
19	175.7, qC			17, 18	
20	13.3, CH	1.01, d (7.5)	11		9, 11
21 Ac	169.9, qC			9	15
CH_3 21	21.9, CH_3	2.18, s		21	

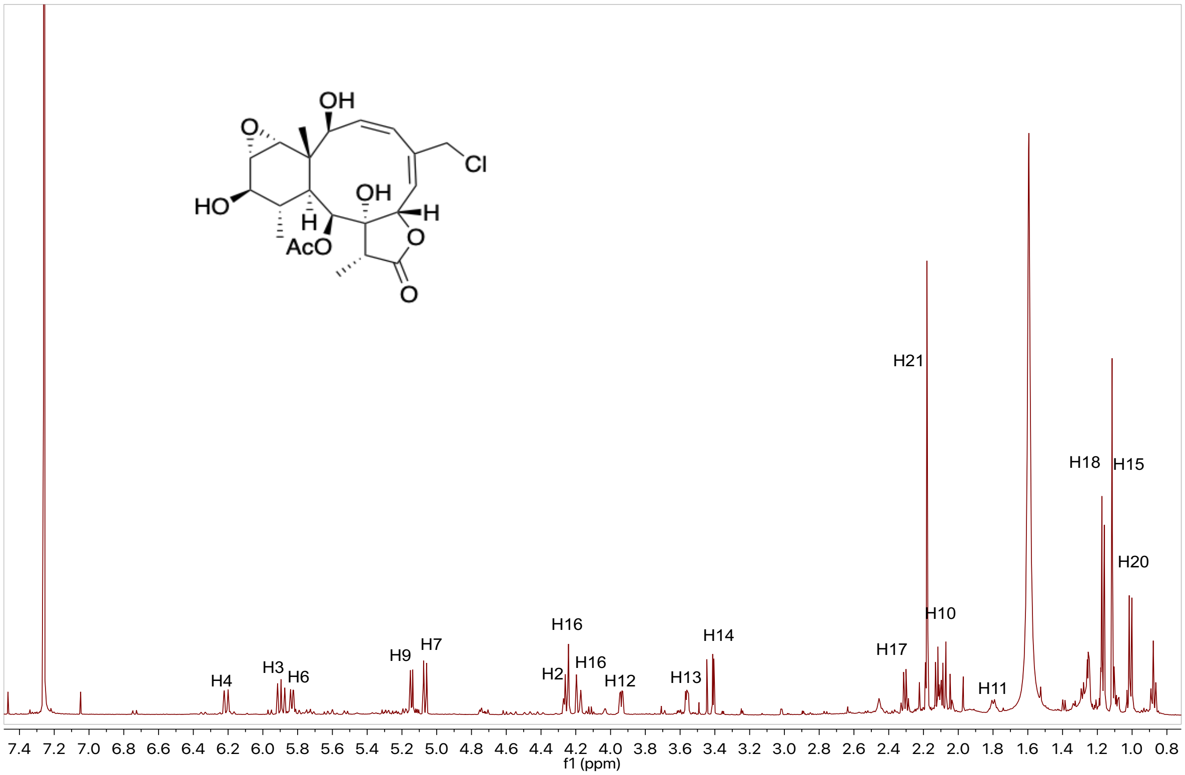

Figure S33. ^{1}H NMR spectrum 12-isobriarenolide P (**5**) (500 MHz, $CDCl_3$)

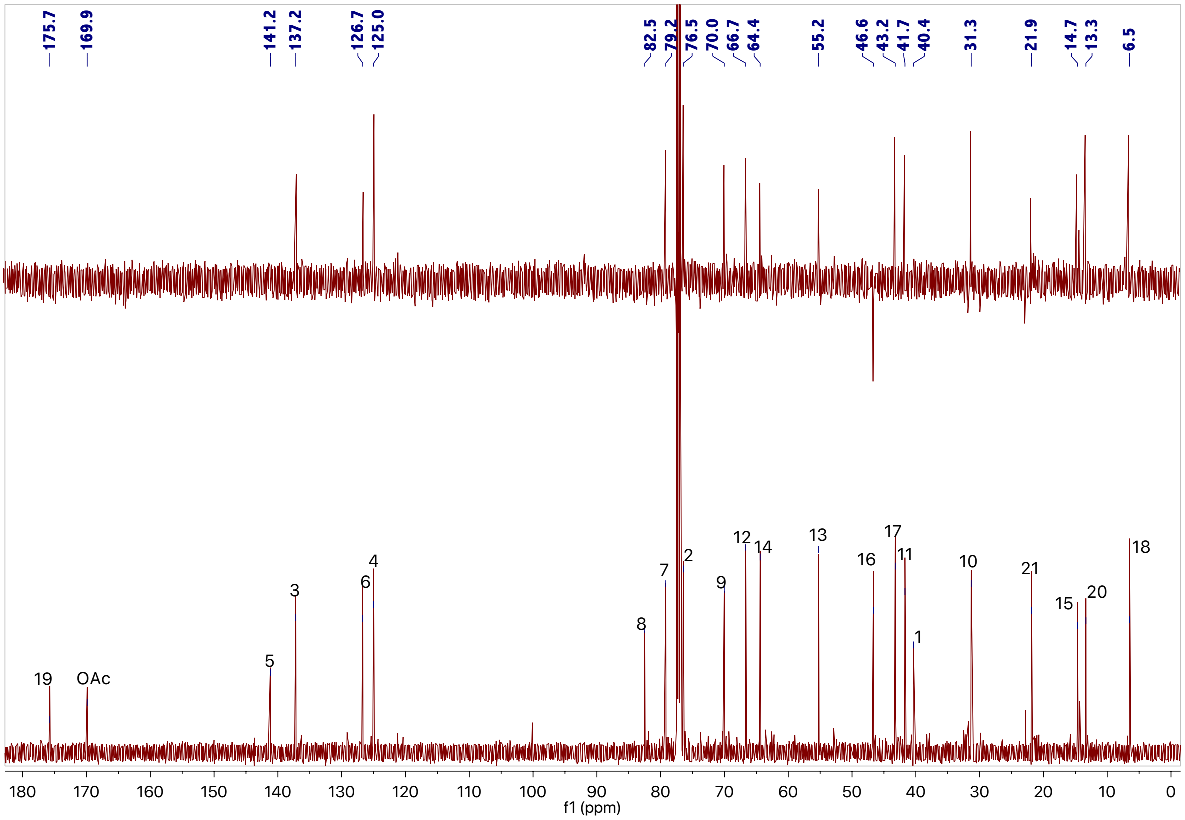

Figure S34. ^{13}C NMR and DEPT-135 spectrum of 12-isobriarenolide P (**5**) (500 MHz, $CDCl_3$)

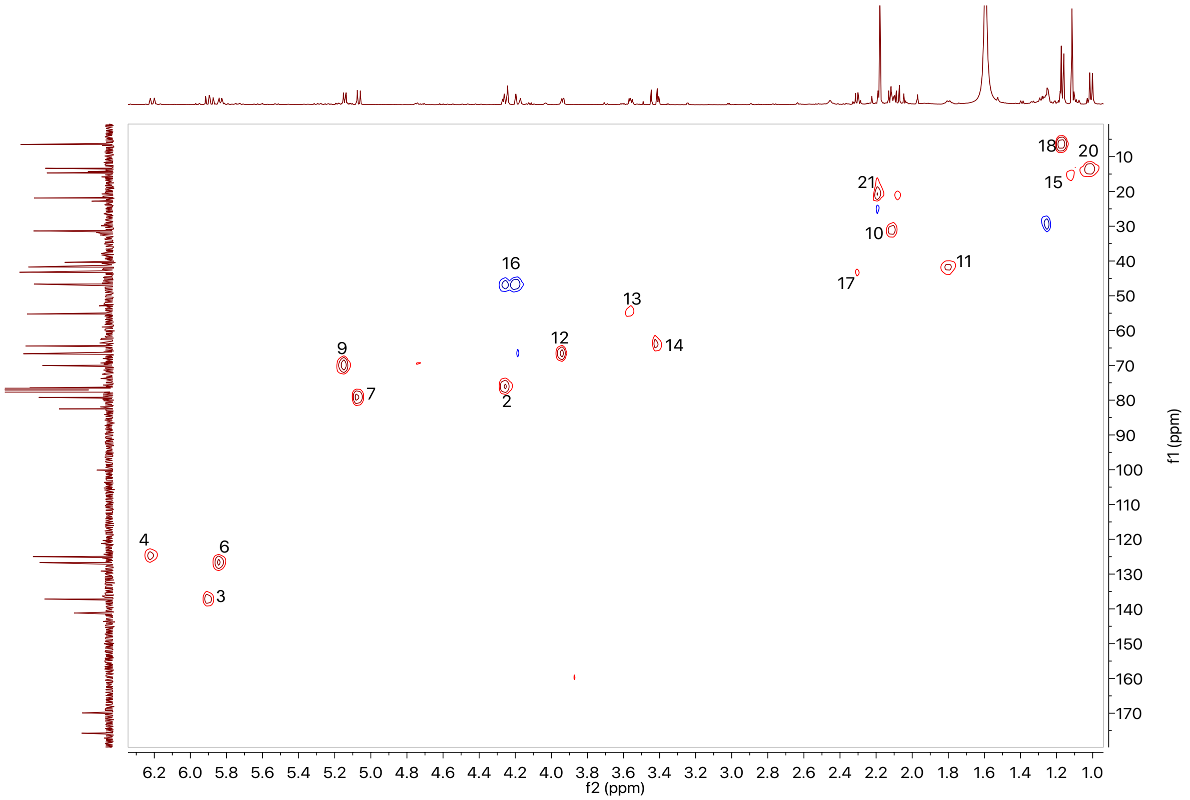

Figure S35. HSQC spectrum of 12-isobriarenolide P (**5**) (500 MHz, $CDCl_3$). CH_2: blue cross-peaks and CH or CH_3: red cross-peaks.

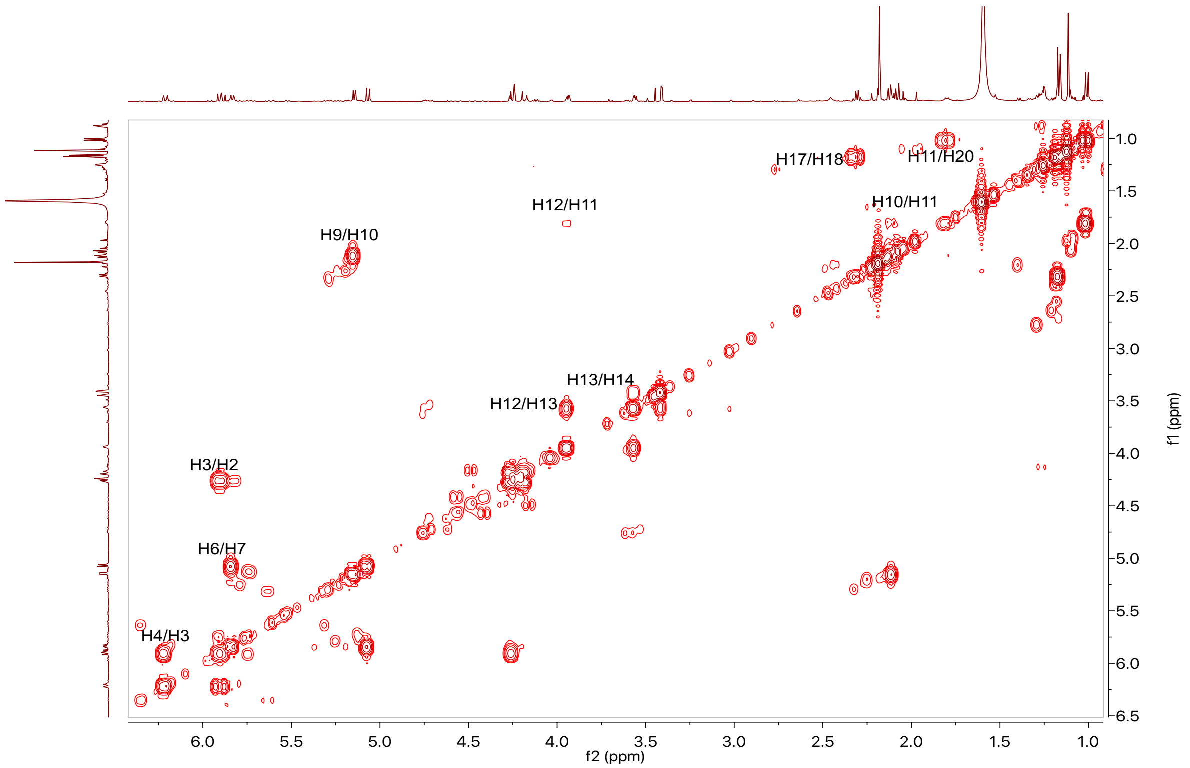

Figure S36. COSY spectrum of 12-isobriarenolide P (**5**) (500 MHz, $CDCl_3$)

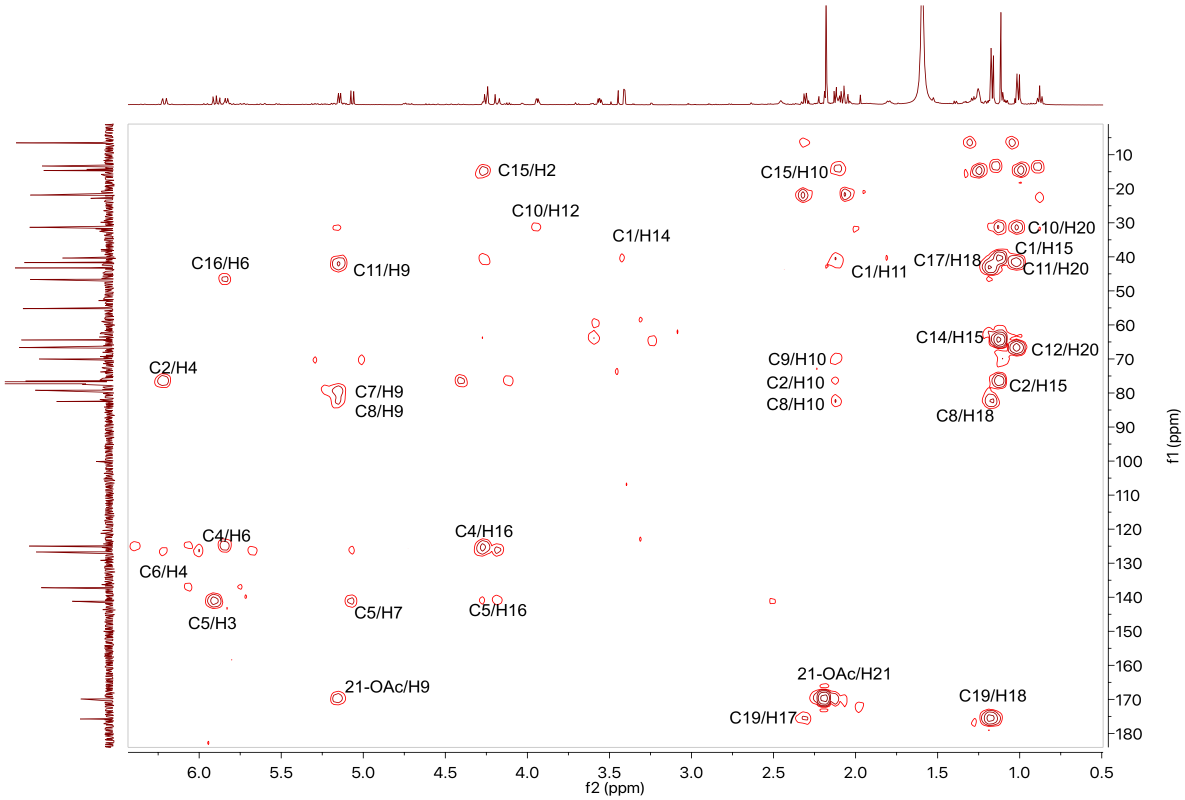

Figure S37. HMBC spectrum of 12-isobriarenolide P (**5**) (500 MHz, $CDCl_3$)

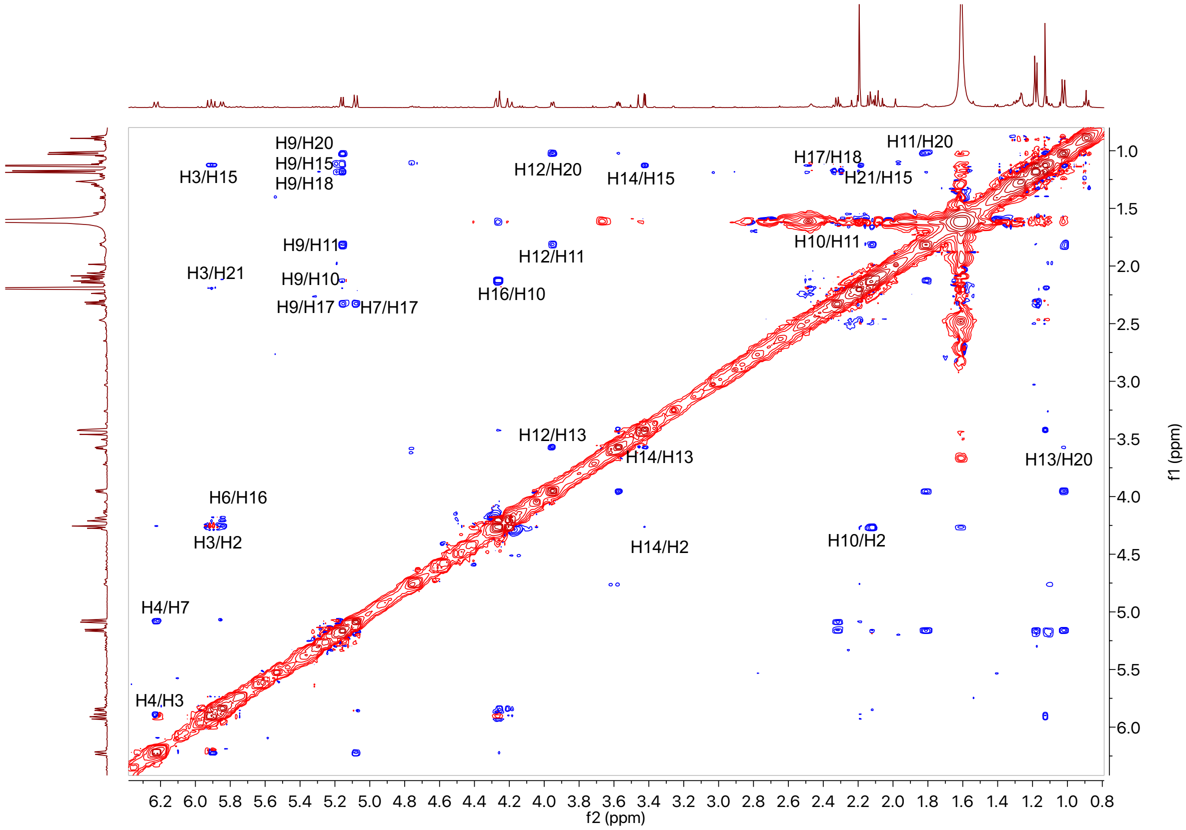

Figure S38. NOESY spectrum of 12-isobriarenolide P (**5**) (500 MHz, $CDCl_3$)

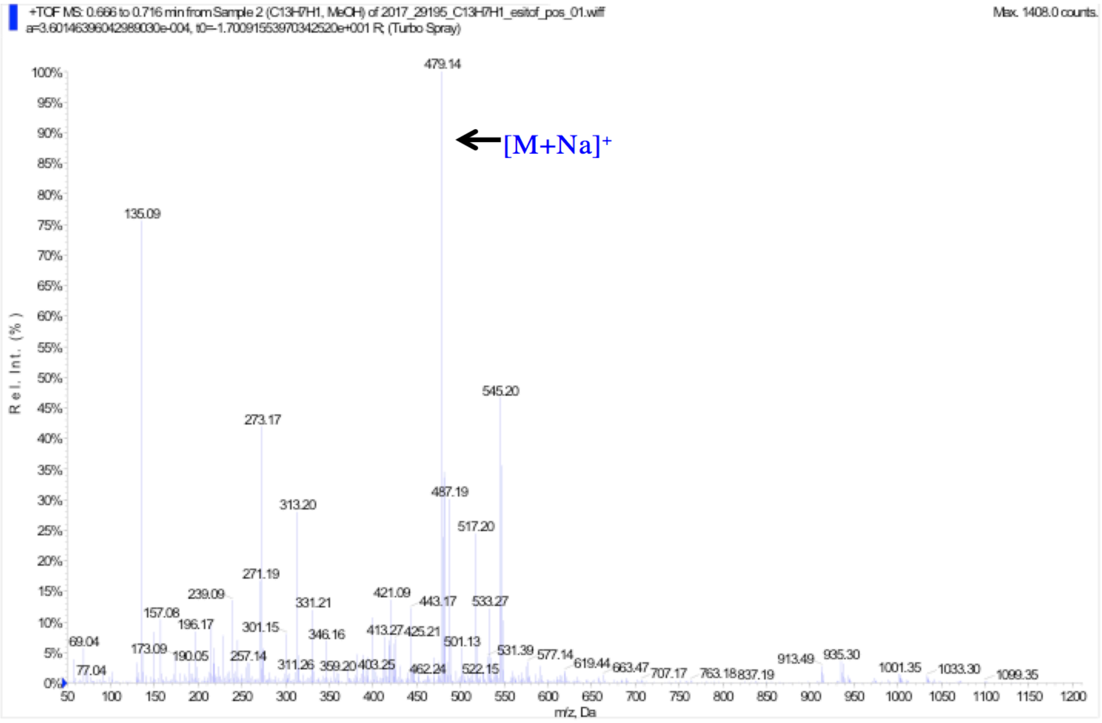

Figure S39. (+)-LRESIMS of 12-isobriarenolide P (**5**)

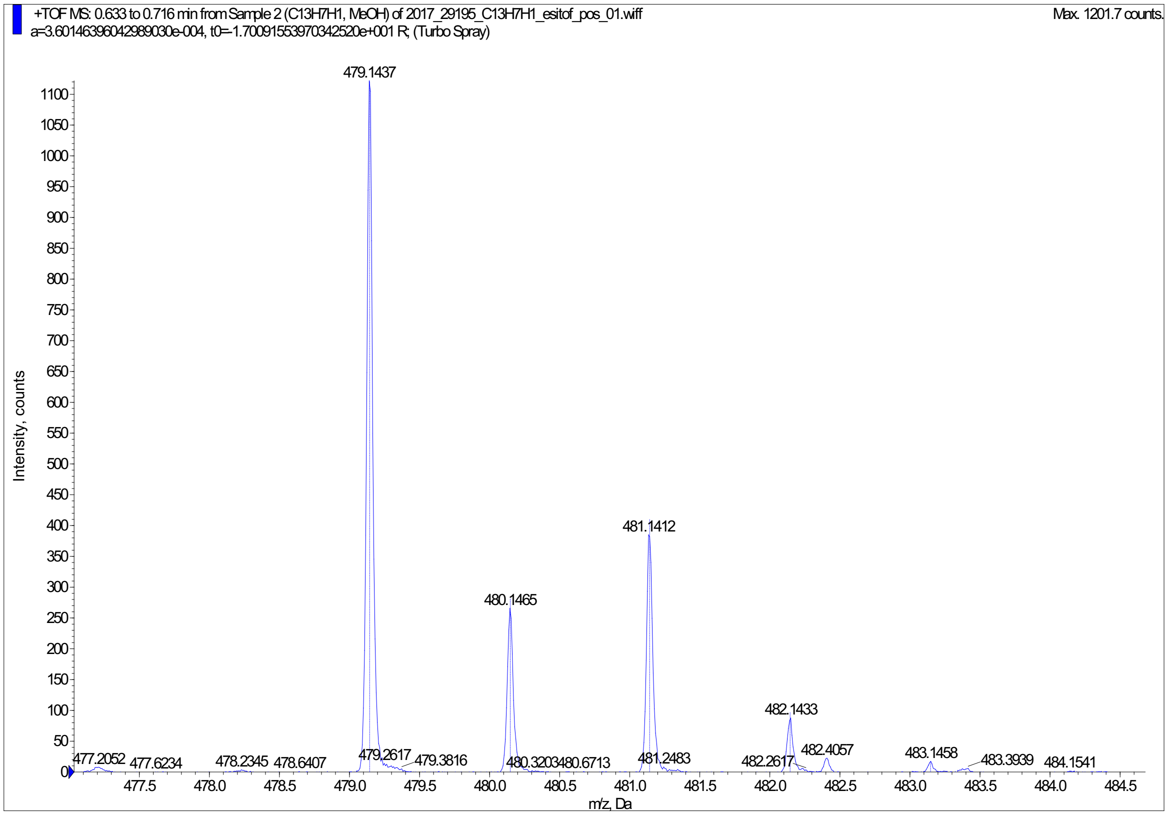

Figure S40. (+)-HRESIMS of 12-isobriarenolide P (**5**)

Table S8. NMR data of lactone 14 (**10**) in $CDCl_3$ (500 MHz)

no.	δ_C type	δ_H, mult. (J in Hz)	COSY	HMBC
1	44.5, qC			4, 14, 15
2	73.5, CH	4.77, d (3.4)	3	15, 23
3	28.8, CH_2	1.96, m-2.20, m	2, 4	14
4	29.9, CH_2	2.55, m-1.25, m	3	
5	148.3, qC			3, 16
6	120.9, CH	5.41, bs	7	2, 3
7	78.6, CH	5.35, bs	6	9
8	81.8, qC			9, 18
9	70.3, CH	5.99, bs	10	10, 21
10	40.4, CH	2.80, bs	9	20
11	134.5, qC			9, 10, 20
12	117.1, CH	5.36, bs	13	20
13	32.0, CH_2	2.56, t (3.8)-1.65 (bs)	12, 14	
14	74.8, CH	4.98, d (7.4)	13	13, 22
15	14.6, CH_3	0.98, s		10, 14
16	27.8, CH_3	2.02, s		3, 7
17	43.9, CH_3	2.51, d (7.2)	18	18
18	7.2, CH_3	1.21, d (6.6)	17	17
19	176.0, qC			17, 18
20	26.8, CH_3	1.97, s		12
21 Ac	169.8, qC			9
22 Ac	170.7, qC			14
23 Ac	171.3, qC			2
CH_3 21	21.3, CH_3	2.17, s		21
CH_3 22	21.6, CH_3	2.02, s		22
CH_3 23	21.7, CH_3	2.02, s		24
OH				

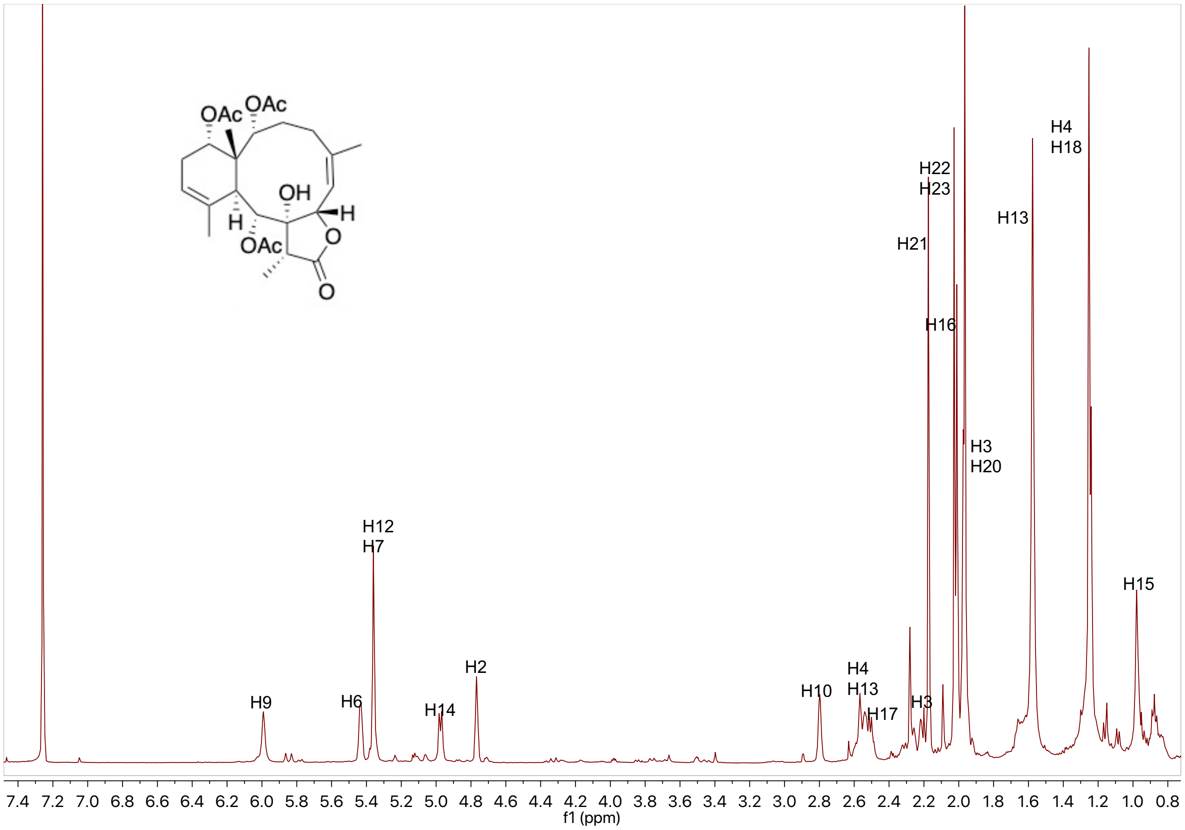

Figure S41. ^{1}H NMR spectrum of lactone 14 (**10**) (500 MHz, $CDCl_3$)

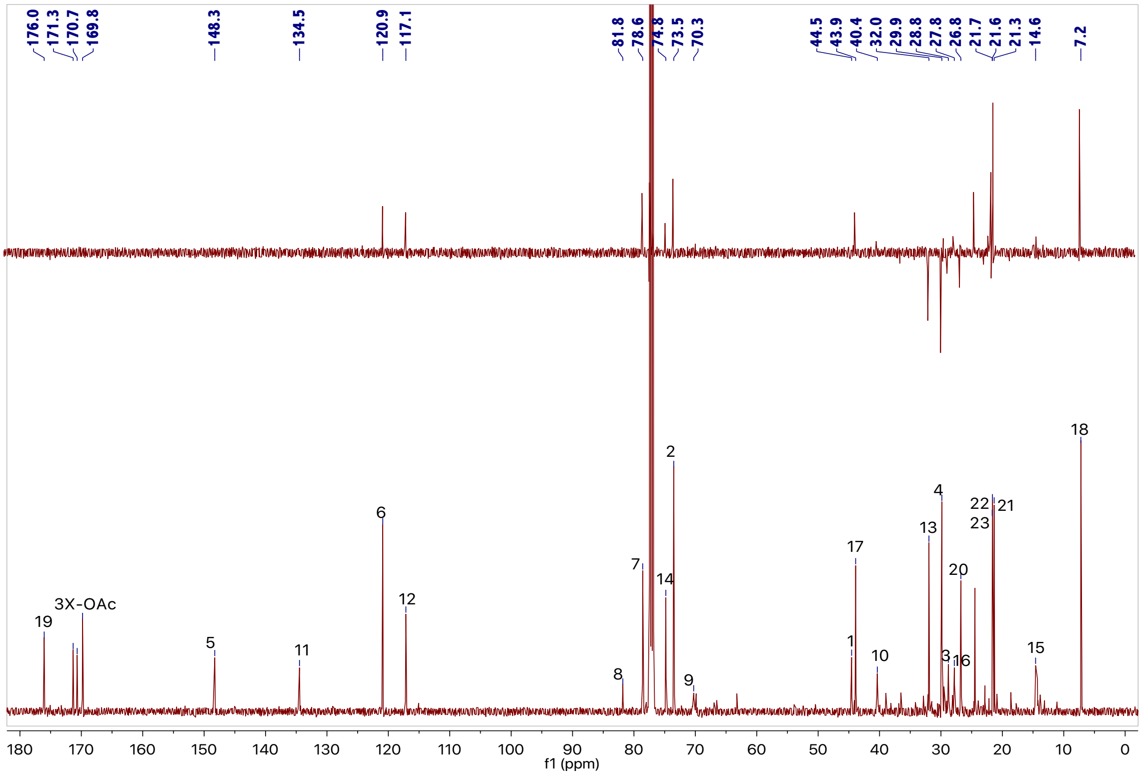

Figure S42. ^{13}C NMR and DEPT-135 spectrum of lactone 14 (**10**) (500 MHz, $CDCl_3$)

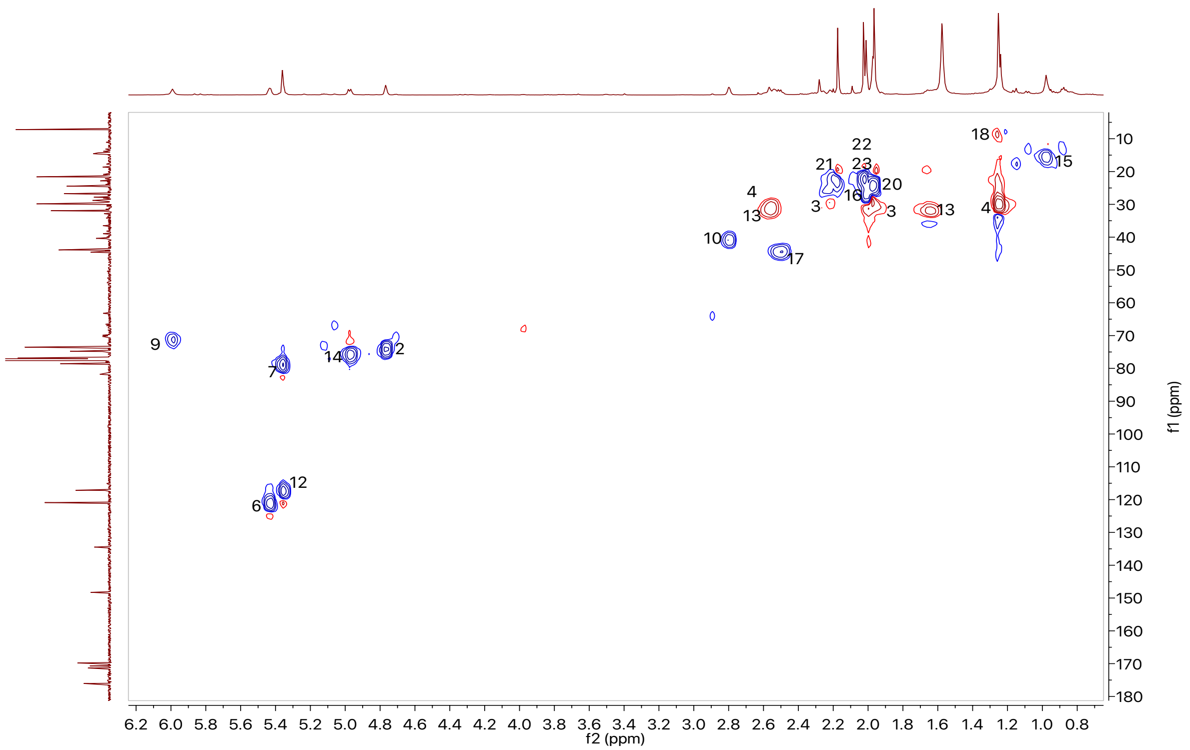

Figure S43. HSQC spectrum of lactone 14 (**10**) (500 MHz, $CDCl_3$). CH_2: red cross-peaks and CH or CH_3: blue cross-peaks.

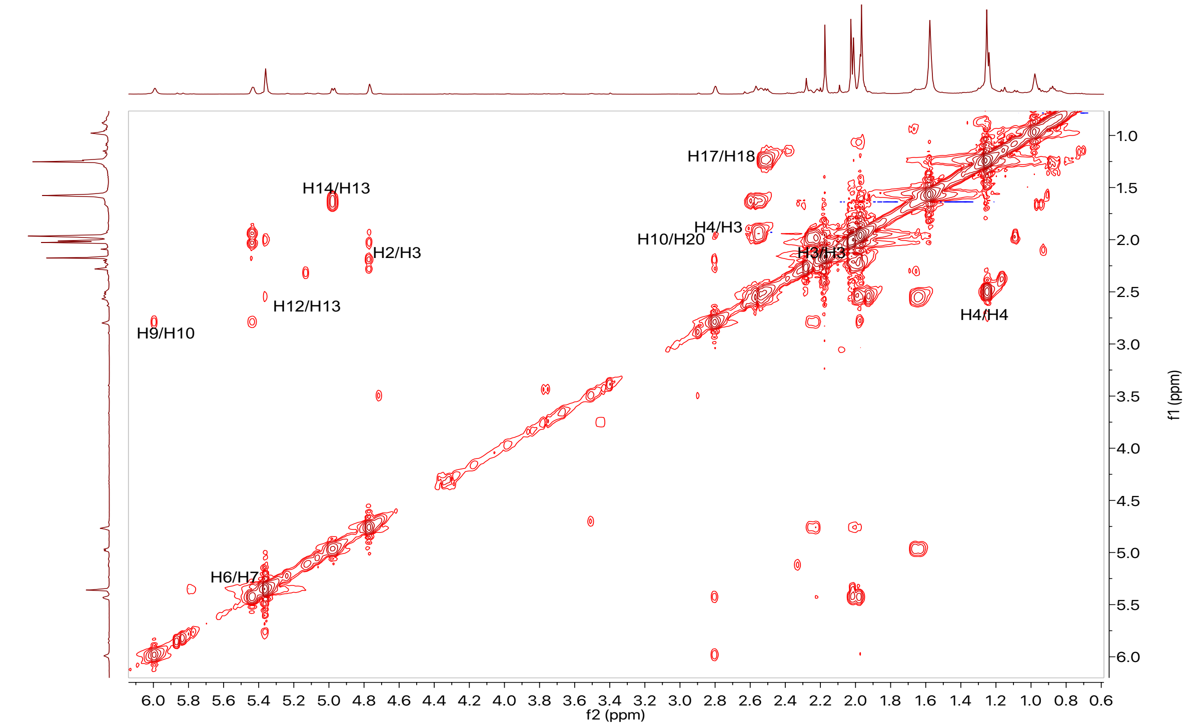

Figure S44. COSY spectrum of lactone 14 (**10**) (500 MHz, $CDCl_3$)

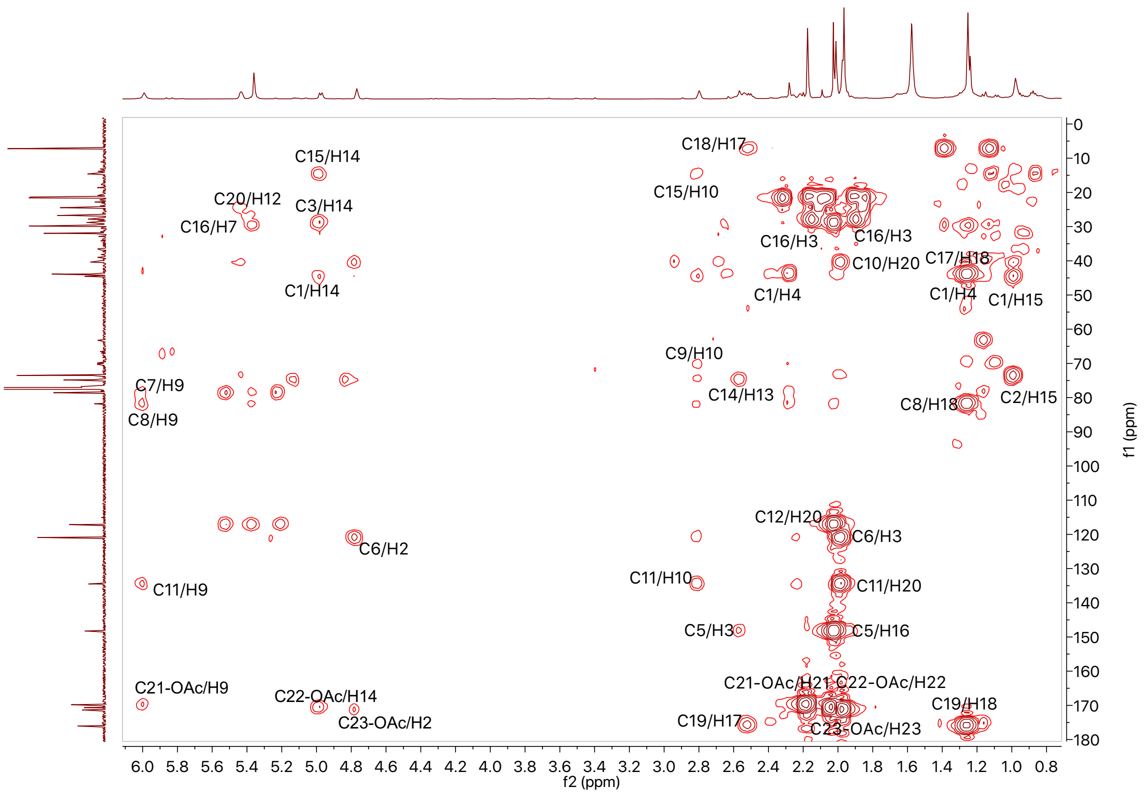

Figure S45. HMBC spectrum of lactone 14 (**10**) (500 MHz, $CDCl_3$)

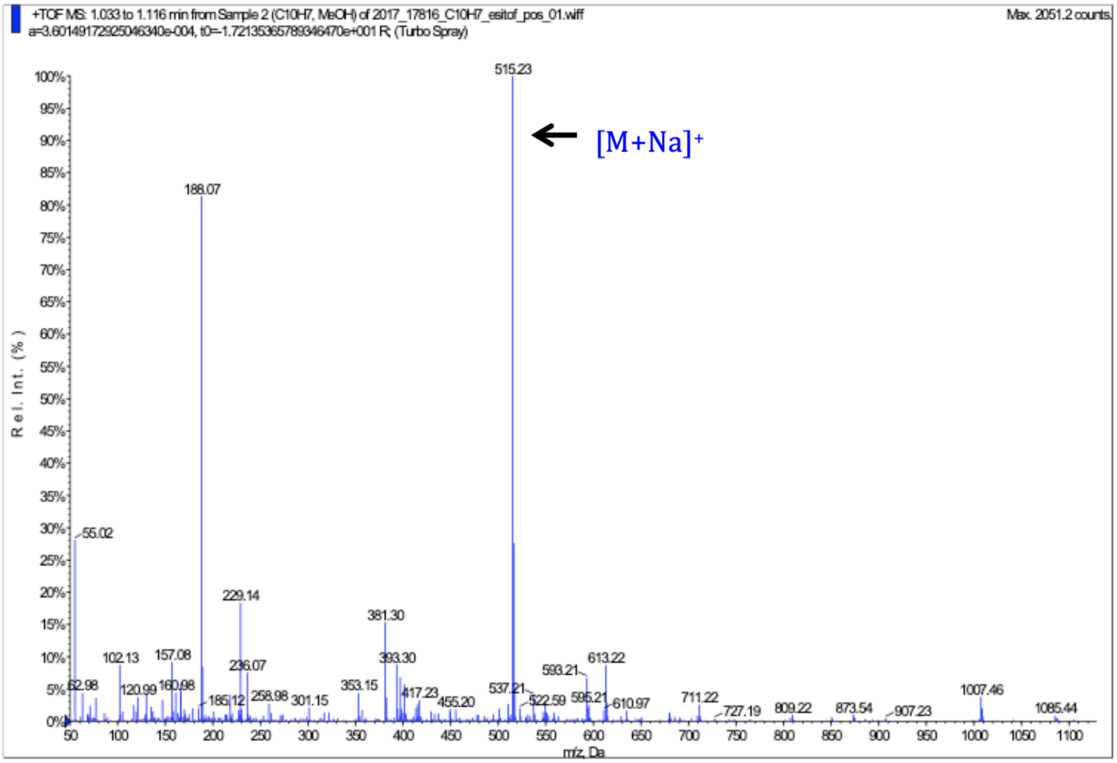

Figure S46. (+)-LRESIMS of lactone 14 **(10)**

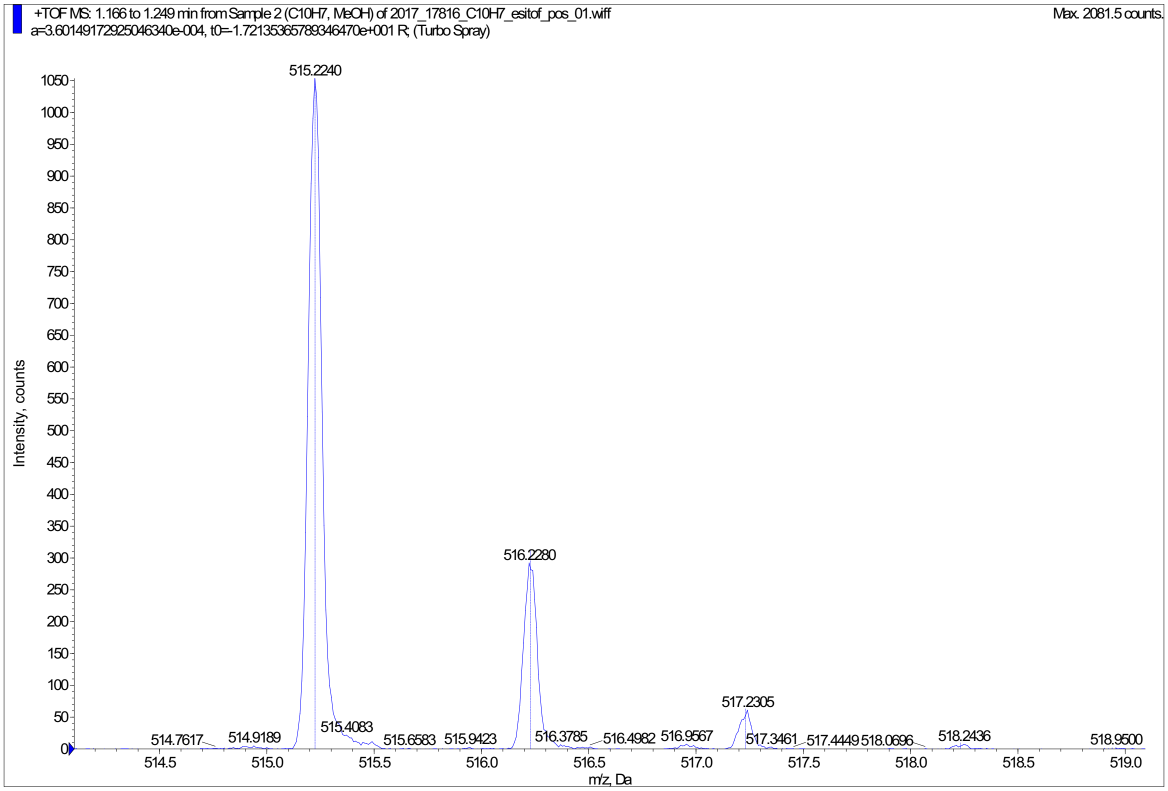

Figure S47. (+)-HRESIMS of lactone 14 (**10**)

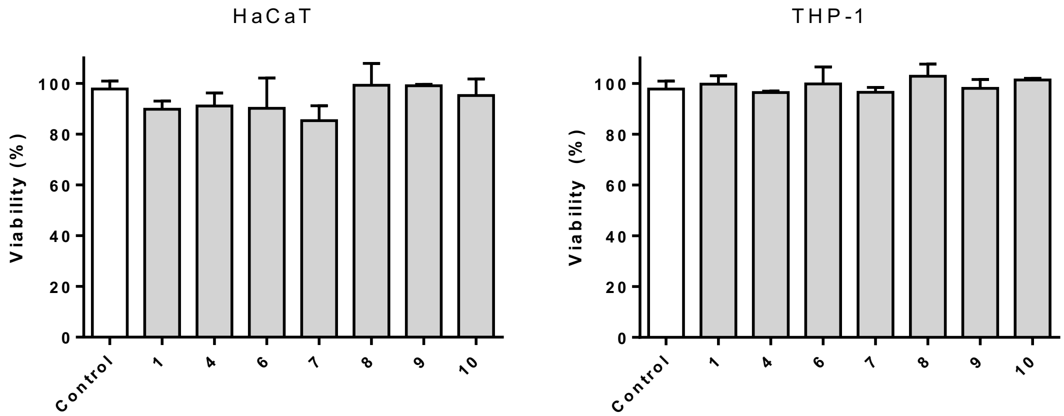

Figure S48. Viability of HaCaT and THP-1 cells exposed to compounds **1**, **4**, **6-10** at 100 μM for 24 hours, as assessed by the MTT assay

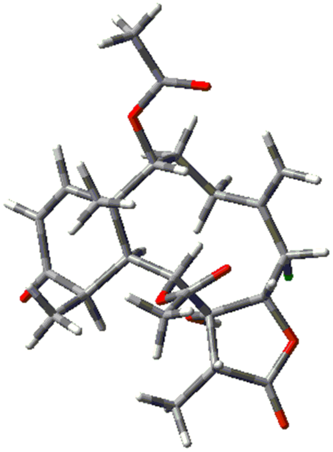

92.8%

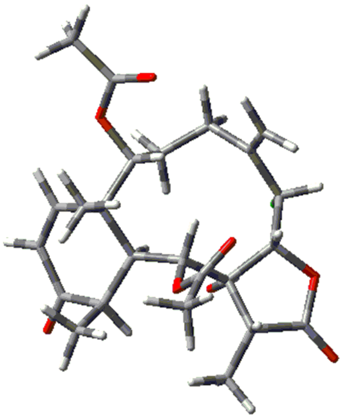

5.1%

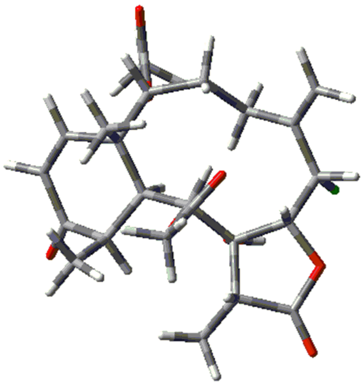

2.1%

Figure S49. The most stable conformers of the (1*S*,2*R*,6*S*,7*R*,8*R*,9*R*,10*S*,11*R*,17*R*) diastereoisomer of briarane B-3.

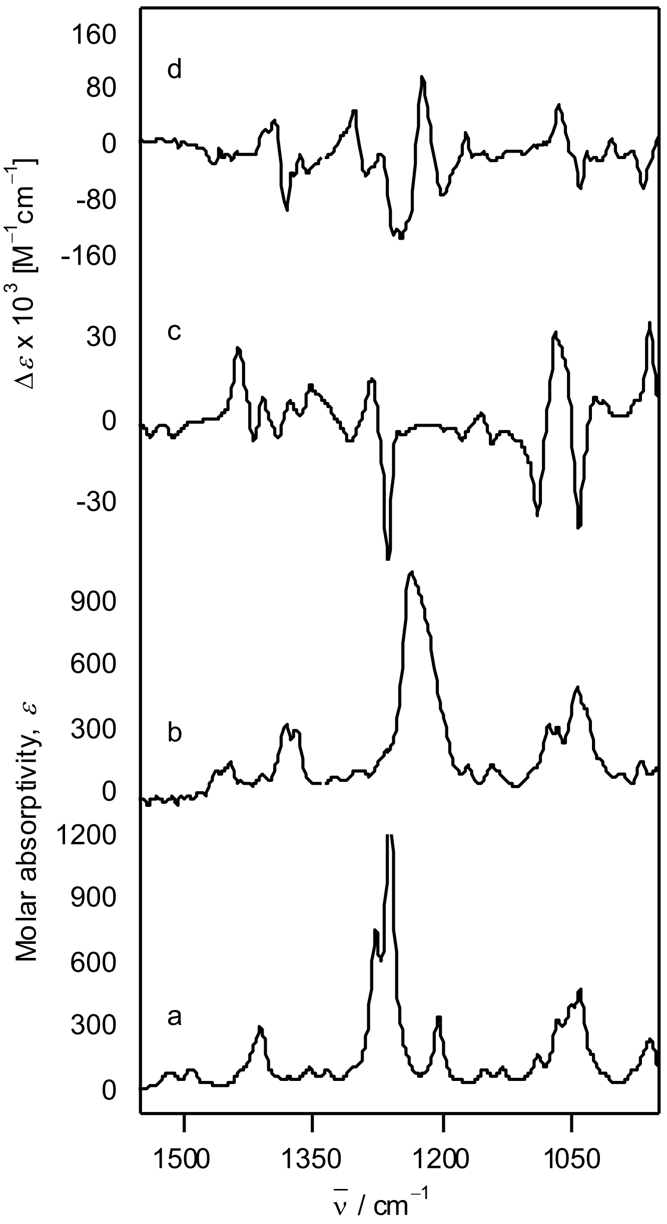

Figure S50. Comparison of the experimental IR (b) and VCD (d) spectra of briarane B-3 (1) with the DFT B3LYP/DGDZVP calculated IR (a) and VCD (c) spectra of its (1*S*,2*R*,6*S*,7*R*,8*R*,9*R*,10*S*,11*R*,17*R*) diastereoisomer.

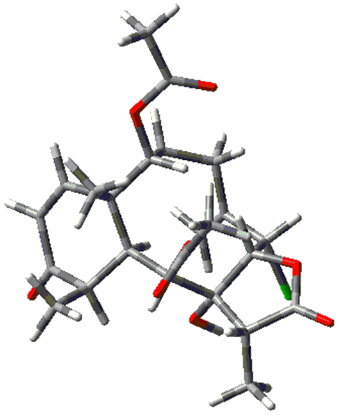

40.1%

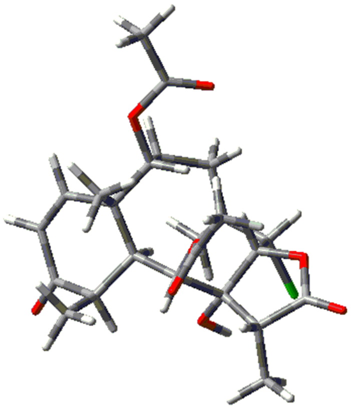

24.5%

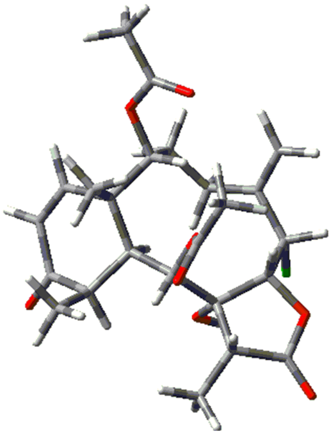

18.0%

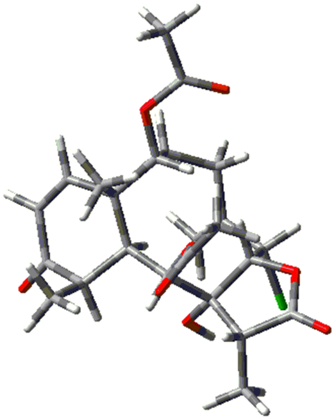

16.5%

Figure S51. The most stable conformers of the (1*S*,2*R*,6*S*,7*R*,8*R*,9*S*,10*S*,11*R*,17*R*) diastereoisomer of briarane B-3.

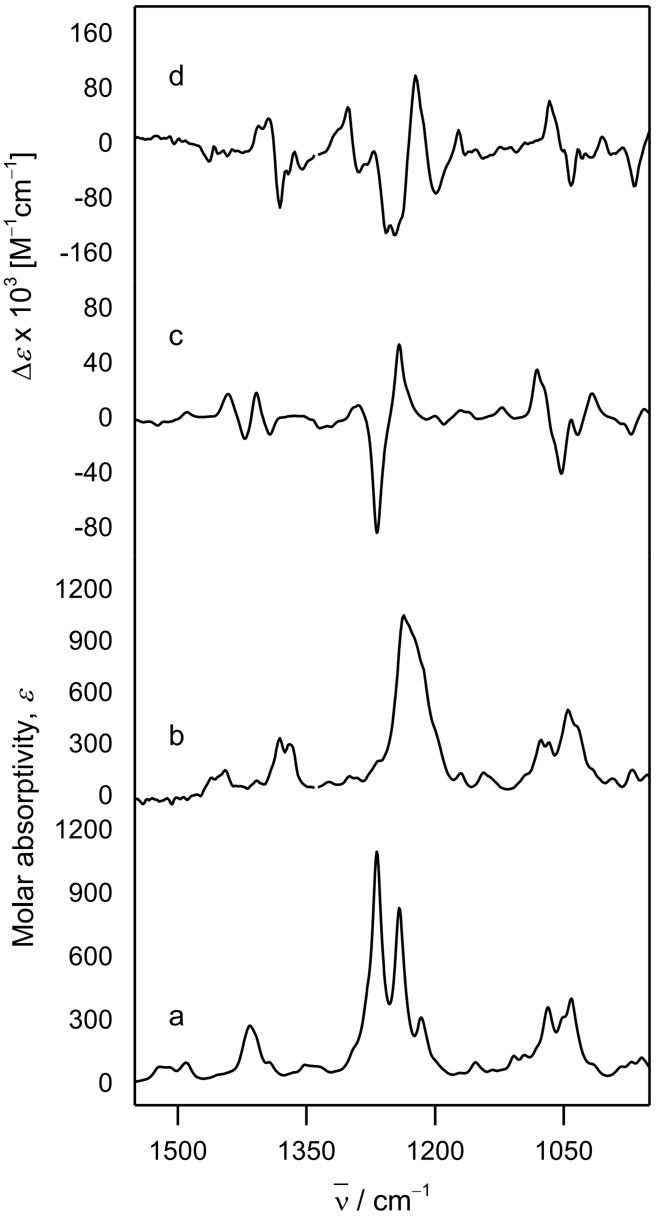

Figure S52. Comparison of the experimental IR (b) and VCD (d) spectra of briarane B-3 (1) with the DFT B3LYP/DGDZVP calculated IR (a) and VCD (c) spectra of its (1*S*,2*R*,6*S*,7*R*,8*R*,9*S*,10*S*,11*R*,17*R*) diastereoisomer.

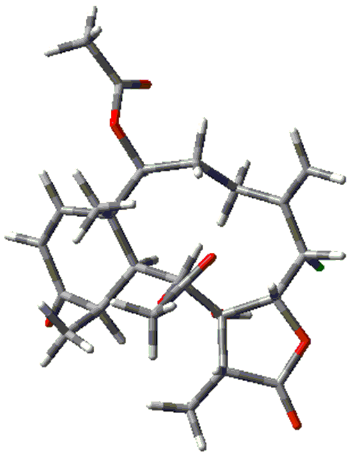

69.5%

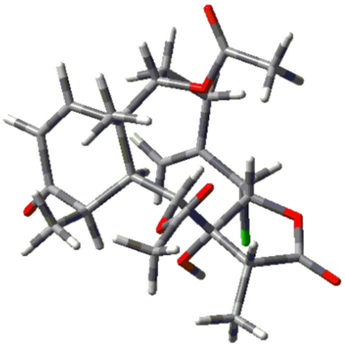

21.6%

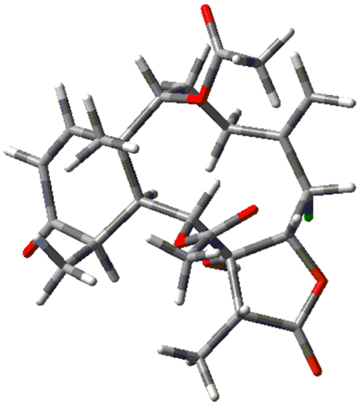

8.9%

Figure S53. The most stable conformers of the (1*S*,2*S*,6*S*,7*R*,8*R*,9*R*,10*S*,11*R*,17*R*) diastereoisomer of briarane B-3.

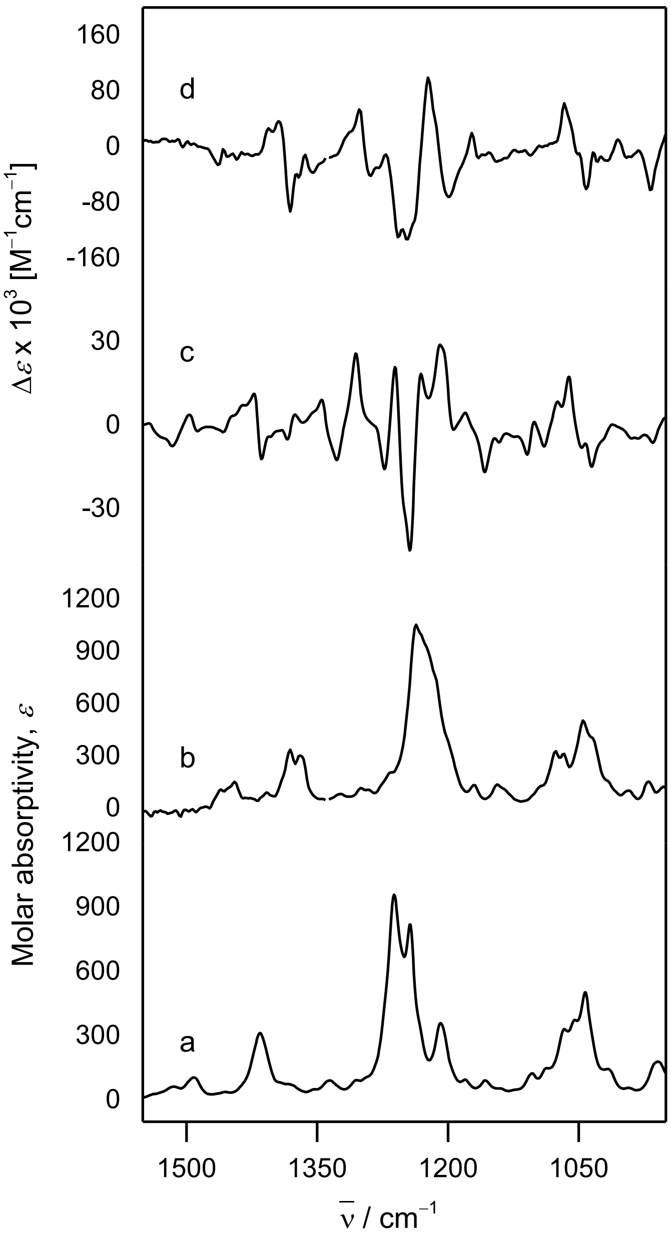

Figure S54. Comparison of the experimental IR (b) and VCD (d) spectra of briarane B-3 (1) with the DFT B3LYP/DGDZVP calculated IR (a) and VCD (c) spectra of its (1*S*,2*S*,6*S*,7*R*,8*R*,9*R*,10*S*,11*R*,17*R*) diastereoisomer.

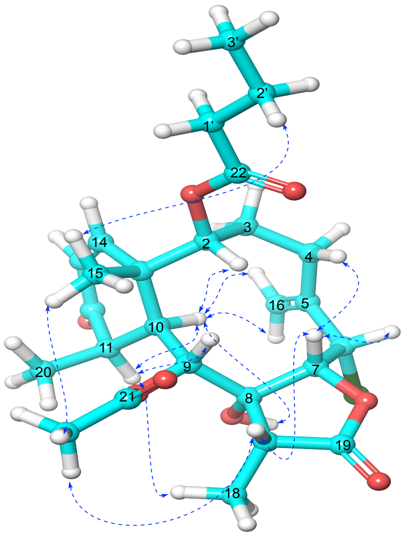

Figure S55. Key NOESY correlations observed in 2-butyryloxybriarane B-3 **(2).**

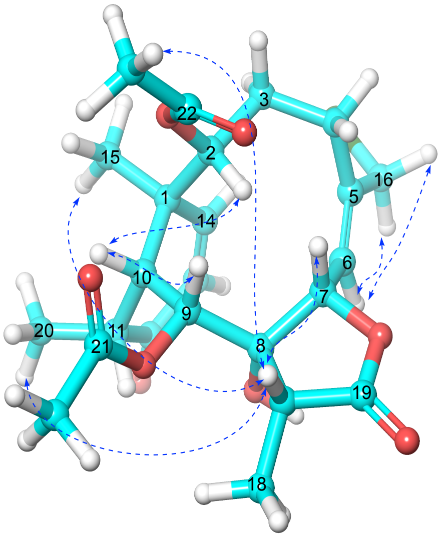

Figure S56. Key NOESY correlations observed in as 9-acetylbriarenolide S (**3**).

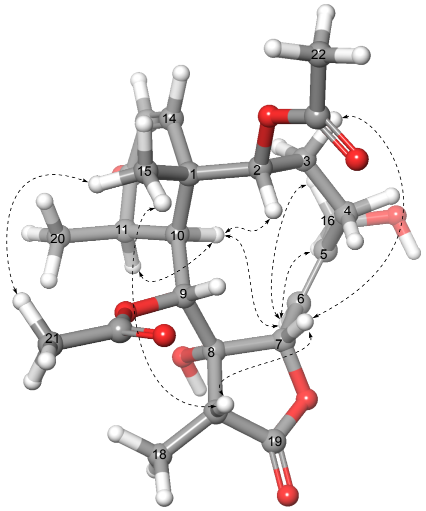

Figure S57. Key NOESY correlations observed in briarenolide W (**4**).

Table S9. qPCR target information.

Gene	Accession number	Primers	Annealing temperature (°C)	Amplicon length (bp)
Ptgs2 (COX-2)	NM_000963.4	F: CCCATGTCAAAACCGAGGTG<br>R: AAAATTCCGGTGTTGAGCAGT	59.0	107
Tnf (TNF-α)	NM_000594.4	F: TAGCCCATGTTGTAGCAAACC<br>R: ATGAGGTACAGGCCCTCTGAT	59.5	136
Il6 (IL-6)	NM_000600.5	F: CTTCTCCACAAGCGCCTTCG<br>R: TGGAATCTTCTCCTGGGGGT	61.0	101
Il1β (IL-1β)	NM_000576.2	F: CCTGAGCTCGCCAGTGAAAT<br>R: TGGAAGGAGCACTTCATCTGTTT	60.5	98
Gapdh (GAPDH)	NM_002046.6	F: AGGTCGGAGTCAACGGATTT<br>R: TGGAATTTGCCATGGGTGGA	60.0	157
